# Synthesis of Fmoc-Triazine Amino Acids and Its Application in the Synthesis of Short Antibacterial Peptidomimetics

**DOI:** 10.3390/ijms21103602

**Published:** 2020-05-20

**Authors:** Pethaiah Gunasekaran, Eun Young Kim, Jian Lee, Eun Kyoung Ryu, Song Yub Shin, Jeong Kyu Bang

**Affiliations:** 1 Division of Magnetic Resonance, Korea Basic Science Institute (KBSI), Ochang 28119, Korea; gunaharaks@kbsi.re.kr (P.G.); paperin@kbsi.re.kr (J.L.); ekryu@kbsi.re.kr (E.K.R.); 2 Department of Medical Science, Graduate School, Chosun University, Gwangju 61452, Korea; lovetive@naver.com (E.Y.K.); syshin@chosun.ac.kr (S.Y.S.); 3 Department of Bio-analytical Science, University of Science & Technology, Daejeon 34113, Korea; 4 Department of Cellular and Molecular Medicine, School of Medicine, Chosun University, Gwangju 61452, Korea

**Keywords:** Fmoc-triazine-amino acids, antibacterials, short peptidomimetics, intracellular targeting, unnatural amino acids

## Abstract

To combat the escalating rise of antibacterial resistance, the development of antimicrobial peptides (AMPs) with a unique mode of action is considered an attractive strategy. However, proteolytic degradation of AMPs remains the greatest challenge in their transformation into therapeutics. Herein, we synthesized Fmoc-triazine amino acids that differ from each other by anchoring either cationic or hydrophobic residues. These unnatural amino acids were adopted for solid-phase peptide synthesis (SPPS) to synthesize a series of amphipathic antimicrobial peptidomimetics. From the antimicrobial screening, we found that the trimer, **BJK-4** is the most potent short antimicrobial peptidomimetic without showing hemolytic activity and it displayed enhanced proteolytic stability. Moreover, the mechanism of action to kill bacteria was found to be an intracellular targeting.

## 1. Introduction

Antibiotics play vital roles in health care since the invention of penicillin. However, frequent and excessive misuse of antibiotics contributes to the escalating threat of antibiotic resistance [1,2]. Worldwide more than 700,000 infections are caused by antibiotic-resistant bacteria, which are identified as the major public health issues of the 21st century [3,4]. Development of a new class of antibiotics in the past two decades has decreased. Even though 38 antibiotics were approved by the Food and Drug Administration (FDA) since 2000 [5,6,7], only five were new classes, in fact, which were active only against Gram-positive bacteria. Thus, a combination of a rapid increase of multidrug-resistant (MDR) pathogens and a noticeable scarcity in the development of a new class of antibiotics necessitate the discovery of new and alternative potent antimicrobial agents [5]. In this regard, antimicrobial peptides (AMPs) attract significant attention as a new generation antibiotics to combat the drug-resistant pathogens because they are widely present in species ranging from bacteria to mammals as a host defense system and exhibit potent antimicrobial activities [6]. In general, AMPs, are a class of amphipathic peptides, comprise of a series of cationic and hydrophobic amino acids that adopt amphipathic structure upon the contact with the microbial membrane, which leads to a distinctive mode of action and rapid killing rate against the pathogens. Thus, propensity for the development of resistance becomes low, eventually enhancing the efficacy for combating multi-drug resistance bacteria [7,8]. Even though AMPs are found to be an alternative for treating drug-resistant bacteria, their transformations into therapeutics is a highly challenging due to the severe disadvantages, including high production cost owing to their large size, hemolytic activity, poor stability in protease and cytotoxicity [9]. In addition, usage of AMPs with sequences that are similar to that of the human defense system, may cause an inevitable loss in the natural defense [10].

Considering the limitations mentioned above, significant efforts have been taken for the synthesis of small molecular mimetics [11,12], polymers [13], arylamide foldamers [14], and dipeptides [15]. In addition, incorporation of unnatural amino acids into biologically active peptides was found to modify its activity and stability [16]. Our group reported short peptidomimetics derived from pyrazole based unnatural amino acids and cationic peptide mimicking aryl antibacterials [17,18]. As a continuous effort to discover the antibacterials [19,20], in the present study, we focused on developing the short and simple peptidomimetics from structurally simple Fmoc-triazine amino acids.

**Figure 1:**
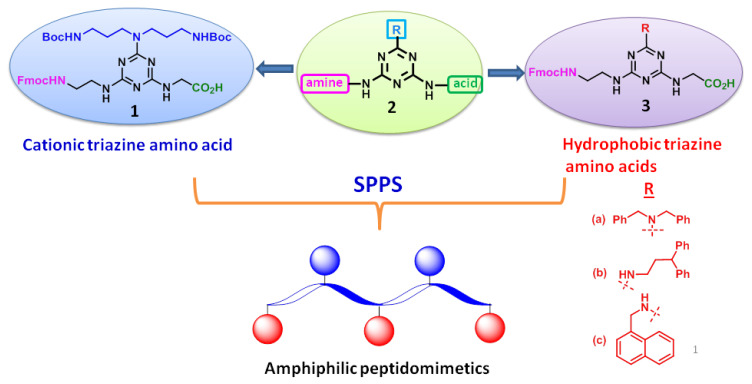
Designing strategy of Fmoc-triazine amino acids. SPPS = solid-phase peptide synthesis.

Triazine heterocycles find considerable attention as novel scaffolds in medicinal chemistry due to their remarkable pharmacological properties. In particular, *s*-triazine under nucleophilic substitution reactions offers diverse and substituent depended structural array of 1,3,5-triazines. Moreover, triazine derivatives were found to exhibit various biological properties along with the antibacterial activity [13,18,21]. In this work, we have designed two types of 1,3,5-triazine based unnatural amino acids holding trisubstitution, including a Fmoc-amine, an acid and a cationic/hydrophobic group, as shown in Figure 1.

Then, we designed and synthesized a series of amphipathic peptidomimetics for the first time from the newly synthesized cationic and hydrophobic Fmoc-triazine amino acids placing at alternative positions using solid-phase peptide synthesis (SPPS). Even though pseudo- and cyclic peptidomimetics [22,23] were derived using triazine building blocks, the present work advances in terms of synthesis of cationic/hydrophobic Fmoc-triazine amino acids and their application in further construction of short, amphipathic antimicrobial peptidomimetics.

The synthesized triazine based peptidomimetics were screened for their antimicrobial activities against a panel of both Gram-positive and Gram-negative bacterial strains. In view of identifying the most promising compound, the trimer **BJK-4** and tetramer **BJK-6** were found to elicit significant antibacterial effects. Moreover, the short peptidomimetic, **BJK-4** was evaluated for proteolytic stability using trypsin digestion method. To understand the bacterial killing mechanism, and to identify the effect of this compound on the integrity of the bacterial membranes and intracellular region, we investigated the cytoplasmic membrane depolarization, membrane permeability, flow cytometry studies. Moreover, the influence of **BJK-4** on the migration of plasmid DNA was also examined.

## 2. Results and Discussion

### 2.1. Design and Synthesis

In search of discovering a unique scaffold for synthesizing peptidomimetics from unnatural amino acids, we investigated the 1,3,5-triazine as a core moiety because it can be derived from cheap and commercially available starting material 2,4,6-trichloro-1,3,5-triazine, i.e., cyanuric chloride. Achieving substrate-defined trisubstitution in cyanuric chloride is highly facile by sequential aromatic nucleophilic substitution reaction using temperature gradient that can offer diversity in structures. Thus, synthesis of triazine based Fmoc amino acids and further construction of amphipathic peptidomimetics are feasible by controlling the substitution. These Fmoc-triazine amino acids may increase the proteolytic stability of the peptidomimetics. Moreover, identification of triazine based short amphipathic peptidomimetics may lead to a significant contribution to the development of AMPs.

**Figure 2:**
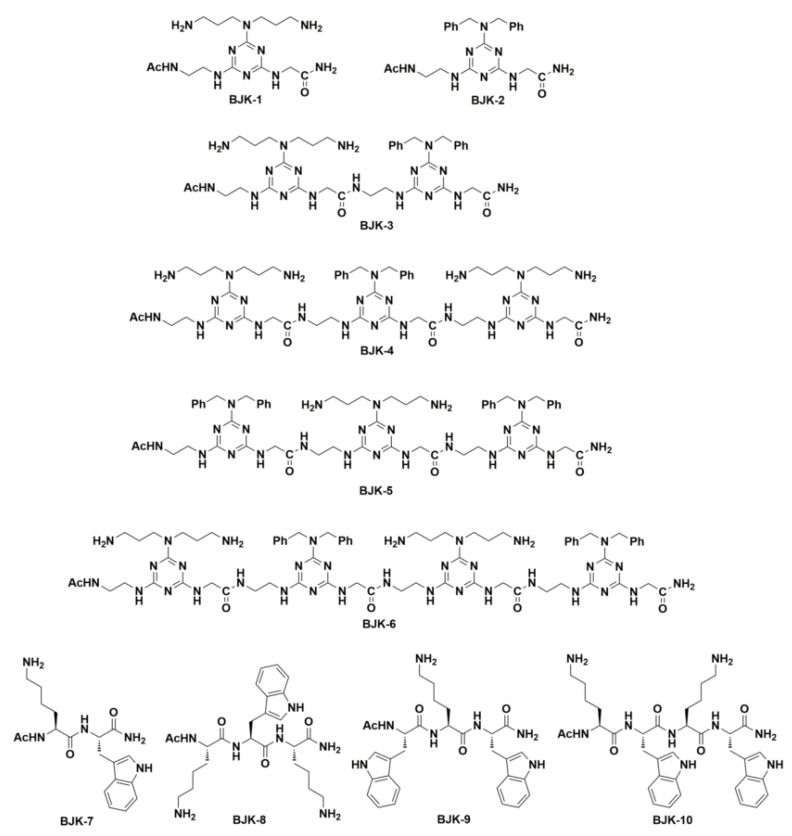
Structures of peptidomimetics derived from the Fmoc-triazine amino acids **BJK-(1-6)** and peptides derived from Lys and Trp, **BJK-(7-10)**.

The antimicrobial peptides consisting of Trp (W) and Lys (K) repeating units showed significant antimicrobial activity [24,25,26], which was attributed to the fact that amphipathicity achieved from (KW)n. We hypothesized that hydrophobic-Fmoc-triazine amino acid might contribute the hydrophobicity similar to that of Trp and cationic Fmoc-triazine amino acid can mimic the Lys. Thus, we have synthesized four peptides involving Trp (W) and Lys (K) (Figure 2) and these amphipathic peptides were compared with the triazine-derived peptidomimetics for antimicrobial activity.

In our design, triazine scaffold, **2** was appended with an acid and a Fmoc-amine functionality that are identical for both cationic and hydrophobic amino acids. However, these amino acids are differed at the third substituent by anchoring either cationic or hydrophobic residues, as shown in Figure 1. *tert*-butyl *N*-[3-[3-[(2-methylpropan-2-yl)oxycarbonylamino]propylamino]propyl]carbamate (**4**) was appended for deriving cationicity. Moreover, dibenzylamine, 3,3-diphenylpropylamine and 1-naphthylmethylamine were anchored for obtaining hydrophobicity.

**Scheme 1:**
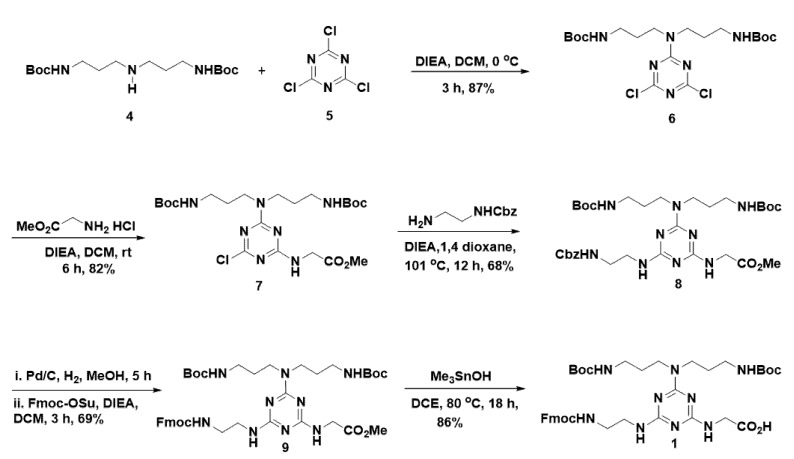
Synthesis of cationic Fmoc-triazine amino acid.

With this aim in mind, we set out to develop a suitable route for the synthesis of **1** as delineated in Scheme 1. Thus, we performed a sequential introduction of appropriate nucleophiles in cyanuric chloride using a temperature gradient. Initially, as a cationic precursor, we appended *tert*-butyl *N*-[3-[3-[(2-methylpropan-2-yl)oxycarbonylamino]propylamino]propyl]carbamate (**4**) in the first nucleophilic substitution reaction in the presence of *N*,*N*-diisopropylethylamine (DIEA) at 0 °C to result in the formation of **6**. Glycine methyl ester was used as the second nucleophile to react with **6** in the presence of DIEA in dichloromethane under room temperature to yield **7** in 82% yield. In order to introduce Fmoc group in the third substitution, mono protected diamine was required, indeed that protection must be capable for deprotection under neutral condition because acid-sensitive Boc groups and base sensitive methyl ester are present in **7**. Considering these facts, we chose the Cbz-ethylenediamine, which was treated with **7** in 1,4-dioxane and heated to reflux at 101 °C to result in the formation of **8** in 68% of yield. In order to introduce the Fmoc group, initially, Cbz-group of **8** was deprotected using Pd/c in hydrogen atmosphere in MeOH, and subsequent treatment with Fmoc-OSu in the presence of DIEA in dichloromethane yielded the **9** in 69% of yields. In mid of acid-sensitive Boc group and base sensitive Fmoc in **9**, selective methyl ester hydrolysis was found highly challenging. Thus, we performed methyl ester hydrolysis in neutral conditions using LiI in ethyl acetate [17], unfortunately, it was not fruitful. Next, we attempted for selective methyl ester hydrolysis of **9**, using Me_3_SnOH in 1,2-dichloroethane (DCE) at 80 °C following the K. C. Nicolaou et al. protocol [27]. Despite the catalyst loading was high, interestingly, the Me_3_SnOH mediated reaction offered a selective methyl ester hydrolysis to yield the desired cationic Fmoc-triazine amino acid **1** in 86% of yields, without affecting sensitive protective groups such as Boc and Fmoc. Thus, our work describes the synthesis of cationic Fmoc-triazine amino acid, which possesses a Fmoc amine and a carboxylic acid, which are amenable for SPPS protocols for peptide synthesis, from commercially available and cheap starting material involving five steps.

**Scheme 2:**
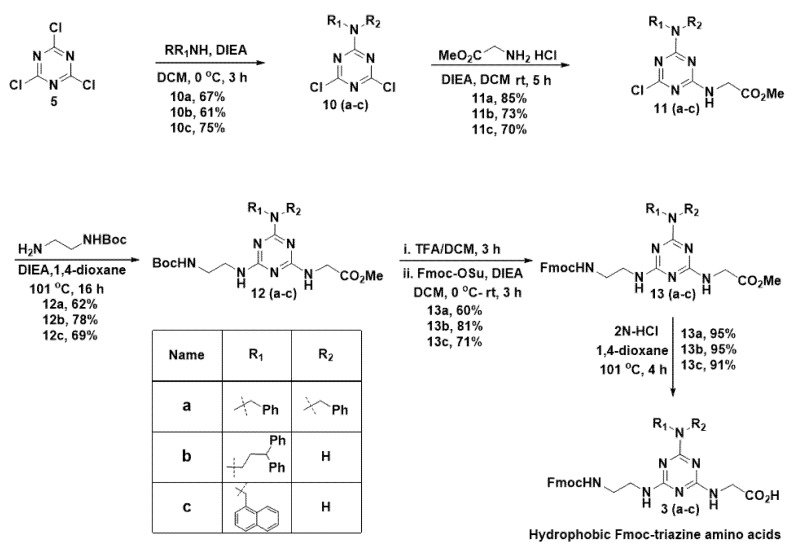
Synthesis of hydrophobic Fmoc-triazine amino acids.

Next, in the synthesis of hydrophobic Fmoc-triazine amino acids, initially, we planned to anchor functionalities that were responsible for hydrophobicity in the first substitution reaction in cyanuric chloride (Scheme 2). Thus, we treated various amines, including dibenzylamine, 3,3-diphenylpropylamine and 1-naphthylmethylamine with cyanuric chloride (**5**) in the presence of DIEA at 0 °C in dichloromethane that resulted in the formation of **10(a-c)** in 61–75% yields; however, traces of disubstituted product formation was also inevitable.

In the second nucleophilic substitution reaction, the acid precursor glycine methyl ester was planned to append. Consequently, glycine methyl ester was treated with **10(a–c)** at room temperature in the presence of DIEA to obtain **11(a–c)** in 70–85% yields, and bi-product formation was limited in this reaction. For the third substitution reaction, Boc-ethylenediamine was used as a precursor for appending the Fmoc group in the next stage. Thus, Boc-ethylenediamine was treated **11(a–c)** in the presence of DIEA under reflux condition in 1,4-dioxane to result in the formation of **12(a–c)** in 62–78% yields. To introduce the Fmoc group at **12(a–c)**, the Boc group was deprotected from the amine in **12(a–c)** using TFA in dichloromethane. After evaporating the solvent, the crude residue was treated with Fmoc-OSu in the presence of DIEA in dichloromethane to result in compound, **13(a–c)** in 60–71% yields. Finally, hydrolysis of methyl ester was carried out using 2N HCl in refluxing 1,4-dioxane to get the desired hydrophobic Fmoc-triazine amino acid **3(a–c)**, in 91–95% yields. Thus, our methodology offers versatility in the synthesis of hydrophobic Fmoc-triazine amino acids from commercially available cheap starting material with simple operational procedures.

After the successful synthesis of cationic and hydrophobic Fmoc-triazine amino acids, they were adopted for the sequence controlled amphipathic triazine peptidomimetics synthesis using Rink amide resin mediated SPPS. In particular, in this work, we synthesized six amphipathic peptidomimetics, **BJK-(1-6)**, using dibenzyl anchored hydrophobic Fmoc-triazine amino acid **3a** and cationic Fmoc-triazine amino acid (**1**) as shown in Figure 2. It is pertinent to note that *N*-acetyl protection and *C*-terminal amidation in AMPs tends to increase the proteolytic stability [28]. In view of increasing proteolytic stability, acetyl, and amidation were protected at *N*-terminal and *C*-terminals of all the synthesized peptidomimetics, respectively. Further, peptides, **BJK**-**(7**-**10)** involving the sequences of Lys and Trp were also synthesized. For the coupling, 2-(1*H*-benzotriazole-1-yl)-1,1,3,3-tetramethyluronium hexafluorophosphate (HBTU) and 1-hydroxybenzotriazole (HOBt) in DMF were used, and the Fmoc group removal was facilitated with 20% piperidine in DMF. After the completion of the sequence, finally, to cleave the peptide from the resin, the mixture of trifluoroacetic acid, water, and triisopropylsilane (90:5:5, *v*/*v*/*v*, 2 mL) was treated and precipitated from diethyl ether. The resultant crude peptidomimetics and peptides were purified using preparative RP-HPLC. The molecular weight of synthesized peptidomimetics and peptides was determined by matrix-assisted laser desorption ionization time-of-flight mass spectrometry (MALDI-TOF MS).

### 2.2. Structure Antimicrobial Activity Relationship Study (SAR)

Having synthesized the amphipathic peptidomimetics, we evaluated the antimicrobial activities of these peptidomimetics against a panel of both Gram-positive and Gram-negative bacterial strains, with the reference, melittin, which is a 26-aminoacid sequenced antimicrobial peptide obtained from venom of European honeybee, Apis mellifera [29,30]. As shown in Table 1, inspired by antibacterial efficiencies of cationic small-molecule antibacterial [18], initially we synthesized monomers **BJK-1** and **BJK-2** from **1** and **3a** respectively, unfortunately, they failed to reveal considerable activity against all the strains. Then, combining both **1** and **3a** led to the formation of the dimer, **BJK-3**, which showed a substantial increase in potency against all the strains compared to that of the monomers. However, except against *Escherichia coli*, **BJK-3** showed a two-fold reduced potency against the other three bacterial strains compared to that of reference, melittin. In contrast, the dimer **BJK-7** sequencing KW did not show any antibacterial activity. Inspired by this encouraging result, further, we were interested in the construction of trimers **BJK-4** and **BJK-5**. In view of understanding the charge effect, **BJK-4** was synthesized involving a sequence that constitutes a hydrophobic residue flanked in between two cationic residues, with increase of charge from **BJK-3**. Surprisingly, **BJK-4** showed the most potent activity against all the bacterial strains similar to that of melittin. It was inferred from the results of **BJK-3** and **BJK-4** that the excess of charge was required for achieving anti-bacterial effect possibly because the extra charge might bind with phospholipids at the surface of the bacterial membrane. Further, to understand the influence of hydrophobicity, **BJK-5** was synthesized with a sequence comprising two hydrophobic and a cationic triazine residue. Unfortunately, **BJK-5** did not show a considerable increase in antibacterial effect compared to that of dimer **BJK-3**, and it lost the antimicrobial effect completely against *E. coli*. In the case of *Pseudomonas aeruginosa*, it displayed a two-fold decreased activity and against Gram-positive strains, four folds reduced activity was found compared to that of reference, melittin. In addition, it is pertinent to note that similarly patterned natural peptides **BJK-8** (KWK) and **BJK-9** (WKW) did not show considerable antibacterial effect compared to that of **BJK-4** and **BJK-5**. In general, a balance between charge and hydrophobicity is an essential factor for achieving antimicrobial activity [31]. Thus, hydrophobicity was increased by the addition of a hydrophobic Fmoc–triazine amino acid in **BJK-4**, which led to the formation of **BJK-6**. Surprisingly, there was no appreciable change in the antibacterial activity of **BJK-6** and it showed an antibacterial effect as same as the most potent peptide, **BJK-4**. This could be attributed to the fact that the cationic charge might interact with negatively charged DNA molecules through electrostatic interactions. Thus, we performed the influence of **BJK-4** on migration of plasmid DNA through the agarose gel in the mechanistic investigation.

This result suggested that an increase in hydrophobicity did not play crucial roles in the case of Fmoc-triazine amino acids. Meanwhile, tetramer from the natural peptide, **BJK-10** (KWKW) failed to show antibacterial effects against any of the tested strains. It is pertinent to note that the peptide, **BJK-10** screening against various strains did not reveal a considerable antibacterial effect [24]. Collectively, we identified **BJK-4** as the most potent short antimicrobial peptidomimetic. It is inferred from the above results that Fmoc-triazine amino acids sequenced peptidomimetics showed significant potential in displaying antibacterial effects compared to that of peptides derived from natural amino acids, including Lys and Trp. The high cationic charges of **BJK-4** and **BJK-6** reveals that cationic charge plays a crucial role in achieving the antibacterial activity. In particular, the short, cationic, trimer peptidomimetic (**BJK-4**) is capable of displaying potent antimicrobial activity.

### 2.3. Hemolytic Activity

**Figure 3:**
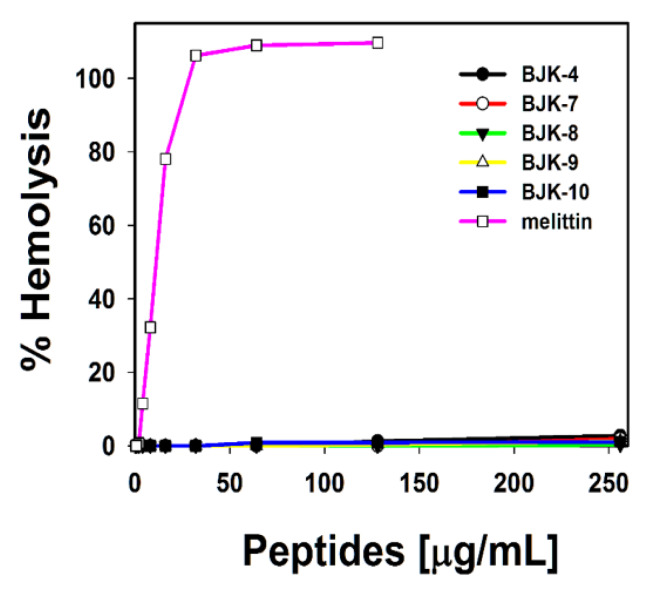
Hemolytic activity of **BJK-4**, **BJK-(7-10)** and melittin against sheep red blood cells (sRBCs).

It is understood from the properties of antibacterial agents that hydrophobicity tends to enhance the hemolysis [32,33]. Since, the short, amphipathic antimicrobial peptidomimetic, **BJK-4** consists of a hydrophobic group, which may trigger the hemolysis; thus, we investigated the hemolysis of **BJK-4**, as shown in Figure 3. The hemolysis results suggested that the short peptidomimetic, **BJK-4** did not induce any hemolytic activity even at the maximum concentration (256 µg/mL). However, melittin displayed maximum hemolysis at 25 µg/mL. These results suggest that **BJK-4** has significant potential to be a model for developing an antimicrobial agent.

#### Cytotoxicity

**Figure 4:**
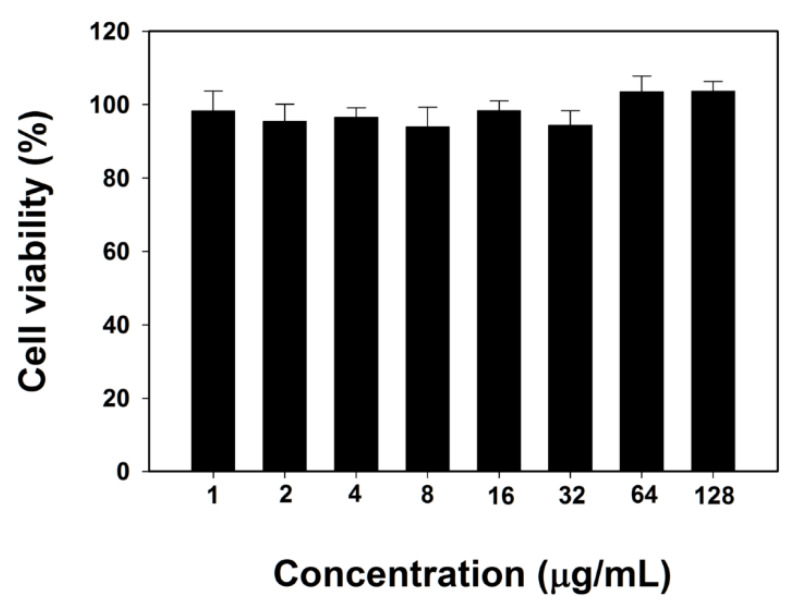
Cytotoxicity of **BJK-4** against RAW 264.7 cells. Data are mean ± SD (*n* = 3).

To evaluate the cytotoxicity of **BJK-4** against mammalian cells, we performed MTT (3-(4,5-dimethylthiazol-2-yl)-2,5-diphenyltetrazolium bromide) dye reduction assay against RAW 264.7 cells as previously described [34]. Interestingly, as shown in Figure 4, **BJK-4** did not show any toxicity even at the maximum concentration of 128 µg/mL. The low toxicity with potent antibacterial activity of **BJK-4** may represent a potential model for designing triazine based peptidomimetic antibacterials.

### 2.4. Protease Stability

**Figure 5:**
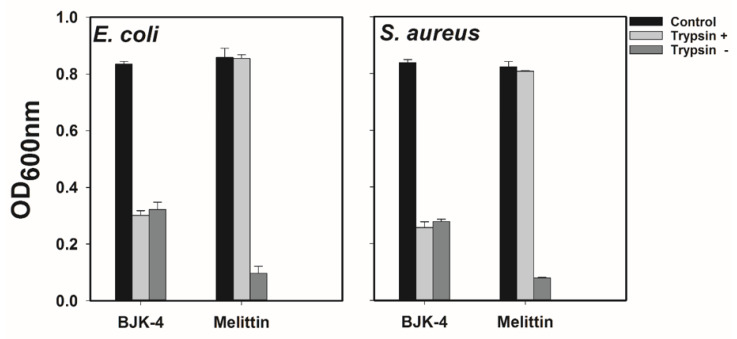
Effect of trypsin on the antimicrobial activity of **BJK-4** and Melittin. *E. coli* KCTC 1682 and *S. aureus* KCTC 1621 strains were used. Control represents bacteria alone.

Susceptibility to protease degradation is one of the serious issues in the advancement of antimicrobial peptides that are sequenced with natural amino acids. As our synthesized peptidomimetics are considered an alternative to natural peptides, we were curious to investigate the proteolytic stability of our peptidomimetics. Trypsin is the most prevalent enzyme in the digestive tract of mammals, which degrades the AMPs that comprise Lys and Arg [35]. Since the most potent peptidomimetic, **BJK-4**, consists of Lys type alkyl amines, we tested its protease resistance ability against trypsin in *E. coli* and *S. aureus* (Figure 5). Trypsin treatment completely abolished the antimicrobial activity of melittin against both *E. coli* and *S. aureus*. In contrast, the antimicrobial activity of **BJK-4** was nearly preserved even after trypsin treatment, proving the resistance to tryptic degradation.

### 2.5. Mechanism of Antimicrobial Action

**Figure 6:**
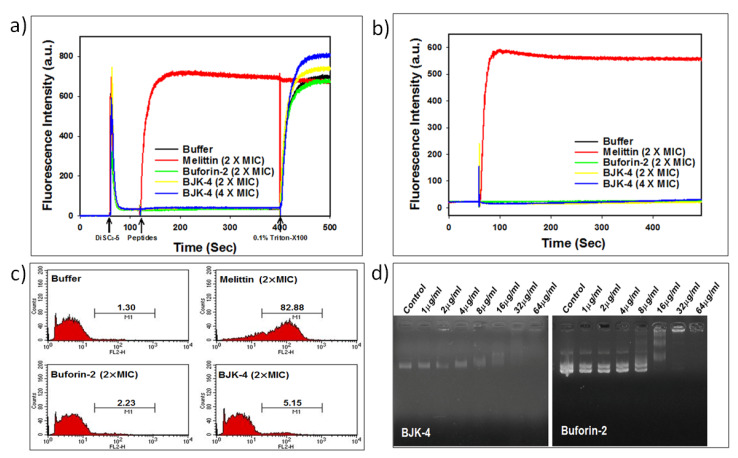
(**a**) Determination of cell membrane depolarization of *S. aureus*, KCTC 1621 using the membrane potential-sensitive fluorescent dye DiSC3-5. (**b**) Cell membrane permeability of *S. aureus*, KCTC 1621 using SYTOX Green assay. (**c**) Cell membrane permeabilization of *E. coli*, KCTC 1682 using Flow cytometry. (**d**) The influence of **BJK-4** and buforin-2 on the migration of plasmid DNA through the agarose gel.

To probe the mechanism of antimicrobial action of **BJK-4** on microbes, we investigated the effect **BJK-4** on the cytoplasmic membrane of bacterial cells using membrane potential sensitive dye DiSC_3_-5 that shows an increase in fluorescence intensity due to the dispersion of probe into the medium during the permeabilization and disruption of the cytoplasmic membrane. Besides, the membrane-disrupting AMP, melittin and intracellular-targeting AMP, buforin-2 were used as references (Figure 6a). Buforin-2, is a 21-amino acid sequencing cationic amphipathic peptide that tends to bind with DNA and RNA to inhibit the cellular functions, are derived from 39-amino acid sequenced AMP, buforin-1 isolated from stomach tissue of Asian toad Bufo gargarizans [36,37,38]. The depolarization by 2× or 4× MIC of the **BJK-4** suggested that it did not induce considerable intensity like buforin-2, while melittin showed an increase in fluorescence intensity. Further, SYTOX Green assay was performed to assess the membrane targeting ability of **BJK-4** (Figure 6b). SYTOX Green is a nucleic acid-binding dye that shows increase in fluorescence intensity during the penetration into bacteria through damaged cell walls. The assay results suggest that **BJK-4** did not increase fluorescence intensity at 2× and 4× MIC as buforin-2 (Figure 6b), in contrast, membrane targeting AMP, melittin shows significant fluorescence intensity. These two results inferred that **BJK-4** might follow the intracellular targeting mechanism similar to that of buforin-2. Further, to ascertain this fact, we performed flow cytometry using *E. coli* in the presence of the DNA intercalating dye PI (Figure 6c). After treatment with 2× MIC melittin, the percentage of PI-positive *E. coli* increased to 82.88%. However, treatment with 2× MIC of buforin-2 resulted only 2.23% of positive nucleic acid staining. Treatment of **BJK-4** at 2× MIC induced 5.15% of cells. Thus, similar to buforin-2, the percentage of PI-positive *E. coli* cells was very low, indicating that **BJK-4** does not target the bacterial cell membranes but kills the bacteria by the intracellular-target mechanism. Finally, to investigate the influence of **BJK-4** on plasmid DNA, retardation of DNA by **BJK-4** and buforin-2 was assessed by analyzing the electrophoretic movement of plasmid DNA bands through an agarose gel (1%, *w*/*v*). Like buforin-2, **BJK-4** was capable of inhibiting DNA migration at a concentration of 16 μg/mL (Figure 6d), which suggests that **BJK-4** kills bacteria possibly by inhibiting intracellular functions via interference with DNA function.

## 3. Materials and Methods

### 3.1. Chemistry

As described previously [39], all reactions were performed under argon atmosphere in flame-dried glassware using dry solvents unless otherwise noted. Anhydrous organic solvents of purity greater than 99.9% were purchased from Aldrich and used directly in the reaction. All reagents and some of the starting materials were purchased from commercial chemical suppliers, including Sigma-Aldrich, TCI, and Across Organics and used as received. Analytical thin-layer chromatography (TLC) was performed on Merck aluminum sheets with silica gel 60 F254 using 0.25 mm plates, and was visualized by ultraviolet light, staining with KMnO_4_ and ninhydrin. Column chromatography purification was performed on Merck silica gel 60 (70–230 mesh or 230–400 mesh). Bruker DRX-400 and DRX-500 NMR spectrometer were used for recording ^1^H and ^13^C NMR spectra. NMR chemical shifts (δ) are denoted in parts per million (ppm), and coupling constants (*J*) are given in hertz (Hz). MALDI-TOF mass was recorded using Shimadzu mass spectrometer. The crude peptidomimetics and peptides were purified by reverse-phase high-performance liquid chromatography (RP-HPLC) on Agilent HPLC system equipped with a C_18_ semi-preparative column (250 mm × 22 mm, 10 μm) using 0.05% aq. trifluoroacetic acid (TFA) (eluent A) and 0.05% TFA in CH_3_CN (eluent B) were used with a flow rate of 5.0 mL/min at 25 °C, under wavelength 280 nm. Moreover, the purity of the peptide judged to be ≥ 95% pure by analytical HPLC equipped with a C_18_ column (4.6 mm× 250 mm, 10 μm). Two different linear gradients of 0.05% aq. TFA (eluent A) and 0.05% TFA in CH_3_CN (eluent B) (gradient method, 5% of eluent B to 95% of Eluent B in 30 min; injection volume, 10 µL; concentration 10 µmol) were used with a flow rate of 1.0 mL/min at 25 °C, under wavelength 280 nm.

#### 3.1.1. *tert*-Butyl 3,3’-(4,6-dichloro-1,3,5-triazin-2-ylazanediyl)bis(propane-3,1-diyl)dicarbamate (**6**)

To a stirred solution of cyanuric chloride **5** (1 g, 5.43 mmol) and DIEA (2.8 mL, 16.3 mmol,) in CH_2_Cl_2_ (30 mL) at 0 °C, was added **4** (1.83 g, 5.54 mmol) in CH_2_Cl_2_ (20 mL) dropwise over 30 min, ensuring that the temperature was maintained at 0 °C, and stirred further for 3 h at 0 °C. The reaction mixture was quenched with water (50 mL) and extracted from CH_2_Cl_2_ (2 × 30 mL). The combined organic layer extracts were washed with brine (50 mL), dried over Na_2_SO_4_, and evaporated. The resultant crude residue was purified by silica gel column chromatography (hexane-EtOAc, 3:1) to afford **6** as a white solid (2.26 g, 87%). ^1^H NMR (400 MHz, CDCl_3_) δ 5.05 (t, *J* = 6.0 Hz, 2H), 3.63 (t, *J* = 6.9 Hz, 4H), 3.13 (q, *J* = 6.4 Hz, 4H), 1.81 (p, *J* = 6.7 Hz, 4H), 1.45 (s, 18H). ^13^C NMR (100 MHz, CDCl_3_) δ 170.1, 164.7, 156.0, 79.3, 45.0, 37.4, 28.4, 27.7. Maldi-tof m/z calcd for C_19_H_32_Cl_2_N_6_O_4_: 478.1, found 501.2 (M+Na)^+^.

#### 3.1.2. Methyl 2-((4-(bis(3-((*tert*-butoxycarbonyl)amino)propyl)amino)-6-chloro-1,3,5-triazin-2-yl)amino)acetate (**7**)

To a stirred solution of **6** (1.4 g, 2.92 mmol) and DIEA (2.96 mL, 14.6 mmol) in CH_2_Cl_2_ (20 mL), was added glycine methyl ester hydrochloride (0.73 g, 5.84 mmol) slowly. The resultant solution was stirred for 6 h at room temperature and then quenched with H_2_O (50 mL). The resultant mixture was extracted from CH_2_Cl_2_ (2 × 20 mL). The combined organic layers were washed with brine (20 mL), dried over anhydrous Na_2_SO_4_, filtered, and evaporated. The residue was purified by flash chromatography on silica gel using hexane/EtOAc (1:1) to afford the title compound (**7**) (1.27 g, 82%) as a white solid. ^1^H NMR (500 MHz, CDCl_3_) δ 6.16 (s (br), 1H), 5.51 (s (br), 1H), 4.95 (s, (br), 1H), 4.28–4.12 (m, 2H), 3.85–3.75 (m, 3H), 3.61 (t, *J* = 6.0 Hz, 2H), 3.55 (t, *J* = 7.1 Hz, 2H), 3.21–3.03 (m, 4H), 1.86 –1.72 (m, 4H), 1.54–1.41 (m, 17H). ^13^C NMR (100 MHz, CDCl_3_) δ 170.2, 168.6, 165.2, 164.8, 156.2, 156.0, 79.3, 79.0, 52.4, 44.5, 43.9, 43.0, 37.8, 36.9, 28.5, 28.4, 27.9, 27.7. Maldi-tof m/z calcd for C_22_H_38_ClN_7_O_6_: 531.2, found 554.9 (M+Na)^+^.

#### 3.1.3. Methyl 2-((4-(bis(3-((*tert*-butoxycarbonyl)amino)propyl)amino)-6-((2-((2-oxo-2-phenylethylidene)amino)ethyl)amino)-1,3,5-triazin-2-yl)amino)acetate (**8**)

To a stirred solution of **7** (0.8 g, 1.5 mmol) and DIEA (1.38 mL, 7.5 mmol) in 1,4-dioxane (10 mL) was added benzyl (2-aminoethyl)carbamate (0.37 g, 5.84 mmol) slowly. The resultant solution was heated to reflux at 101 °C in an oil bath for 12 h, and then the reaction mixture was evaporated under vacuum. The resultant mixture was dissolved in H_2_O (15 mL) and EtOAc (15 mL) and extracted from EtOAc (2 × 10 mL). The combined organic layers were washed with brine (10 mL), dried over anhydrous Na_2_SO_4_, filtered, and evaporated. The residue was purified by flash chromatography on silica gel using CH_2_Cl_2_/EtOAc (1:1) to afford the title compound (**8**) (0.68 g, 68%) as a colorless oil. ^1^H NMR (400 MHz, MeOD) δ 7.44–7.11 (m, 5H), 5.06 (s, 2H), 4.05 (s, 2H), 3.80–3.60 (m, 4H), 3.64–3.38 (m, 7H), 3.13–2.90 (m, 4H), 1.82–1.61 (m, 4H), 1.43 (s, 18H). ^13^C NMR (100 MHz, MeOD) δ 172.0, 166.3, 164.7, 162.7, 157.6, 157.0, 136.9, 128.1, 127.6, 127.4, 78.6, 66.0, 51.1, 48.3, 48.1, 44.1, 42.3, 40.6, 40.0, 37.7, 27.8, 27.5. Maldi-tof *m*/*z* calcd for C_32_H_49_N_9_O_7_: 671.3, found 671.5.

#### 3.1.4. Methyl 2-((4-((2-((((9*H*-fluoren-9-yl)methoxy)carbonyl)amino)ethyl)amino)-6-(bis(3-((*tert*-butoxycarbonyl)amino)propyl)amino)-1,3,5-triazin-2-yl)amino)acetate (**9**)

To stirred solution of **8** (0.5 g, 0.745 mmol) in MeOH (10 mL), 10% palladium on carbon (50 mg) was added, and the resulting mixture was stirred under an atmosphere of H_2_ at room temperature for 5 h. The reaction mixture was filtered through celite, and the filtrate was concentrated under vacuum. The crude product was dissolved in CH_2_Cl_2_ (10 mL), and DIEA was added (0.64 µL, 3.46 mmol). To the resultant reaction mixture at 0 °C, Fmoc-OSu (0.3 g, 0.9 mmol) in CH_2_Cl_2_ (5 mL) added dropwise, and the temperature slowly increased to room temperature and stirred for 3 h. Then the reaction was quenched with the addition of H_2_O (15 mL), and the resultant mixture was extracted from CH_2_Cl_2_ (2 × 10 mL). The combined organic layers were washed with brine (20 mL), dried over anhydrous Na_2_SO_4_, filtered, and evaporated. The residue was purified by flash chromatography on silica gel using EtOAc/CH_2_Cl_2_ (1:1→2:1) to afford the title compound (**9**) (0.39 g, 69%) as a white solid. ^1^H NMR (400 MHz, CDCl_3_) δ 7.68 (d, *J* = 7.4 Hz, 2H), 7.51 (d, *J* = 6.8 Hz, 2H), 7.32 (t, *J* = 7.2 Hz, 2H), 7.27–7.17 (m, 2H), 4.42–4.21 (m, 2H), 4.19–4.09 (m, 1H), 4.10–3.97 (m, 2H), 3.67 (s, 3H), 3.58–3.21 (m, 7H), 3.15–2.85 (m, 4H), 2.63–2.05 (m, 4H), 1.79–1.52 (m, 4H), 1.37 (s, 16H). ^13^C NMR (100 MHz, CDCl_3_) δ 157.0, 156.0, 152.7, 143.9, 143.0, 141.3, 134.7, 129.0, 127.6, 127.1, 125.3, 125.0, 124.4, 119.9, 79.2, 66.8, 52.3, 47.2, 43.6, 42.7, 40.4, 37.7, 37.6, 28.5, 27.8. Maldi-tof m/z calcd for C_39_H_55_N_9_O_8_: 777.4, found 777.2.

#### 3.1.5. 2-((4-((2-((((9*H*-Fluoren-9-yl)methoxy)carbonyl)amino)ethyl)amino)-6-(bis(3-((*tert*-butoxycarbonyl)amino)propyl)amino)-1,3,5-triazin-2-yl)amino)acetic acid (**1**)

Me_3_SnOH (0.55 g, 3.05 mmol) was added to a solution of **9** (1.18 g, 1.52 mmol) in 1,2-dichloroethane (5 mL) and heated to 80 °C and stirred for 18 h. Additional amount of Me_3_SnOH (0.275 g, 1.52 mmol) was added at 3 and 7 h of the reaction. After the completion of the reaction, solvent was evaporated and the crude product was purified by flash chromatography on silica gel using CH_2_Cl_2_/MeOH (9:1→8:2) to afford the title compound (**1**) (1.0 g, 86%) as a white solid. ^1^H NMR (400 MHz, MeOD) δ 7.65 (d, *J* = 7.5 Hz, 2H), 7.48 (d, *J* = 7.4 Hz, 2H), 7.25 (t, *J* = 7.4 Hz, 2H), 7.15 (t, *J* = 7.4 Hz, 2H), 4.21 (d, *J* = 6.8 Hz, 2H), 4.04 (t, *J* = 6.8 Hz, 1H), 3.77 (s, 2H), 3.52–3.30 (m, 6H), 3.29–3.23 (m, 2H), 3.02–2.83 (m, 4H), 1.72–1.52 (m, 4H), 1.43–1.22 (m, 18H). ^13^C NMR (100 MHz, MeOD) δ 165.8, 163.1, 157.6, 157.0, 143.9, 141.2, 127.4, 126.6, 124.8, 119.5, 78.6, 66.4, 45.1, 44.0, 40.1, 37.6, 27.8, 27.4. Maldi-tof m/z calcd for C_38_H_53_N_9_O_8_: 763.4, found 763.2, 785.2 (M-H+Na)^+^.

#### 3.1.6. General Procedure A

##### *N,N*-Dibenzyl-4,6-dichloro-1,3,5-triazin-2-amine (**10a**)

To a stirred solution of 2,4,6-trichloro-1,3,5-triazine, **5** (0.50 g, 2.72 mmol) and DIEA (2.5 mL, 13.5 mmol) in CH_2_Cl_2_ (15 mL) at 0 °C, was added dibenzylamine (0.49 mL, 2.58 mmol) in CH_2_Cl_2_ (10 mL) dropwise over 30 min, ensuring that the temperature was maintained at 0 °C, and stirred further for 3 h at 0 °C. Then the reaction mixture was treated with water (10 mL) and extracted from CH_2_Cl_2_ (2 × 15 mL). The combined organic layer extracts were washed with brine (50 mL), and dried over Na_2_SO_4_ and evaporated. The resultant residue was purified by silica gel column chromatography (hexane/EtOAc, 9:1→8:2) to afford **10a** as a white solid (0.63 g, 67%). ^1^H NMR (400 MHz, CDCl_3_) δ 7.47–7.31 (m, 6H), 7.31–7.20 (m, 4H), 4.82 (s, 4H). ^13^C NMR (100 MHz, CDCl_3_) δ 170.5, 165.6, 135.4, 128.9, 128.1, 49.1. Maldi-tof *m*/*z* calcd for C_17_H_14_Cl_2_N_4_: 344.07, found 345.22 (M+H)^+^.

##### 4,6-Dichloro-*N*-(3,3-diphenylpropyl)-1,3,5-triazin-2-amine (**10b**)

The **10b** was synthesized according to the general procedure A, using 2,4,6-trichloro-1,3,5-triazine (0.50 g, 2.72 mmol), DIEA (2.5 mL, 13.5 mmol) and 3,3-diphenylpropylamine (0.54 g, 2.58 mmol). The crude residue was purified by silica gel column chromatography (hexane/EtOAc, 9:1) to afford **10b** as a white solid (0.59 g, 61%). ^1^H NMR (400 MHz, CDCl_3_) δ 7.41–7.13 (m, 10H), 6.56 (s, 1H), 4.01 (t, *J* = 7.9 Hz, 1H), 3.50 (q, *J* = 6.7 Hz, 2H), 2.42 (q, *J* = 7.6 Hz, 2H). ^13^C NMR (100 MHz, CDCl_3_) δ 170.9, 169.6, 165.8, 143.6, 128.8, 127.7, 126.7, 48.9, 40.4, 34.5. Maldi-tof m/z calcd for C_18_H_16_Cl_2_N_4_: 358.07, found 359.24 (M+H)^+^.

##### 4,6-Dichloro-*N*-(naphthalen-1-ylmethyl)-1,3,5-triazin-2-amine (**10c**)

The **10c** was synthesized according to the general procedure A, using 2,4,6-trichloro-1,3,5-triazine (1.5 g, 8.15 mmol), DIEA (7.5 mL, 13.5 mmol) and 1-naphthylmethylamine (1.27 g, 7.74 mmol). The crude residue was purified by silica gel column chromatography (hexane/EtOAc, 9:1→8:2) to afford **10c** as a white solid (1.85 g, 75%). ^1^H NMR (400 MHz, CDCl_3_) δ 7.92–7.82 (m, 2H), 7.80 (dd, *J* = 10.2, 2.9 Hz, 1H), 7.54–7.44 (m, 2H), 7.44–7.35 (m, 2H), 6.06 (s, 1H), 5.03 (d, *J* = 5.6 Hz, 2H). ^13^C NMR (100 MHz, CDCl_3_) δ 171.2, 170.1, 165.6, 133.9, 131.4, 131.1, 129.3, 129.1, 126.9, 126.8, 126.3, 125.5, 122.8, 43.7. Maldi-tof m/z calcd for C_14_H_10_Cl_2_N_4_: 304.02, found 305.7 (M+H)^+^.

#### 3.1.7. General Procedure B

##### Methyl 2-((4-chloro-6-(dibenzylamino)-1,3,5-triazin-2-yl)amino)acetate (**11a**)

To a stirred solution of **10a** (1.0 g, 2.90 mmol) and DIEA (2.67 mL, 14.5 mmol) in CH_2_Cl_2_ (20 mL) was added glycine methyl ester hydrochloride (0.54 g, 4.36 mmol) slowly. The resultant solution was stirred for 5 h at room temperature. After the completion of reaction, H_2_O (40 mL) was added. The resultant mixture was extracted from CH_2_Cl_2_ (2 × 20 mL). The combined organic layers were washed with brine (20 mL), dried over anhydrous Na_2_SO_4_, filtered, and evaporated. The residue was purified by flash chromatography on silica gel using hexane/EtOAc (7:3) to afford the title compound (**11a**) (0.983 g, 85%) as a white solid. ^1^H NMR (400 MHz, CDCl_3_) δ 7.25–7.14 (m, 6H), 7.11 (d, *J* = 7.0 Hz, 2H), 7.06 (t, *J* = 6.7 Hz, 2H), 6.23 (t, *J* = 5.1 Hz, 1H), 4.64 (s, 2H), 4.58 (s, 2H), 4.13–3.93 (m, 2H), 3.71–3.42 (m, 3H). ^13^C NMR (100 MHz, CDCl_3_) δ 170.3, 168.9, 165.7, 165.6, 137.0, 136.9, 128.7, 128.1, 127.7, 127.6, 127.5, 52.2, 48.7, 48.6, 42.9 (rotamer existence is present). Maldi-tof *m*/*z* calcd for C_20_H_20_ClN_5_O_2_: 397.1, found 398.8 (M+H)^+^.

##### Methyl 2-((4-chloro-6-((3,3-diphenylpropyl)amino)-1,3,5-triazin-2-yl)amino)acetate (**11b**)

The **11b** was synthesized according to the general procedure B, using **10b** (0.80 g, 2.23 mmol), DIEA (2.05 mL, 11.2 mmol) and glycine methyl ester hydrochloride (0.41 g 3.35 mmol). The crude residue was purified by silica gel column chromatography (hexane/EtOAc, 7.5:3.5) to afford **11b** (668 mg, 73%) as a white solid. ^1^H NMR (400 MHz, CDCl_3_) δ 7.18–7.06 (m, 8H), 7.03 (t, J = 7.0 Hz, 2H), 6.85 (t, *J* = 5.5 Hz, 1H), 6.46 (t, *J* = 6.0 Hz, 1H), 4.10–3.87 (m, 2H), 3.82 (t, *J* = 8.0 Hz, 1H), 3.70–3.44 (m, 3H), 3.20 (q, *J* = 8.0 Hz, 2H), 2.19 (q, *J* = 8.0 Hz, 2H). ^13^C NMR (100 MHz, CDCl_3_) δ 170.2, 168.0, 165.6, 165.5, 144.1, 128.6, 127.8, 127.7, 126.4, 52.3, 48.7, 42.7, 39.8, 34.8 (rotamer existence is present). Maldi-tof *m*/*z* calcd for C_21_H_22_ClN_5_O_2_: 411.1, found 412.8 (M+H)^+^, 433.9 (M+Na)^+^.

##### Methyl 2-((4-chloro-6-((naphthalen-1-ylmethyl)amino)-1,3,5-triazin-2-yl)amino)acetate (**11c**)

**11c** was synthesized according to the general procedure B, using **10c** (0.80 g, 2.62 mmol), DIEA (2.41 mL, 13.1 mmol) and glycine methyl ester hydrochloride (0.491 g, 3.93 mmol). The crude residue was purified by silica gel column chromatography (EtOAc/CH_2_Cl_2_, 3:1) to afford **11c** (650 mg, 70%) as a white solid. ^1^H NMR (400 MHz, MeOD) δ 8.07 (t, *J* = 8.5 Hz, 1H), 7.88 (d, *J* = 7.8 Hz, 1H), 7.79 (d, *J* = 7.8 Hz, 1H), 7.59 – 7.37 (m, 4H), 4.99 (s (br), 2H), 4.41 (s, 3H), 4.16–3.94 (m, 2H), 3.80–3.42 (m, 3H). ^13^C NMR (100 MHz, CDCl_3_) δ 170.0, 169.4, 166.1, 165.7, 134.1, 133.1, 132.6, 131.4, 129.1, 128.7, 126.8, 126.1, 125.6, 123.2, 52.4, 43.2, 43.0 (rotamer existence is present). Maldi-tof *m*/*z* calcd for C_17_H_16_ClN_5_O_2_: 357.0, found 358.9 (M+H)^+^, 380.9 (M+Na)^+^.

#### 3.1.8. General Procedure C

##### Methyl 2-((4-((2-((*tert*-butoxycarbonyl)amino)ethyl)amino)-6-(dibenzylamino)-1,3,5-triazin-2-yl)amino)acetate (**12a**)

*N*-Boc-ethylenediamine (0.4 g, 2.52 mmol) was added slowly to the solution of **11a** (0.5 g, 1.25 mmol) and DIEA (0.8 mL, 7.5 mmol) in 1,4-dioxane (10 mL). The resultant solution was heated to reflux at 101 ^o^C using an oil bath for 16 h, and then the reaction mixture was evaporated under vacuum. The resultant mixture was dissolved in H_2_O (15 mL) and EtOAc (15 mL) and extracted from EtOAc (2 × 10 mL). The combined organic layers were washed with brine (10 mL), dried over anhydrous Na_2_SO_4_, filtered, and evaporated. The residue was purified by flash chromatography on silica gel using hexane/EtOAc (1:1) to afford the title compound (**12a**) (0.4 g, 62%) as a white solid. ^1^H NMR (400 MHz, CDCl_3_) δ 7.41–7.16 (m, 10H), 6.36–5.00 (m (br), 2H), 4.89–4.66 (m, 4H), 4.11 (s, 1H), 3.89–3.55 (m, 3H), 3.55–3.38 (m, 2H), 3.39–3.07 (m, 2H), 2.62 (s (br), 1H), 1.43 (s, 9H). ^13^C NMR (100 MHz, CDCl_3_) δ 171.5, 168.4, 166.7, 165.8, 156.2, 138.4, 128.5, 127.8, 127.6, 127.3, 52.1, 48.5, 47.6, 42.8, 41.1, 40.7, 28.4. Maldi-tof m/z calcd for C_27_H_35_N_7_O_4_: 521.2, found 522.1 (M+H)^+^.

##### Methyl 2-((4-((2-((*tert*-butoxycarbonyl)amino)ethyl)amino)-6-((3,3-diphenylpropyl)amino)-1,3,5-triazin-2-yl)amino)acetate (**12b**)

The **12b** was synthesized according to the general procedure C, using **11b** (0.60 g, 1.45 mmol), *N*-Boc-ethylenediamine (0.47 g 2.91 mmol), and DIEA (1.3 mL, 7.29 mmol) in refluxing 1,4-dioxane. The crude residue was purified by silica gel column chromatography (CH_2_Cl_2_/EtOAc, 3:7→2:8) to afford **12b** (725 mg, 78%) as a white solid. ^1^H NMR (400 MHz, CDCl_3_) δ 7.35–7.23 (m, 8H), 7.19 (t, *J* = 6.8 Hz, 2H), 5.83–5.06 (m, 3H), 4.12 (s, 2H), 4.01 (t, *J* = 7.7 Hz, 1H), 3.85–3.58 (m, 3H), 3.55–3.15 (m, 6H), 2.72 (s, 1H), 2.44–2.21 (m, 2H), 1.44 (s, 9H). ^13^C NMR (100 MHz, CDCl_3_) δ 171.4, 167.5, 166.3, 165. 7, 156.2, 144.4, 128.5, 127.8, 126.3, 79.2, 52.2, 48.6, 42.6, 41.2, 40.6, 39.3, 35.5, 28.4. Maldi-tof *m*/*z* calcd for C_28_H_37_N_7_O_4_: 535.2, found 536.1 (M+H)^+^.

##### Methyl 2-((4-((2-((*tert*-butoxycarbonyl)amino)ethyl)amino)-6-((naphthalen-1-ylmethyl)amino)-1,3,5-triazin-2-yl)amino)acetate (**12c**)

The **12c** was synthesized according to the general procedure C, using **11c** (0.6 g, 1.68 mmol), *N*-Boc-ethylenediamine (0.53 g, 3.36 mmol), and DIEA (1.54 mL, 8.4 mmol) in refluxing 1,4-dioxane. The crude residue was purified by silica gel column chromatography (CH_2_Cl_2_/EtOAc, 3:7) to afford **12c** (557 mg, 69%) as a white solid. ^1^H NMR (400 MHz, MeOD) δ 8.23–8.05 (m, 1H), 7.89 (d, *J* = 7.8 Hz, 1H), 7.79 (d, *J* = 8.0 Hz, 1H), 7.62–7.38 (m, 4H), 5.00 (s, 2H), 4.06 (s, 2H), 3.82–3.64 (m, 2H), 3.56–3.36 (m, 3H), 3.28–3.06 (m, 2H), 1.42 (s, 9H). ^13^C NMR (100 MHz, MeOD) δ 172.0, 166.1, 165.7, 157.2, 134.9, 134.6, 133.9, 131.3, 128.3, 127.4, 125.8, 125.3, 125.1, 123.2, 78.7, 51.1, 41.8, 40.0, 27.4. Maldi-tof *m*/*z* calcd for C_24_H_31_N_7_O_4_: 481.2, found 482.0 (M+H)^+^.

#### 3.1.9. General Procedure D

##### Methyl 2-((4-((2-((((9*H*-fluoren-9-yl)methoxy)carbonyl)amino)ethyl)amino)-6-(dibenzylamino)-1,3,5-triazin-2-yl)amino)acetate (**13a**)

To a stirred solution of **12a** (1.0 g, 1.9 mmol) in CH_2_Cl_2_ (5 mL) at 0 °C, was added trifluoroacetic acid (TFA) (10 mL) dropwise, after the addition temperature increased to rt and stirred for 3 h. Then the reaction mixture was evaporated repeatedly using CH_2_Cl_2_ (3 × 10 mL) to remove the TFA. The crude reaction mixture was dissolved in CH_2_Cl_2_ (10 mL), to which DIEA (3.53 mL, 19 mmol) was added dropwise at 0 °C. Then, Fmoc-OSu (0.840 g, 2.49 mmol) in CH_2_Cl_2_ (10 mL) was added at 0 °C and stirred for 30 min. Then, the reaction temperature was increased slowly to room temperature and stirred for 3 h. The reaction was treated with H_2_O (15 mL), and the resultant mixture was extracted from CH_2_Cl_2_ (2 × 20 mL). The combined organic layers were washed with brine (20 mL), dried over anhydrous Na_2_SO_4_, filtered, and evaporated. The residue was purified by flash chromatography on silica gel using CH_2_Cl_2_/MeOH (9.5:0.5) to afford the title compound (**13a**) (740 g, 60%) as a white solid. ^1^H NMR (400 MHz, CDCl_3_) δ 7.77 (d, *J* = 7.5 Hz, 2H), 7.58 (d, *J* = 6.8 Hz, 2H), 7.40 (t, *J* = 7.4 Hz, 2H), 7.36–7.14 (m, 12H), 4.87–4.65 (m, 4H), 4.53–4.30 (m, 2H), 4.30–4.17 (m, 1H), 4.11 (s, 2H), 3.85–3.55 (m, 3H), 3.54–3.19 (m, 4H), 2.89 (s, 1H). ^13^C NMR (100 MHz, CDCl_3_) δ 171.2, 166.5, 165.4, 156.7, 154.9, 144.0, 141.3, 138.2, 128.5, 127.8, 127.7, 127.5, 127.1, 125.1, 119.9, 66.6, 52.1, 48.7, 47.9, 47.3, 42.7, 40.5. Maldi-tof *m*/*z* calcd for C_37_H_37_N_7_O_4_: 643.2, found 644.1 (M+H)^+^.

##### Methyl 2-((4-((2-((((9*H*-fluoren-9-yl)methoxy)carbonyl)amino)ethyl)amino)-6-((3,3-diphenylpropyl)amino)-1,3,5-triazin-2-yl)amino)acetate (**13b**)

The **13b** was synthesized using **12b** (3.2 g, 5.98 mmol) in CH_2_Cl_2_ (15 mL) and TFA (30 mL) at the Boc-deprotection stage. Further reaction was carried out using DIEA (11.05 mL, 60 mmol) and Fmoc-OSu (2.63 g, 7.8 mmol) in CH_2_Cl_2_ by following the general procedure D. The crude residue was purified by silica gel column chromatography CH_2_Cl_2_/MeOH (8.5:1.5) to afford **13b** (3.18 g, 81%) as a white solid. ^1^H NMR (400 MHz, CDCl_3_) δ 7.76 (t, *J* = 7.5 Hz, 2H), 7.57 (d, *J* = 7.1 Hz, 2H), 7.40 (t, *J* = 7.1 Hz, 2H), 7.36–7.11 (m, 12H), 6.13–5.10 (m, 3H), 4.54–4.30 (m, 2H), 4.20 (s, 1H), 4.11 (s, 2H), 3.98 (t, J = 7.6 Hz, 1H), 3.72 (s, 3H), 3.56–3.14 (m, 6H), 2.83 (s, 1H), 2.29 (q, *J* = 8.0 Hz, 2H). ^13^C NMR (100 MHz, CDCl_3_) δ 171.6, 169.3, 166.3, 165.9, 156.7, 144.4, 144.0, 141.3, 128.8, 128.5, 127.8, 127.7, 127.1, 126.3, 125.1, 121.0, 119.9, 119.7, 66.6, 52.2, 48.6, 47.3, 42.6, 41.9, 40.3, 39.3, 35.4. Maldi-tof m/z calcd for C_38_H_39_N_7_O_4_: 657.3, found 658.1 (M+H)^+^.

##### Methyl 2-((4-((2-((((9*H*-fluoren-9-yl)methoxy)carbonyl)amino)ethyl)amino)-6-((naphthalen-1-ylmethyl)amino)-1,3,5-triazin-2-yl)amino)acetate (**13c**)

The **13c** was synthesized using **12c** (1.26 g, 2.61 mmol) in CH_2_Cl_2_ (6 mL) and TFA (12 mL) at the Boc-deprotection stage. Further reaction was carried out using DIEA (3.64 mL, 26 mmol) and Fmoc-OSu (1.15 g, 3.4 mmol) in CH_2_Cl_2_ by following the general procedure D. The crude residue was purified by silica gel column chromatography CH_2_Cl_2_/EtOAc (2:8→1:9) to afford **13c** (1.13 g, 71%) as a white solid. ^1^H NMR (500 MHz, CDCl_3_) δ 8.12–7.95 (m, 1H), 7.93–7.82 (m, 1H), 7.76 (d, *J* = 7.4 Hz, 3H), 7.56 (d, *J* = 6.2 Hz, 2H), 7.52–7.44 (m, 2H), 7.44–7.33 (m, 4H), 7.29 (d, *J* = 8.5 Hz, 2H), 6.24–5.43 (m, 3H), 4.96 (s, 2H), 4.51–4.26 (m, 2H), 4.26–3.96 (m, 3H), 3.80–3.53 (m, 3H), 3.51–3.15 (m, 4H), 2.82 (s, 1H). ^13^C NMR (100 MHz, CDCl_3_) δ 171.5, 169.4, 165.8, 156.7, 144.0, 141.3, 134.2, 133.8, 131.4, 128.7, 128.2, 127.7, 127.1, 126.4, 125.8, 125.4, 125.1, 123.5, 119.9, 66.6, 52.2, 47.2, 42.7, 41.7, 40.4. Maldi-tof *m*/*z* calcd for C_34_H_33_N_7_O_4_: 603.2, found 604.0 (M+H)^+^.

#### 3.1.10. General Procedure E

##### 2-((4-((2-((((9*H*-Fluoren-9-yl)methoxy)carbonyl)amino)ethyl)amino)-6-(dibenzylamino)-1,3,5-triazin-2-yl)amino)acetic acid (**3a**)

The **13a** (1 g, 1.55 mmol) was dissolved in 10 mL of 1,4-dioxane and 10 mL of 2N HCL. The resultant mixture was heated to reflux at 101 °C for 4 h. Then the reaction mixture was evaporated to remove 1,4-dioxane, and the resultant residue was dissolved in H_2_O (15 mL) and EtOAc (15 mL), and extracted from EtOAc (2 × 15 mL). The combined organic layers were washed with brine (15 mL), dried over anhydrous Na_2_SO_4_, filtered, and evaporated under vacuum. The resultant sticky solid was treated with diethyl ether (20 mL) stirred for 20 min. The resultant solid was filtered and washed with diethyl ether (2 × 5 mL) to afford **3a** (932 mg, 95%) as a white solid. ^1^H NMR (400 MHz, CDCl_3_) δ 9.56 (s, 1H), 7.71 (d, *J* = 7.5 Hz, 2H), 7.59 (d, *J* = 7.2 Hz, 2H), 7.48–7.06 (m, 14H), 5.92 (s, 1H), 4.83 – 4.47 (m, 4H), 4.28 (d, *J* = 7.2 Hz, 2H), 4.19–3.97 (m, 3H), 3.58–3.13 (m, 4H). ^13^C NMR (100 MHz, CDCl_3_) δ 174.7, 163.4, 156.7, 156.5, 155.3, 144.0, 141.3, 136.8, 136.5, 128.7, 128.6, 127.9, 127.6, 127.5, 127.0, 125.2, 119.9, 66.7, 49.4, 49.3, 47.2, 44.8, 40.9, 40.1. Maldi-tof *m*/*z* calcd for C_36_H_35_N_7_O_4_: 629.1, found 629.1.

##### 2-((4-((2-((((9*H*-Fluoren-9-yl)methoxy)carbonyl)amino)ethyl)amino)-6-((3,3-diphenylpropyl)amino)-1,3,5-triazin-2-yl)amino)acetic acid (**3b**)

The **3b** was synthesized from **13b** (2.5 g, 3.8 mmol) in 25 mL of 1,4-dioxane and 25 mL of 2N HCL by following the general procedure E to afford **3b** (2.37 g, 95% ) as a white solid. ^1^H NMR (400 MHz, CDCl_3_) δ 9.14–8.50 (m, 1H), 7.97–7.65 (m, 2H), 7.66–7.46 (m, 2H), 7.48–6.94 (m, 14H), 6.63–5.75 (m, 2H), 5.55–5.10 (m, 1H), 4.42 (d, *J* = 6.1 Hz, 1H), 4.29 (d, *J* = 7.0 Hz, 1H), 4.17 (s, 2H), 4.09–3.78 (m, 2H), 3.61–2.98 (m, 6H), 2.42–2.07 (m, 2H). ^13^C NMR (100 MHz, CDCl_3_) δ 172.1, 157.4, 156.8, 154.3, 143.9, 143.8, 141.3, 141.1, 128.7, 128.6, 127.8, 127.7, 127.2, 126.6, 126.5, 126.9, 125.3, 119.9, 66.9, 48.6, 47.1, 42.5, 40.8, 40.1, 39.7, 34.8. Maldi-tof *m*/*z* calcd for C_37_H_37_N_7_O_4_: 643.2, found 643.1.

##### 2-((4-((2-((((9*H*-Fluoren-9-yl)methoxy)carbonyl)amino)ethyl)amino)-6-((naphthalen-1-ylmethyl)amino)-1,3,5-triazin-2-yl)amino)acetic acid (**3c**)

The **3c** was synthesized from **13c** (0.9 g, 1.49 mmol) in 10 mL of 1,4-dioxane and 10 mL of 2N HCL by following the general procedure E to afford **3c** (0.80 g, 91% ) as a white solid. ^1^H NMR (400 MHz, CDCl_3_) δ 8.95–8.29 (m (br), 1H), 8.07–6.97 (m, 15H), 6.75–5.94 (m (br), 2H), 4.79 (s, 2H), 4.39–3.84 (m, 5H), 3.58–2.78 (m, 4H). ^13^C NMR (100 MHz, DMSO) δ 171.4, 171.0, 156.7, 144.4, 141.2, 133.8, 131.4, 131.1, 129.1, 128.1, 127.5, 126.9, 126.4, 126.3, 125.9, 125.8, 125.6, 124.1, 123.8, 120.6, 65.9, 47.2, 42.9, 42.5, 42.3, 42.2, 41.9. Maldi-tof *m*/*z* calcd for C_37_H_37_N_7_O_4_: 643.2, found 643.1. Maldi-tof *m*/*z* calcd for C_33_H_31_N_7_O_4_: 589.2, found 589.0.

### 3.2. Peptide Synthesis

All the peptidomimetics and peptides were synthesized by SPPS using synthesized Fmoc-triazine amino acids and Rink amide Resin 100 mg with an initial loading of 0.61 mmol/g. Initially, the Resin was swollen in *N*,*N*-dimethylformamide (DMF) for 45 min before the synthesis. Then, the Fmoc group of the resin was deprotected by treating with 20% piperidine in DMF (1 × 10 min, 2 × 3 min). For the extension of sequence, initially, the Fmoc-triazine amino acid (5.0 equivalent) was activated using 2-(1H-benzotriazole-1-yl)-1,1,3,3-tetramethyluronium hexafluorophosphate (5.0 equivalent), 1-hydroxybenzotriazole (5.0 equivalent) and DIEA (10.0 equivalent) in DMF (2 mL) for 2 min. Then this solution was added to resin for coupling with the free amine, and the coupling reaction was proceeded for 1 h in a vortex stirring. The reaction mixture was filtered and washed with DMF, and then the Fmoc deprotection was achieved with 20% piperidine in DMF (1 × 10 min, 2 × 3 min). The resin was filtered and washed once again, and this process was repeated for the subsequent amino acids. After the sequence extension completed, Fmoc was deprotected by treating with 20% piperidine in DMF (1 × 10 min, 2 × 3 min). Then acetylation was performed at the free amine, using 20% acetic anhydride in DMF (1.5 mL) and 100 µL of DIEA. Finally, the resin was washed with DMF, MeOH, CH_2_Cl_2_, and ether and then dried under vacuum. The cleavage of peptidomimetics and peptide from the resin was carried out using 5% triisopropylsilane (TIS) and 5% H_2_O in trifluoroacetic acid (TFA, approximately 2 mL of TFA per 100 mg of resin) for 2 h. The cleavage mixture was filtered and mixed with cold diethyl ether. The precipitated peptide was purified on RP-HPLC using semi-preparative Vydac C18 column (20 mm × 250 mm, 15 μm,) and water/acetonitrile gradient in the presence of 0.05% TFA using a 280 nm detector. The pure fractions were collected and lyophilized. The purity of the peptidomimetics and peptides (>98%) was assessed by RP-HPLC on an analytical Vydac C18 column (4.6 mm × 250 mm, 300 Å, 5 μm particle size), using a 280 nm detector. These purified peptides mass were determined by MALDI-TOF MS (Shimadzu, Japan).

#### Peptidomimetics and Peptides

**BJK-1**, Maldi-tof *m*/*z* calcd for C_15_H_30_N_10_O_2_: 382.5, found 382.03 (7.8 mg, yield 34%, purity>95%).

**BJK-2**, Maldi-tof *m*/*z* calcd for C_23_H_28_N_8_O_2_: 448.5, found 448.9 (14.2 mg, yield 52%, purity>95%).

**BJK-3**, Maldi-tof *m*/*z* calcd for C_36_H_53_N_17_O_3_: 771.9, found 772.2 (27.4 mg, yield 58%, purity>95%).

**BJK-4**, Maldi-tof *m*/*z* calcd for C_49_H_78_N_26_O_4_: 1095.3, found 1094.9 (41.3 mg, yield 62%, purity>95%).

**BJK-5**, Maldi-tof *m*/*z* calcd for C_57_H_76_N_24_O_4_: 1161.3, found 1162.5 (41.1 mg, yield 58%, purity>95%).

**BJK-6**, Maldi-tof *m*/*z* calcd for C_70_H_101_N_33_O_5_: 1484.7, found 1486.1(48.6 mg, yield 54%, purity>95%).

**BJK-7**, Maldi-tof *m*/*z* calcd for C_19_H_27_N_5_O_3_: 373.4, found 373.8(12.5 mg, yield 57%, purity>95%).

**BJK-8**, Maldi-tof *m*/*z* calcd for C_25_H_39_N_7_O_4_: 501.6, found 502.0 (18.0 mg, yield 59%, purity>95%).

**BJK-9**, Maldi-tof *m*/*z* calcd for C_30_H_37_N_7_O_4_: 559.6, found 560.0 (20.7 mg, yield 61%, purity>95%).

**BJK-10**, Maldi-tof *m*/*z* calcd for C_36_H_4_9N_9_O_5_: 687.8, found 688.1 (26. 5mg, yield 63%, purity>95%).

### 3.3. Biology

#### 3.3.1. Materials

We purchased melittin and buforin-2 from AnyGen Co Ltd., South Korea. 3-(4,5-dimethylthiazol-2-yl)-2,5-diphenyl-2*H*-tetrazolium bromide (MTT), and diSC3-5 were purchased from Sigma-Aldrich (St. Louis, MO, USA). Invitrogen SYTOX green and *E.coli* bacterial plasmid pBR322 were purchased from Thermo Fisher Scientific, Seoul, Korea. Lonza Dulbecco’s Modified Eagle Medium (DMEM) and fetal bovine serum (FBS) were obtained from SeouLin Bioscience (Seoul, Korea). RAW 264.7 (mouse macrophage) was purchased from the American Type Culture Collection (Manassas, VA, USA). Buffers were prepared using Milli-Q ultrapure water (Merck Millipore, Billerica, MA, USA). All other reagents used were of analytical grade. Mueller Hinton broth (MHB) was purchased from Difco, USA. Gram-positive bacterial strains, including *Staphylococcus epidermidis* [KCTC 1917] and *Staphylococcus aureus* [KCTC 1621]), and Gram-negative bacterial strains, including *Escherichia coli* [KCTC 1682] and *Pseudomonas aeruginosa* [KCTC 1637] were procured from the Korean Collection for Type Cultures (KCTC) of the Korea Research Institute of Bioscience and Biotechnology (KRIBB), Seoul, Korea. All bacterial strains were made as glycerol stocks and stored at −80 °C, cultured on Luria Broth agar plates, and stored at 4 °C. Before the experiments, the single bacterial colony was chosen from the agar plates and cultured in MHB media overnight at 37 °C.

#### 3.3.2. Antimicrobial Activity

The bacterial cells were grown overnight for 18 h at 37 °C in 10 mL of Mueller Hinton broth (MHB). The 10 μL of this culture was inoculated into 10 mL of fresh MHB and incubated for an additional 3 h at 37 °C to obtain mid-logarithmic phase organisms. Aliquots (100 μL) of bacterial cell suspension at 4 × 10^6^ CFU/mL in 1% peptone were added to 100 μL of the sample solutions (serial 2-fold dilutions in 1% peptone). The final concentrations of the peptidomimetics, peptides, buforin-2, and melittin were ranged from 1 to 128 μg/mL. The MIC values were determined after 18 h of incubation. The MIC values were defined as the lowest concentration of the peptidomimetics or peptides at which no visible turbidity was observed comparing with the peptide-free control group.

#### 3.3.3. Hemolytic Activity

After centrifugation at 800× *g* for 10 min, fresh sheep red blood cells (sRBCs) were washed three times, with 10 mM phosphate buffer saline (PBS). RBCs were diluted to the final erythrocyte concentration of 8%. The RBC suspension (100 μL) was added to a 96-well microtiter plate. The peptidomimetics, peptides, and melittin dissolved in PBS were added to the wells of a 96-well plate by serial 2-fold dilution (100 μL/well). The final concentrations of these peptidomimetics and peptides were ranged from 1 to 256 μg/mL. PBS and 1% Triton X-100 were used as negative and positive control, respectively. The mixtures were incubated for 60 min at 37 °C, and then centrifuged at 1200× *g* for 15 min. The optical density (OD) values of the supernatants of the samples were measured with a microplate reader (Bio-Tek; EL 800) at 540 nm. Percent hemolysis was calculated as follows: % hemolysis = [OD_540(peptide)_ − OD_540(PBS)_]/[OD_540 (1%Triton X−100)_ − OD_540 (PBS)_], where OD_540(peptide)_ was OD value of the blood samples after incubation with peptide, OD_540 (1%Triton X-100)_ and OD_540(peptide)_ were the OD value of the blood sample treated by 1% Triton X-100 and PBS, respectively.

#### 3.3.4. Cytotoxicity Against RAW 264.7 Cells

To determine the cytotoxicity of **BJK-4**, we used the MTT (3-(4,5-dimethylthiazol-2-yl)-2,5-diphenyltetrazolium bromide) dye reduction assay against RAW 264.7 cells as previously described [34]. Briefly, the cells (2 × 10^4^ cells/well in DMEM supplemented with 10% FBS) were placed into 96-well plates and incubated for 18–24 h 5% CO_2_ at 37 °C. **BJK-4** was then added to the cells at final concentrations of 1–128 µg/mL. After incubation of 48 h, 20 µL MTT (5 mg/mL) reagent was added to each well and incubated for additional 4 h. The formazan crystals produced were dissolved in dimethyl sulfoxide (DMSO), and the absorbance at 570 nm was measured using a microplate ELISA reader.

#### 3.3.5. Protease Resistance

Digestion of the **BJK-4** and melittin by trypsin was carried out using 100 μg/mL peptide and 0.4 μg/mL trypsin in PBS, at 37 °C for 2 h. The reaction solution (50 μL) was added to 150 μL of a bacterial suspension (2 × 10^6^ CFU/mL in Mueller-Hinton broth). After incubation at 37 °C for 18 h, the bacterial growth inhibition was determined by measuring absorbance at 600 nm with a microplate ELISA reader (Bio-Tek; EL 800).

#### 3.3.6. Membrane Depolarization

The interaction of the **BJK-4**, melittin, and buforin-2 with the cytoplasmic membrane of bacteria cells was detected using the membrane potential-sensitive fluorescent dye 3,3′-dipropylthiadicarbocyanine iodide (DiSC_3_-5). Briefly, *S. aureus* KCTC 1621 was cultured to the mid-log phase at 37 °C and diluted to an OD_600_ of 0.08 in HEPES buffer (5 mM HEPES, 20 mM glucose, and 10 mM KCl, pH 7.4). The bacteria cells were incubated with 20 nM diSC_3_-5 until a stable reduction of the fluorescence was achieved. The cell suspension (3 mL) was placed in a 1-cm-path-length cuvette, and **BJK-4**, melittin, and buforin-2 (2 × MIC) were added. The fluorescence intensity changes were monitored using a Shimadzu RF-5300PC fluorescence spectrophotometer (Shimadzu Scientific Instruments, Kyoto, Japan) with an excitation wavelength of 622 nm and an emission wavelength of 670 nm. The membrane potential was entirely abolished by adding 1% Triton X-100.

#### 3.3.7. SYTOX Green Uptake

*S. aureus* KCTC 1621 was grown to mid-logarithmic phase at 37 °C, washed, and suspended (2 × 10^6^ CFU/mL) in HEPES buffer (20 mM glucose, 5 mM HEPES and 10 mM KCl, pH 7.4), after which SYTOX green (Molecular probes) was added to a final concentration of 1 mM, and the cells were incubated at 37 °C for 15 min with agitation in dark. After the addition of **BJK-4**, melittin and buforin-2 at the appropriate concentrations, the time-dependent increase in fluorescence caused by the binding of the cationic dye to intracellular DNA was monitored. After that, without **BJK-4**, melittin and buforin-2, and with **BJK-4**, melittin and buforin-2 (2 × MIC) were added, and then the increase in fluorescence was monitored using a Shimadzu RF-5300PC fluorescence spectrophotometer (Shimadzu Scientific Instruments, Kyoto, Japan) with an excitation wavelength of 485 nm and an emission wavelength of 520 nm. This is possible because the cationic dye, which can only cross compromise membranes, binds to intracellular DNA and this interaction results in a significant increase of fluorescence intensity.

#### 3.3.8. Flow Cytometry

The membrane damage by the **BJK-4**, melittin, and buforin-2 was determined by flow cytometry. In brief, *E. coli* KCTC 1682 was grown to mid-logarithmic phase in LB, washed thrice with PBS and diluted to with PBS to 2 × 10^7^ CFU/mL. The **BJK-4**, melittin, and buforin-2 were incubated with the bacterial suspension at a fixed PI concentration of 10 μg/mL for 1 h at 37 °C, followed by the removal of the unbound dye through washing with an excess of PBS. The data were recorded using a FACScan instrument (FACSCalibur, Beckman Coulter Inc., Indianapolis, IN, USA) with a laser excitation wavelength of 488 nm.

#### 3.3.9. Gel Retardation Assay

DNA retardation activity of **BJK-4** and buforin-2 was performed to examine the inhibition of the rate of migration of DNA bands through agarose gels. In brief, **BJK-4** and buforin-2 were mixed with a fixed concentration (100 ng) of plasmid DNA (pBR322) in a sample buffer (10 mM Tris-HCl, 5% glucose, 50 μg/mL BSA, 1 mM EDTA, and 20 mM KCl). The mixture of DNA, **BJK-4**, and buforin-2 samples were incubated at 37 °C for 1 h. After incubation, the samples were analyzed by 1% agarose gel electrophoresis in 0.5% TAE buffer. The plasmid bands were detected by UV illuminator (Bio-Rad, CA, USA).

## 4. Conclusions

To overcome the issues concerning the emergence of bacterial resistance and low proteolytic stability of AMPs, chemical modifications in the existing AMPs or development of new peptidomimetics are prescribed methods. Incorporation of unnatural amino acids in AMPs is also one such strategy to solve the issues pertaining to poor proteolytic stability of AMPs [16]. The present work describes the synthesis of cationic and hydrophobic Fmoc-triazine amino acids and their application in the construction of amphipathic antibacterial peptidomimetics. We identified the short peptidomimetic, **BJK-4** as the most potent antibacterial agent that showed no significant hemolytic activity. This antibacterial agent follows an intra-cellular targeting mechanism as buforin-2 against the bacteria. We also demonstrate that the peptide derived from Fmoc-triazine amino acids enhances proteolytic stability. Further derivatization of peptidomimetics and detailed antibacterial studies are under progress. Compared to peptoid antibacterials, our triazine peptidomimetics has advantages in terms of facile access to introducing various substrates by simple synthetic protocols. Moreover, synthesis of long peptidomimetics involving peptoids possesses demerits in terms of poor coupling yields in SPPS. In contrast, coupling yields of peptidomimetics involving triazine amino acids in SPPS are considerably good. In our previous study, we synthesized triazine polymer antibacterials using SPPS protocol, however to introduce the third substitution in triazine, we used elevated temperature and the yields were relatively low compared to the present study. In the present study, to synthesize peptidomimetics, we adopted the convenient and standard SPPS protocol with good yields [13]. Thus, this study may represent a model for the future development of antimicrobial peptidomimetics with high protease resistance.

## Figures and Tables

**Table 1:** Antimicrobial activity of the designed peptidomimetics, buforin-2 and melittin.

Peptidomimetics and Peptides	MIC ^a^ (μg/mL)				Net Charge	R^t^ (min) ^b^
	Gram-Negative Bacteria		Gram-Positive Bacteria			
	*E. coli* (KCTC 1682)	*P. aeruginosa* (KCTC 1637)	*Staphylococcus aureus* (KCTC 1621)	*Staphylococcus epidermidis* (KCTC 1917)		
BJK-1	128	128	>128	>128	+2	8.8
BJK-2	>128	>128	>128	>128	0	21.8
BJK-3	64	32	32	32	+2	18.3
BJK-4	16	16	16	16	+4	16.2
BJK-5	128	32	64	64	+2	21.1
BJK-6	16	16	16	16	+4	19.1
BJK-7	128	128	>128	>128	+1	15.4
BJK-8	64	128	>128	>128	+2	14.2
BJK-9	>128	>128	>128	>128	+1	19.6
BJK-10	>128	>128	>128	>128	+2	17.9
melittin	16	16	16	16		
buforin-2	32	128	32	64		

^a^ MICs (minimal inhibitory concentrations) were determined as the lowest concentration of the peptidomimetics or peptides that cause 100% inhibition of microbial growth. ^b^ HPLC retention time in minutes, using method A as stated in the Supplementary Material. KCTC = Korean Collection for Type Cultures.

## Supplementary Materials

Supplementary materials can be found at https://www.mdpi.com/1422-0067/21/10/3602/s1.
