# Supplementary material for: Synthesis of Fmoc-Triazine Amino Acids and Its Application in the Synthesis of Short Antibacterial Peptidomimetics
Source: Int J Mol Sci. 2020 May 20;21(10):3602. doi: 10.3390/ijms21103602 (PMC7279249; doi:10.3390/ijms21103602)
Supplement: Supplementary file 1 [file ijms-21-03602-s001.pdf]

Synthesis of Fmoc-Triazine Amino Acids and Its Application in the Synthesis of Short Antibacterial Peptidomimetics

Pethaiah Gunasekaran [1,†], Eun Young Kim [2,], Jian Lee [1], Eun Kyoung Ryu [1,4,†], Song Yub Shin [2,3] and Jeong Kyu Bang [1,4,*]

[1] Division of Magnetic Resonance, Korea Basic Science Institute (KBSI), Ochang 28119, Korea; gunaharaks@kbsi.re.kr (P.G.); paperin@kbsi.re.kr (J.L.); ekryu@kbsi.re.kr (E.K.R.)
[2] Department of Medical Science, Graduate School, Chosun University, Gwangju 61452, Korea; lovetive@naver.com (E.Y.K.); syshin@chosun.ac.kr (S.Y.S.)
[3] Department of Cellular and Molecular Medicine, School of Medicine, Chosun University, Gwangju 61452, Korea
[4] Department of Bio-analytical Science, University of Science & Technology, Daejeon 34113, Korea
* Correspondence: bangjk@kbsi.re.kr; Tel.: +82-43-240-5023
† These authors contributed equally to this work.

No	Contents	Page No
1	HPLC chromatogram of BJK-(1-10)	S3-S15
2	^{1}H and ^{13}C NMR spectra	S16-S55

HPLC

Method A: YL9100, C_{18} column (4.6 × 250 mm, 10 microns) with eluent A (0.05% of TFA in H_2O) and eluent B (0.05% of TFA in acetonitrile). Gradient, 5% of eluent B to 95% of Eluent B in 30 min at 25 ℃; flow rate1.5 mL/min; UV 280 nm;

Method B: YL9100, C_{18} column (4.6 × 250 mm, 10 microns) with eluent A (0.05% of TFA in H_2O) and eluent B (0.05% of TFA in acetonitrile). Gradient, 0% of Eluent B to 20% of Eluent B in 40 min at 25 ℃; flow rate,1.5 mL/min; UV, 280 nm.

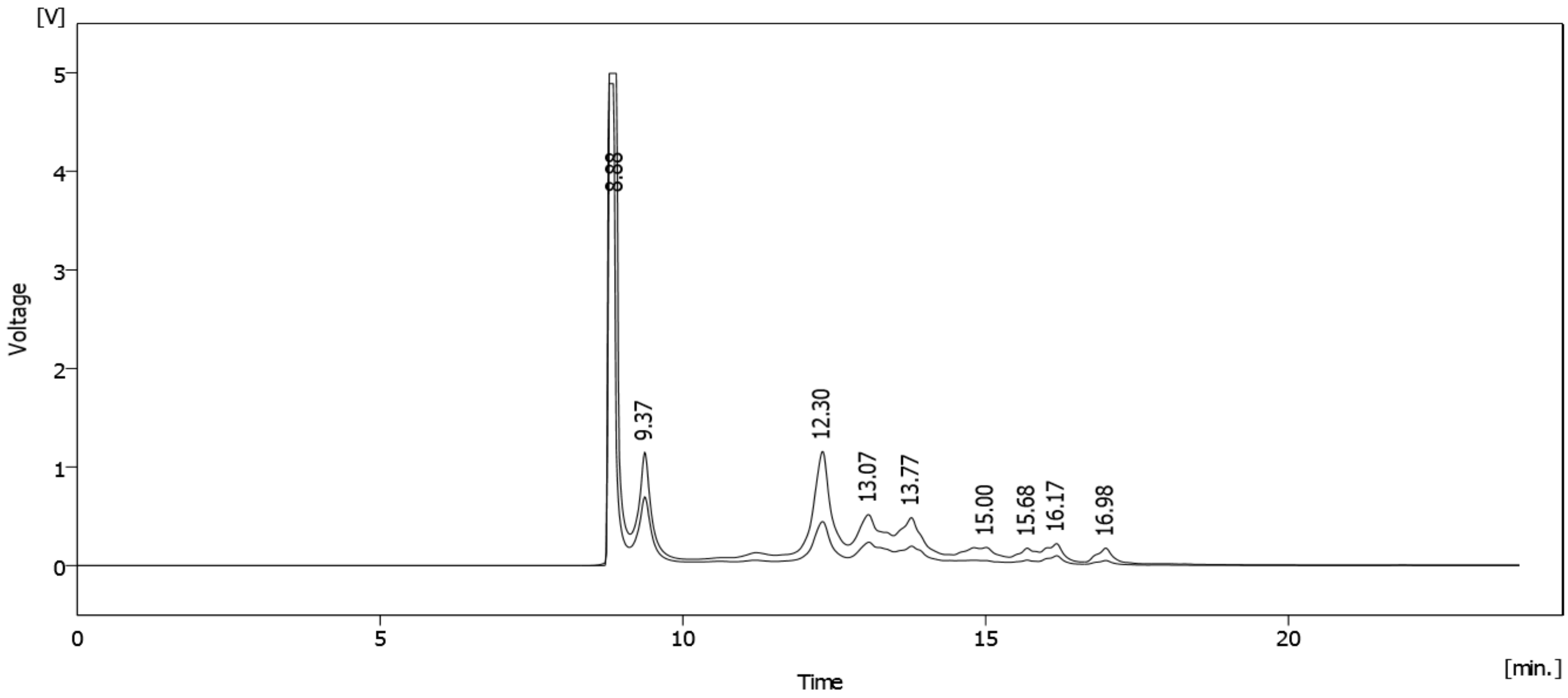

Crude HPLC of **BJK-1**, product RT=8.8 min (Method A)

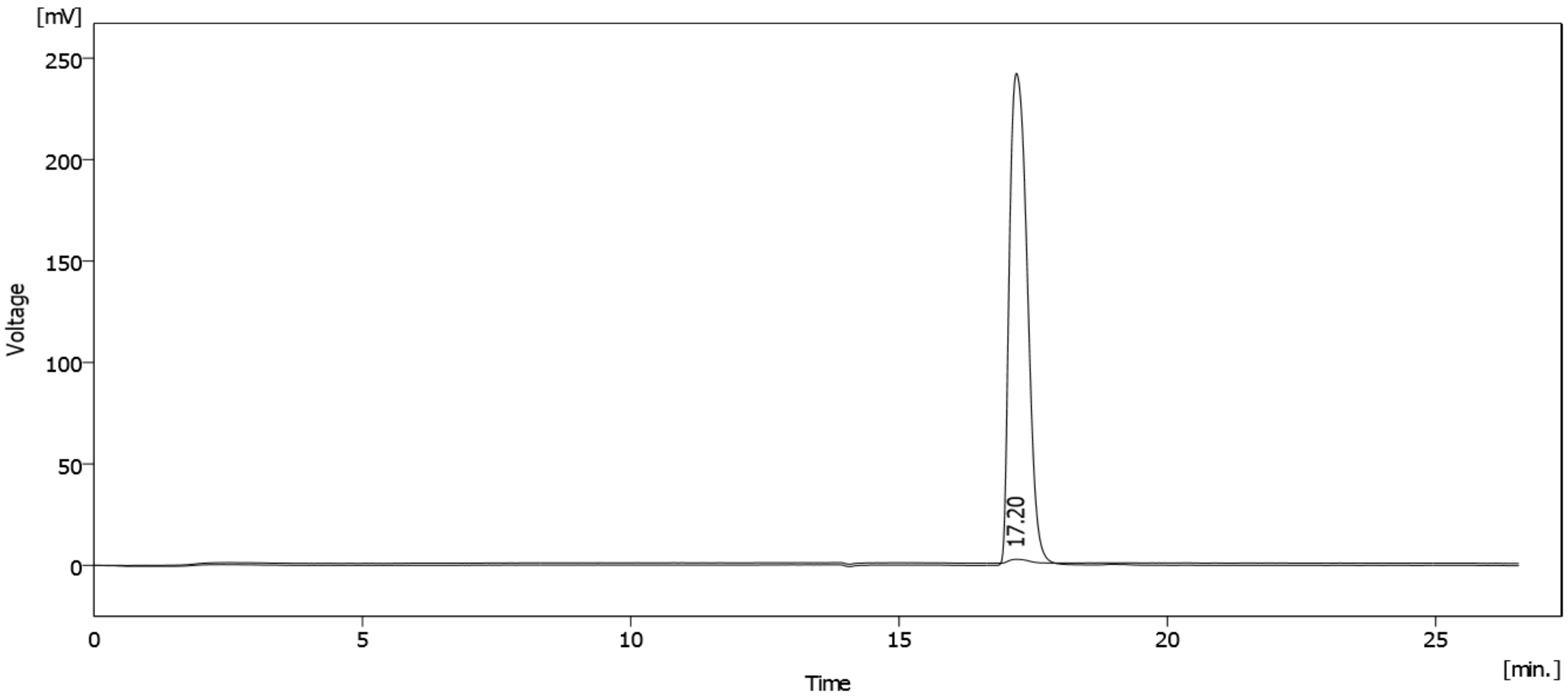

HPLC of **BJK-1** following Method B

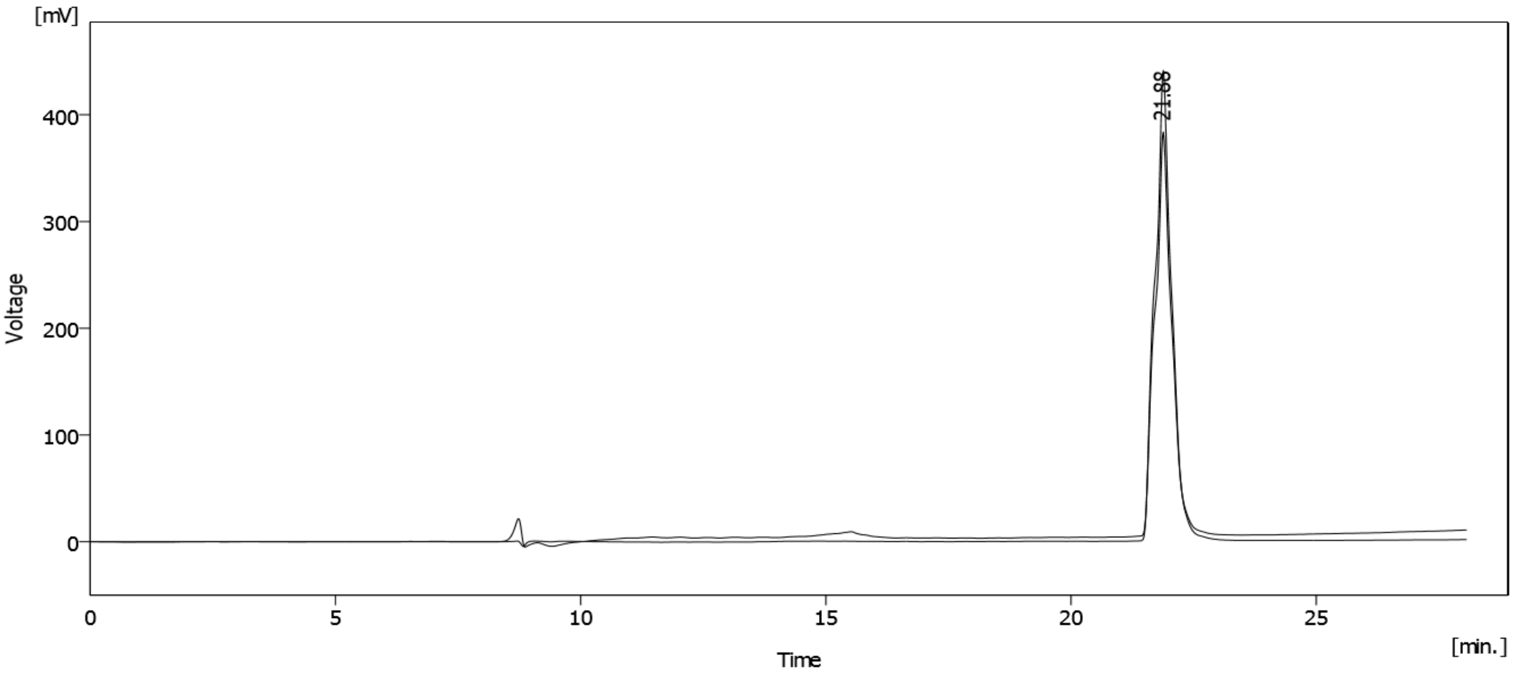

HPLC of **BJK-2** following Method A

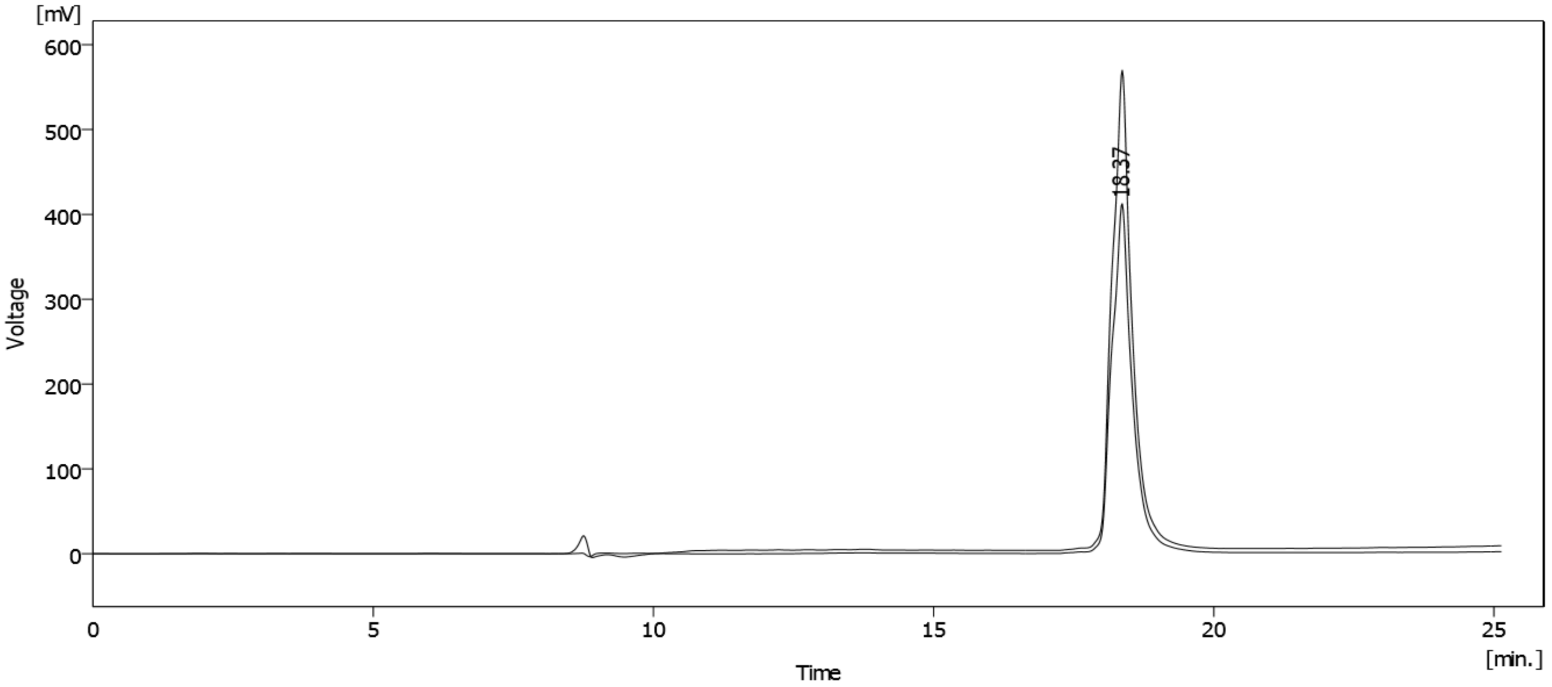

HPLC of **BJK-3** following Method A

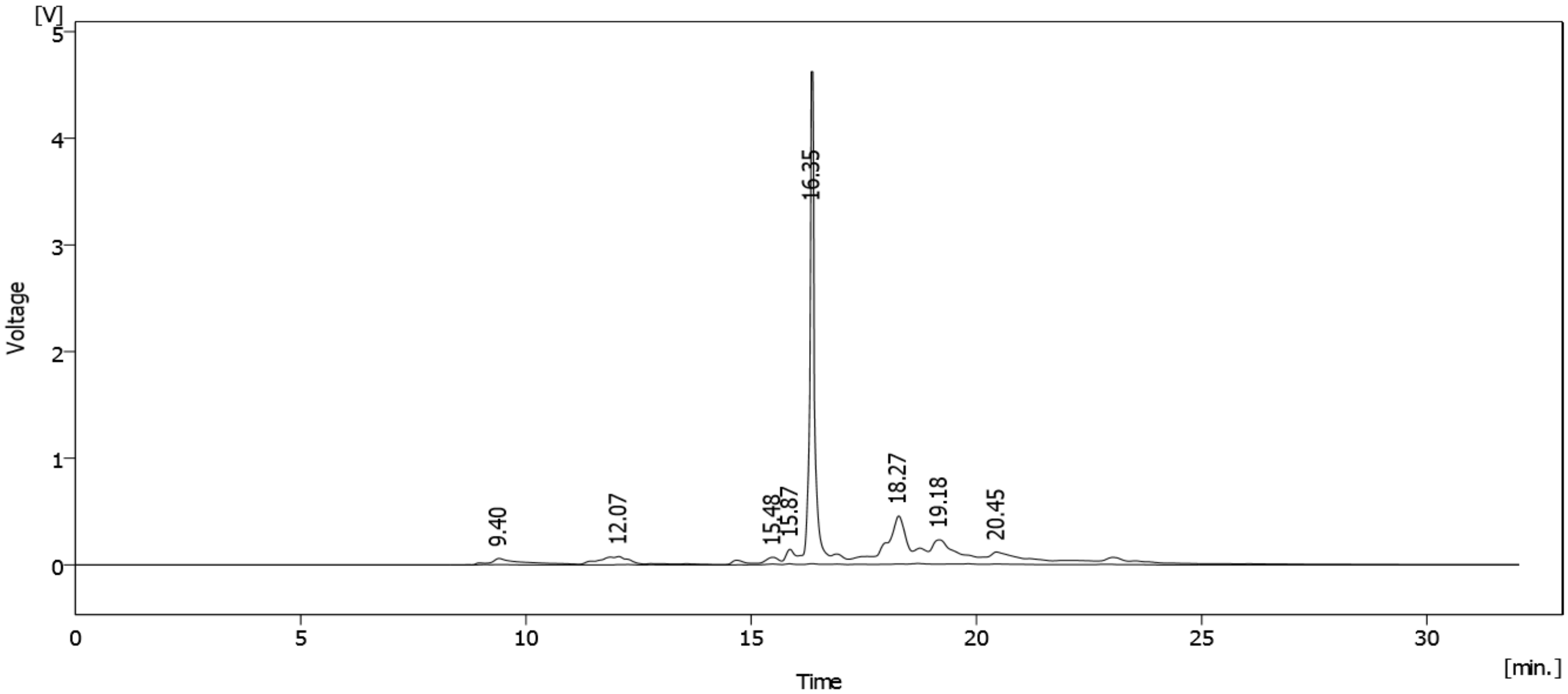

Representative crude HPLC-chromatogram of **BJK-4** following Method A

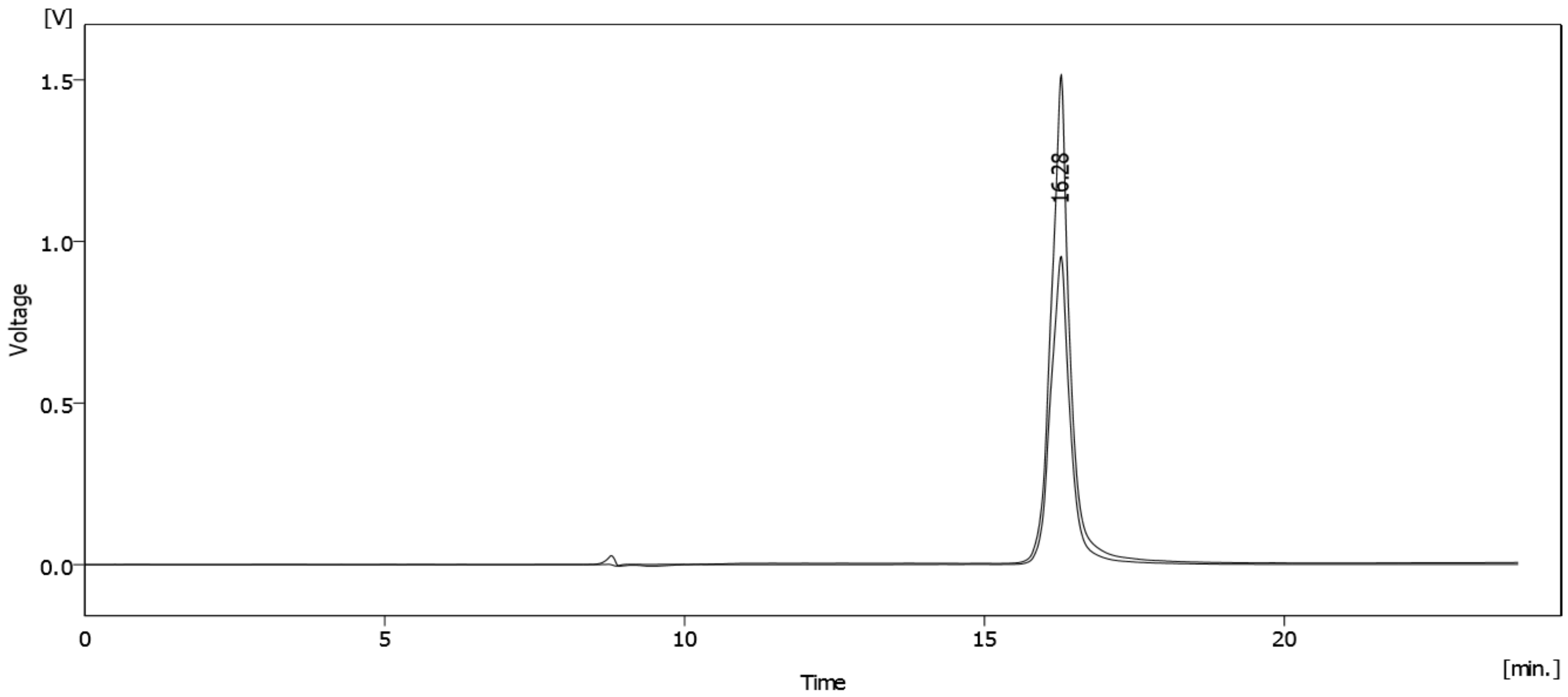

HPLC of **BJK-4** following Method A

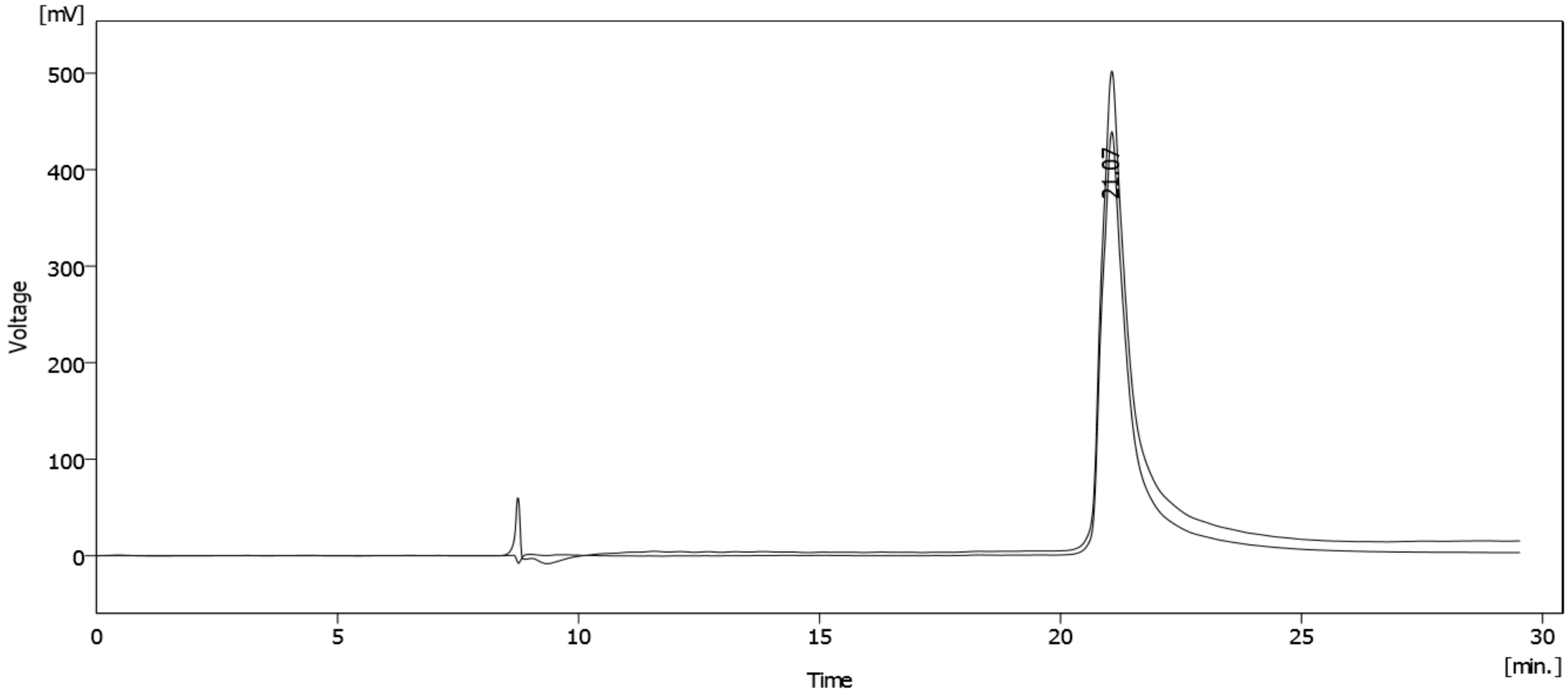

HPLC of **BJK-5** following Method A

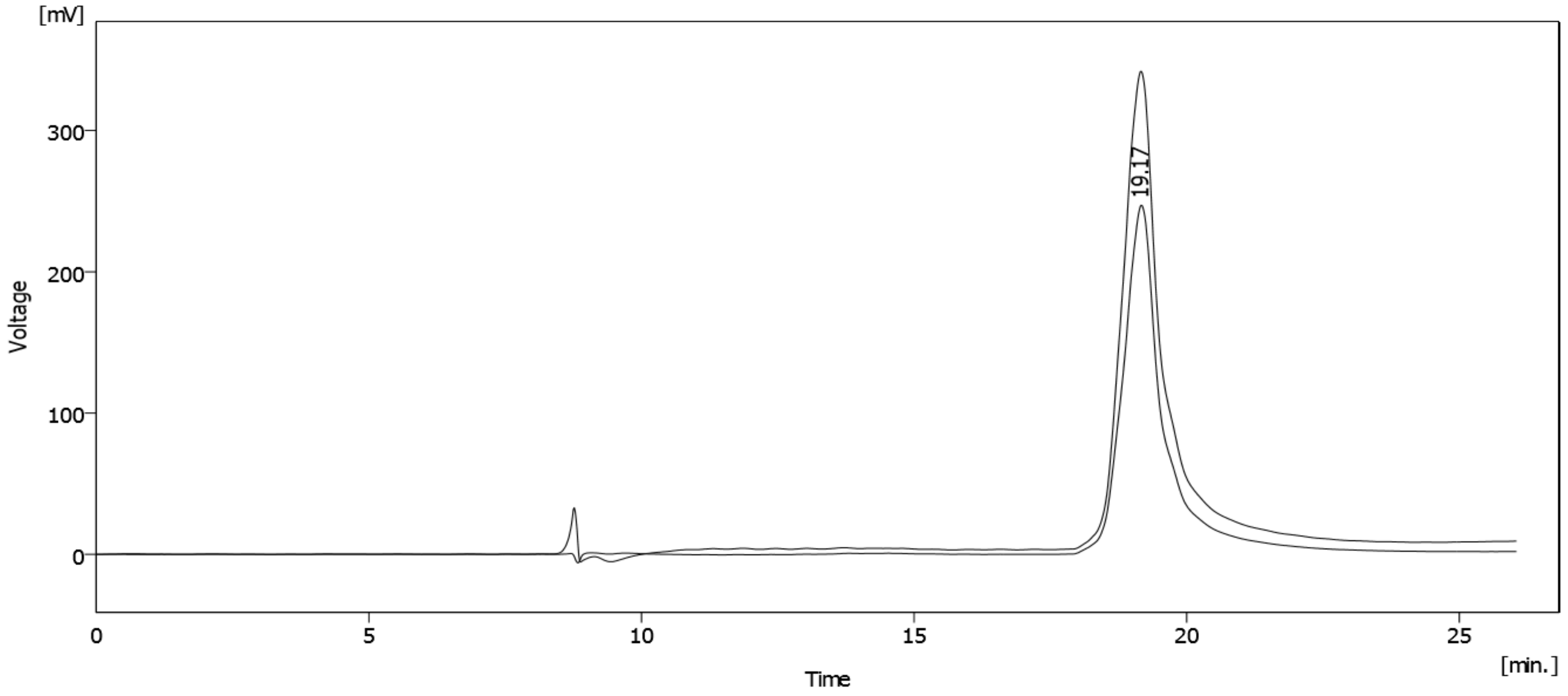

HPLC of **BJK-6** following Method A

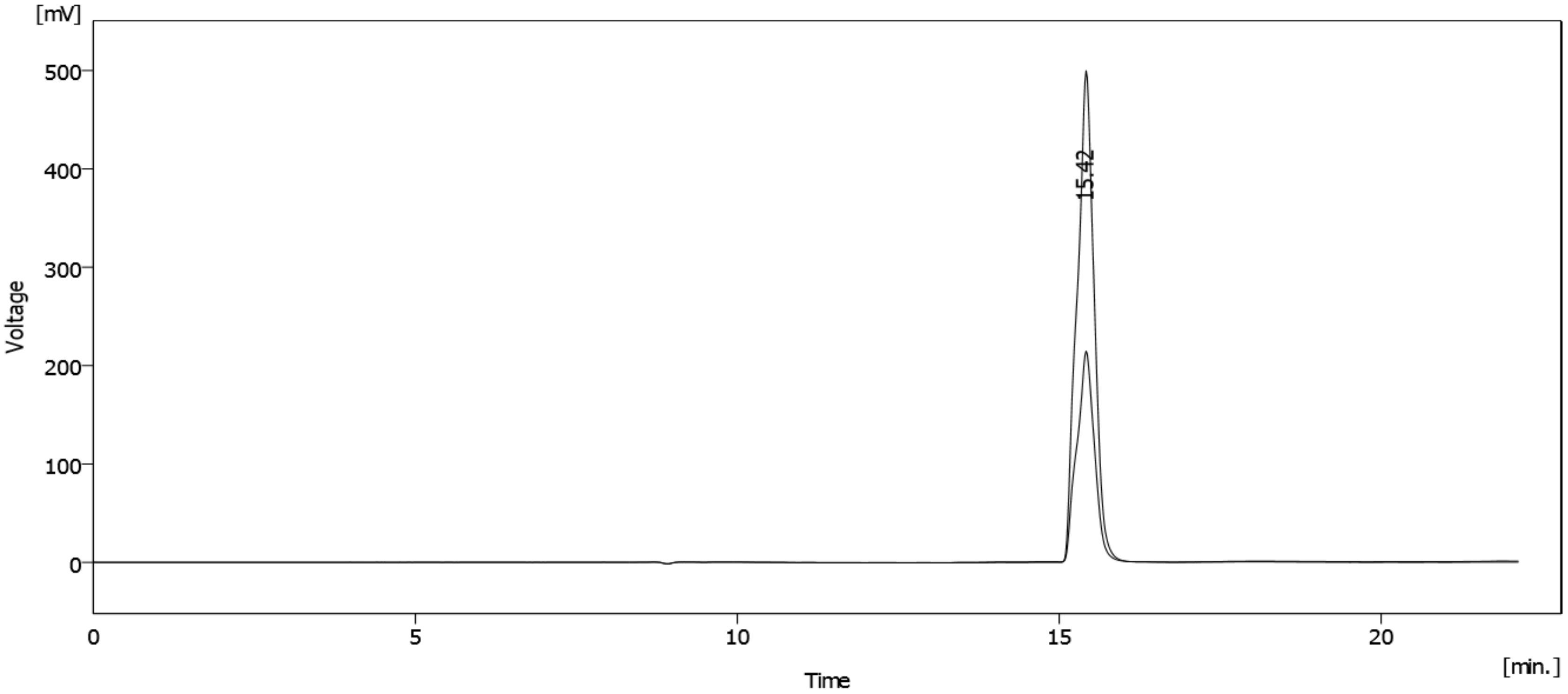

HPLC of **BJK-7** following Method A

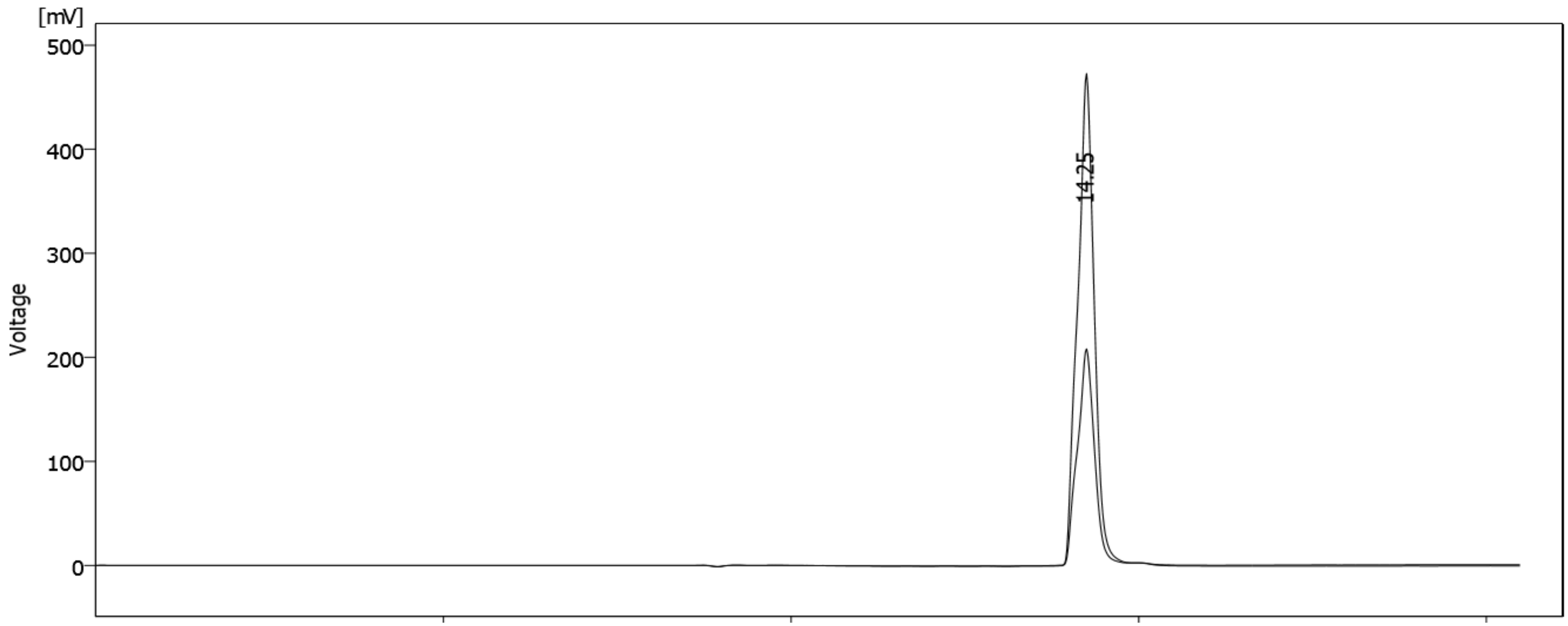

HPLC of **BJK-8** following Method A

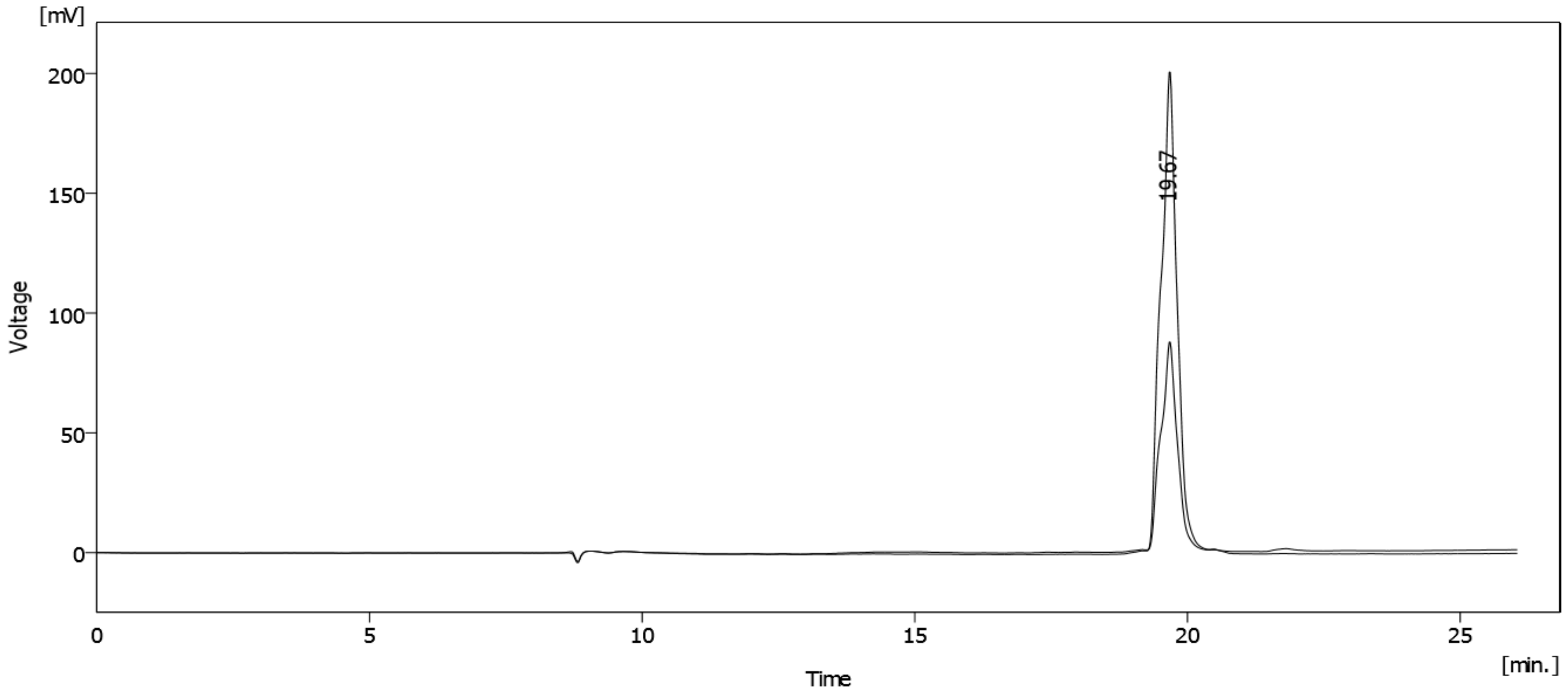

HPLC of **BJK-9** following Method A

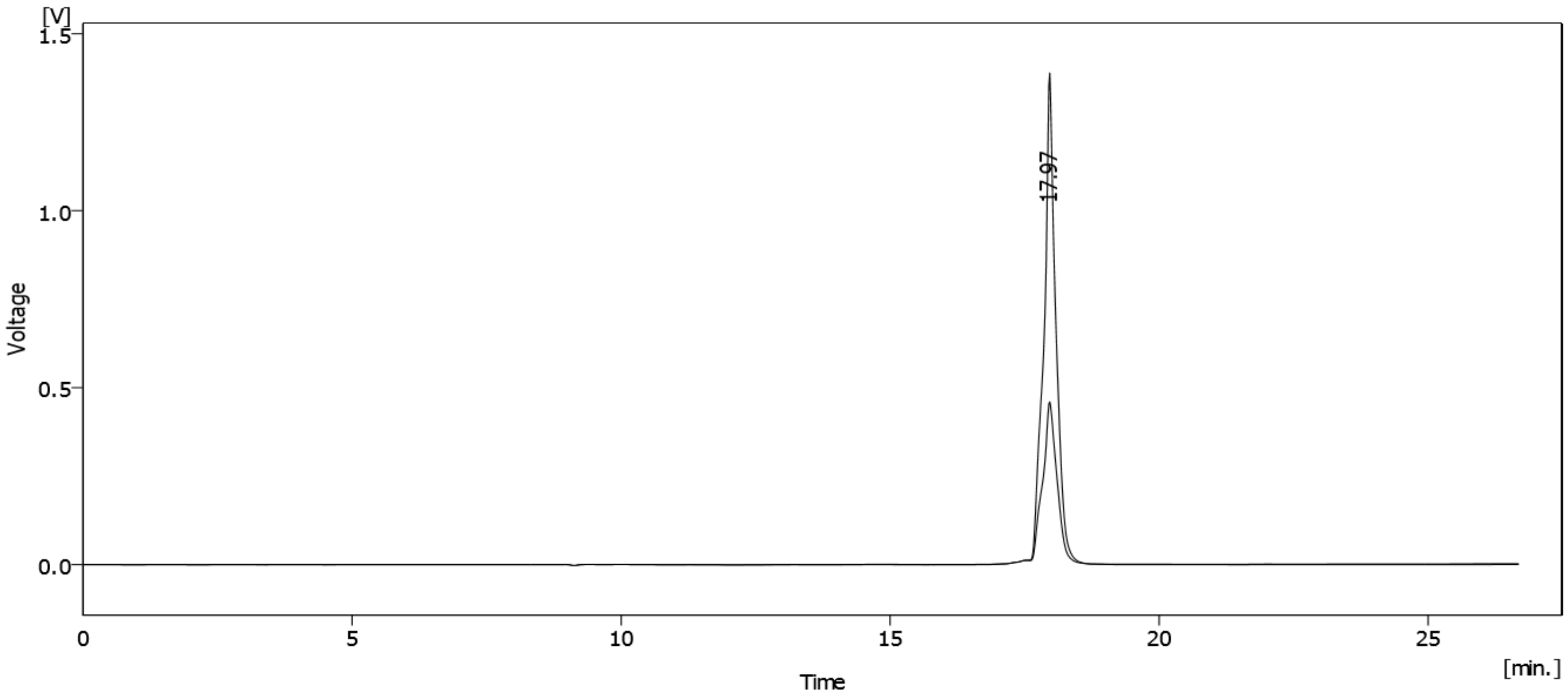

HPLC of **BJK-10** following Method A

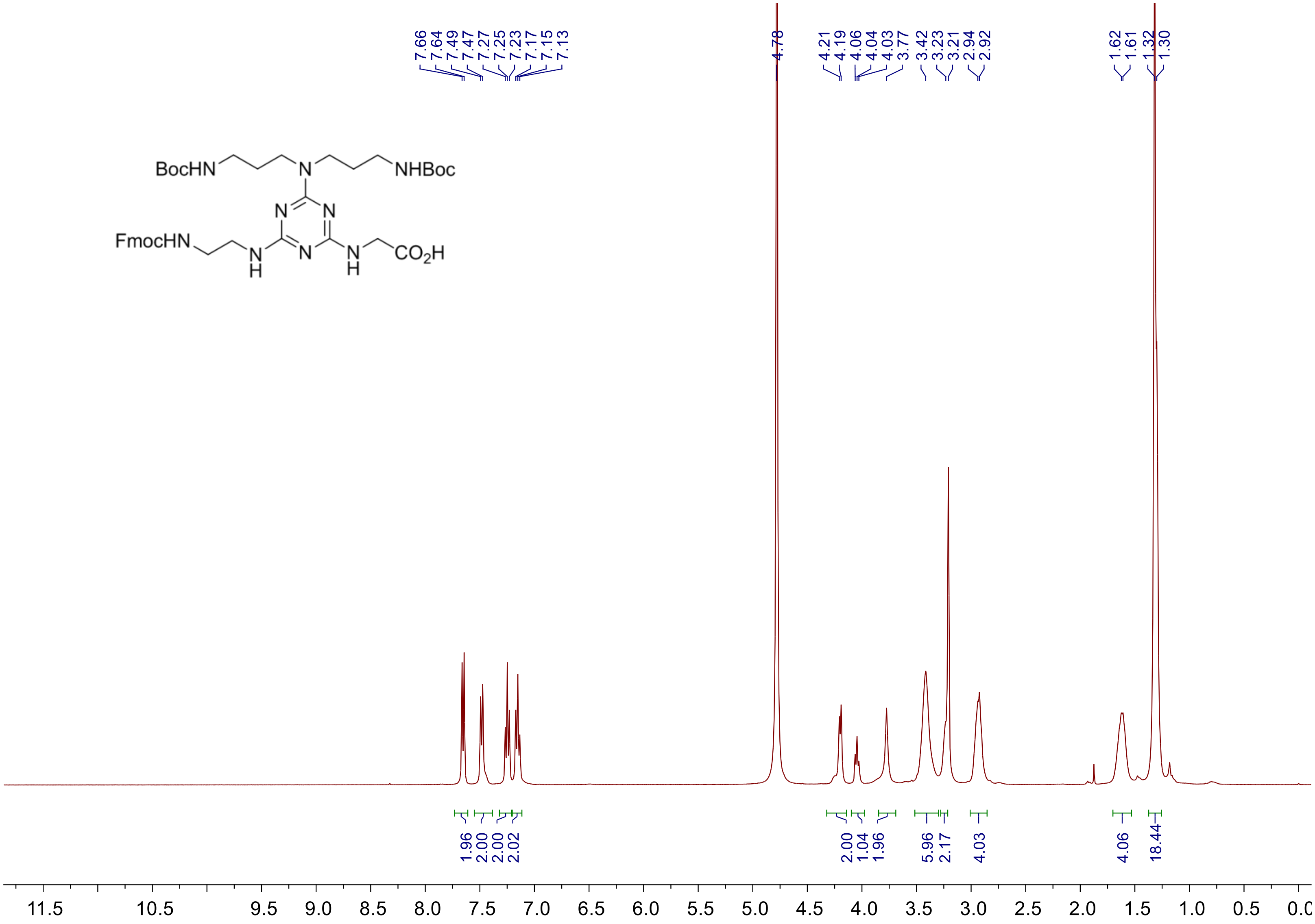

^{1}H NMR spectrum of **1** (MeOD, 400 MHz)

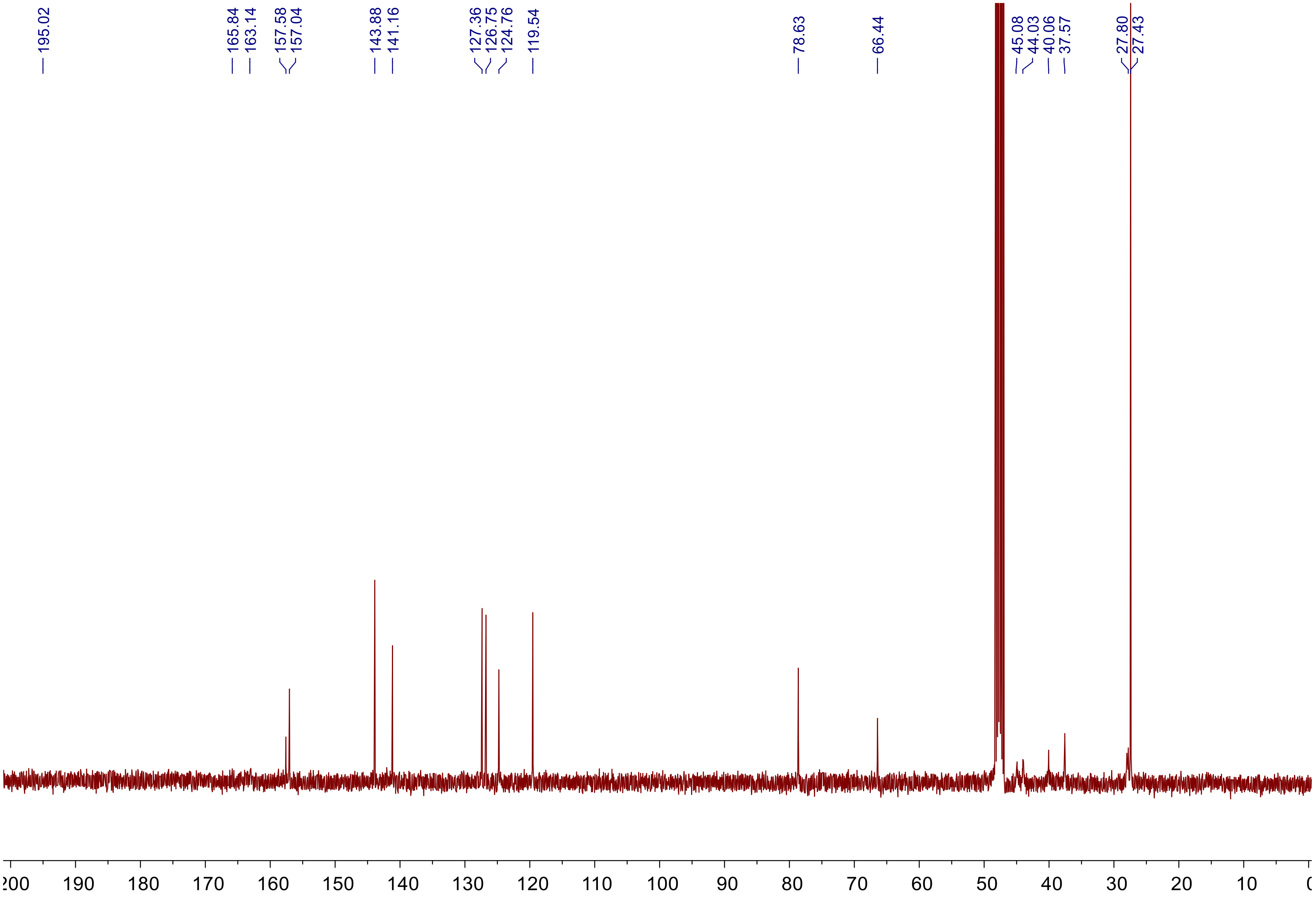

^{13}C NMR spectrum of **1** (MeOD, 400 MHz)

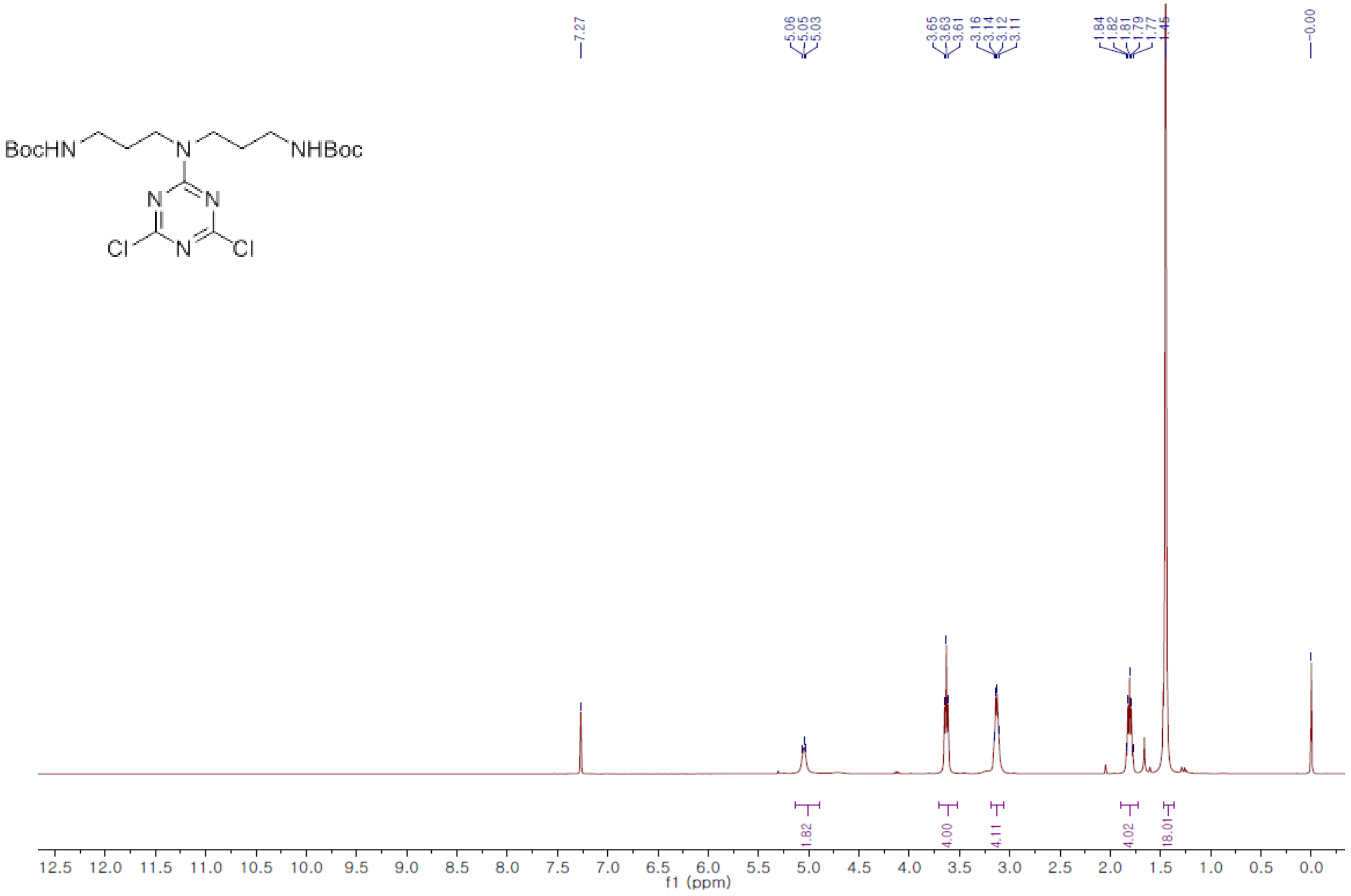

^{1}H NMR spectrum of **6** ($CDCl_3$, 400 MHz)

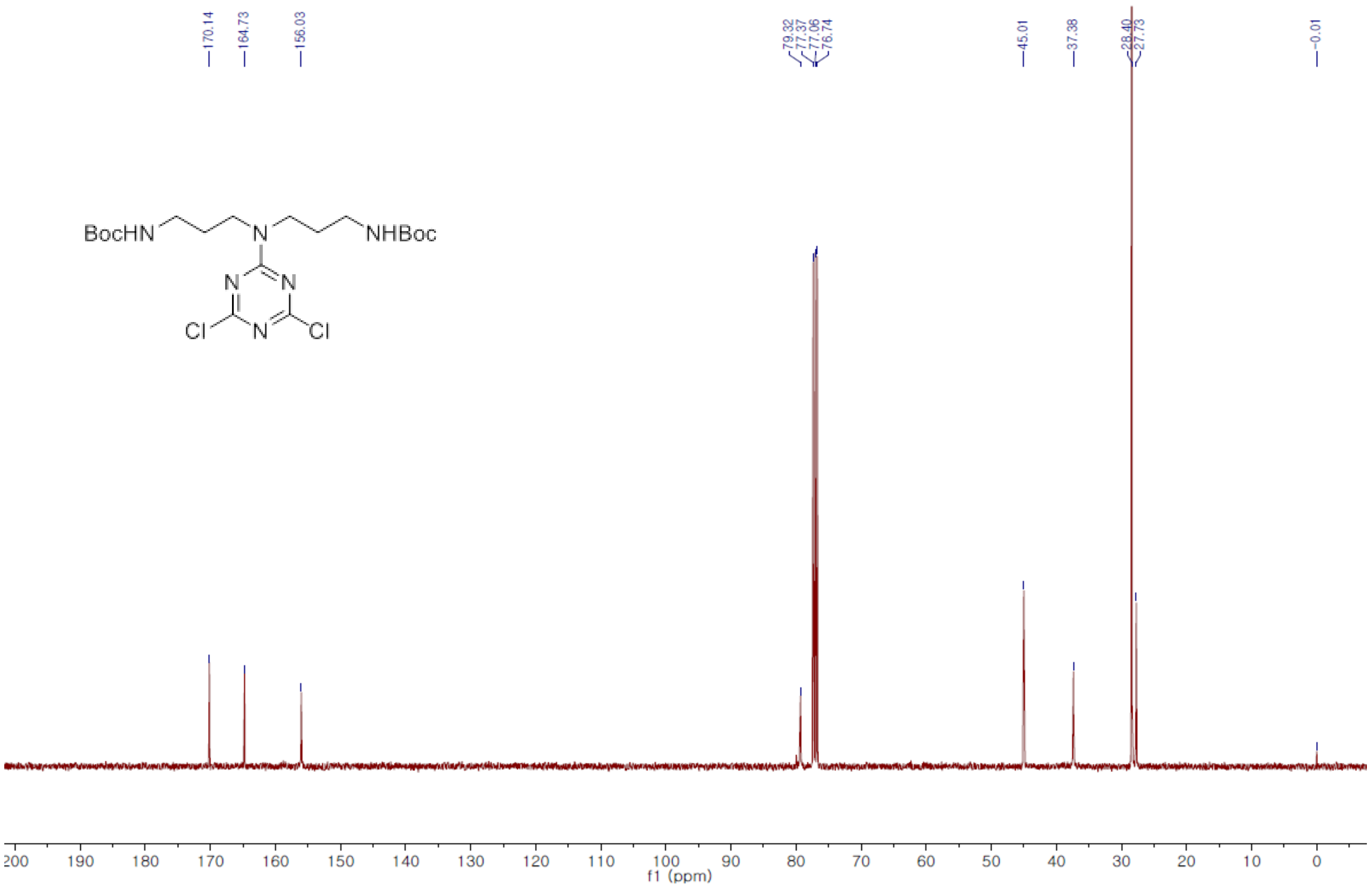

^{13}C NMR spectrum of **6** ($CDCl_3$, 100 MHz)

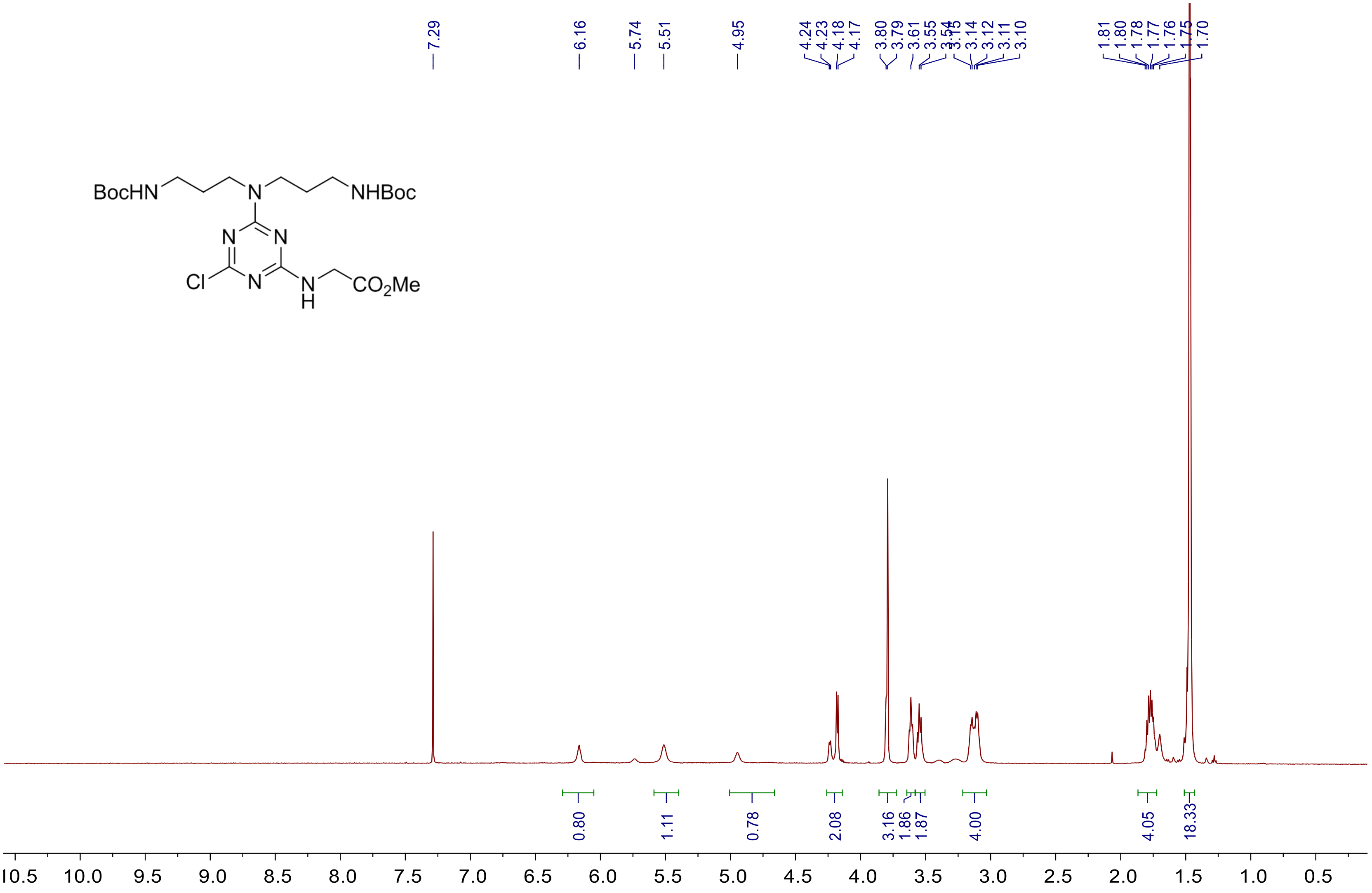

^1H NMR spectrum of **7** ($CDCl_3$, 400 MHz)

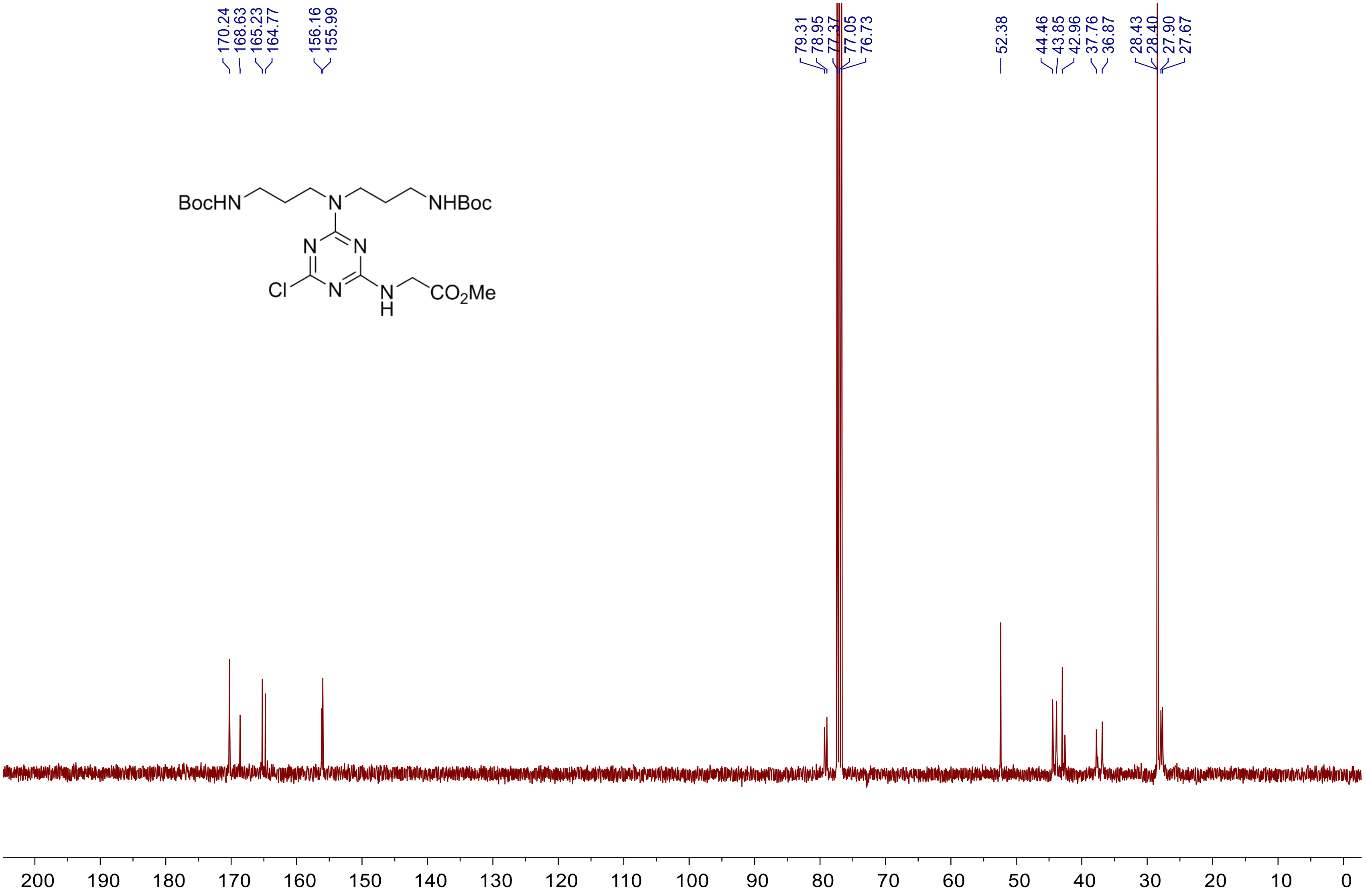

^{13}C NMR spectrum of **7** ($CDCl_3$, 100 MHz)

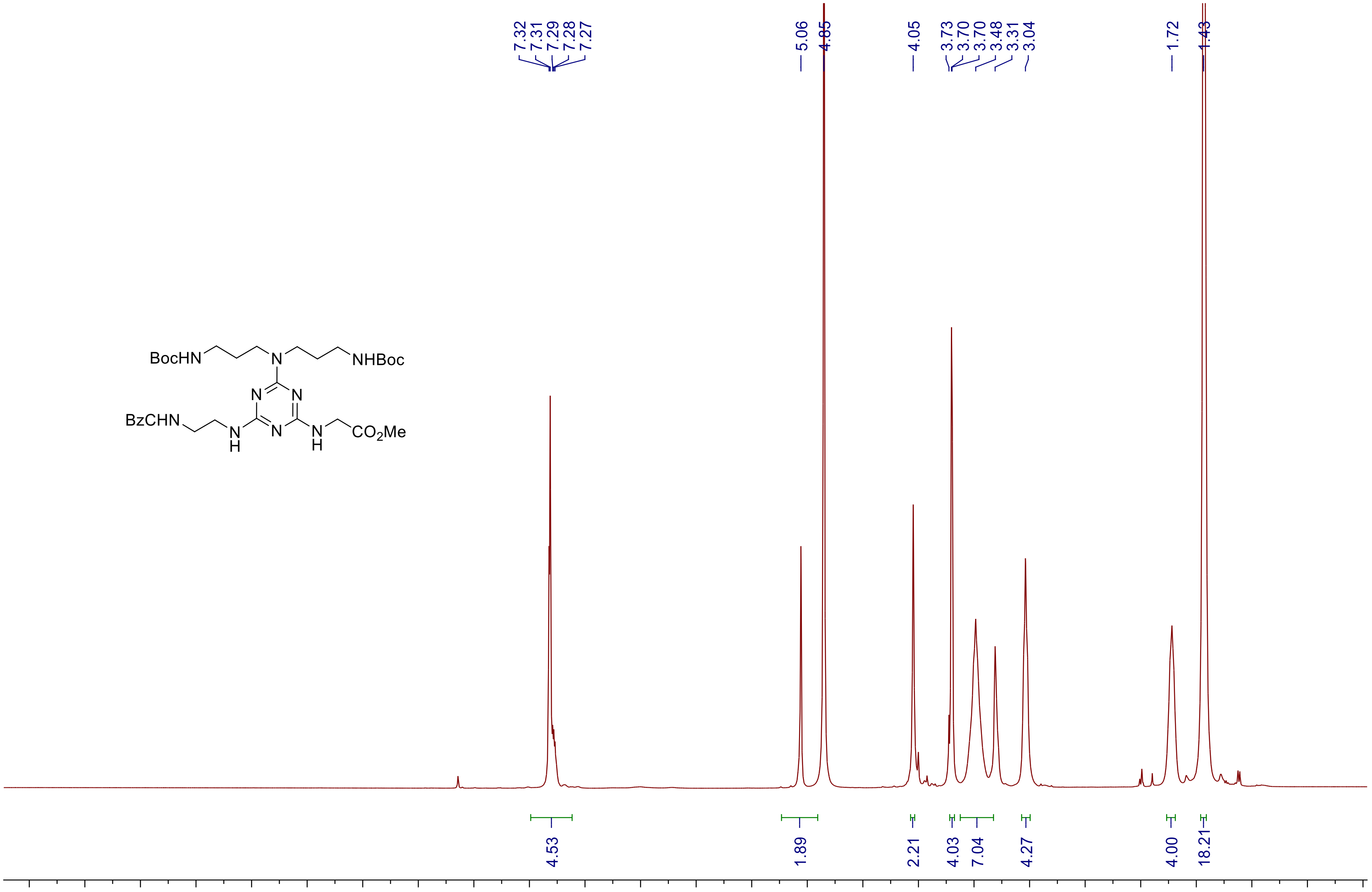

^{1}H NMR spectrum of **8** (MeOD, 400 MHz)

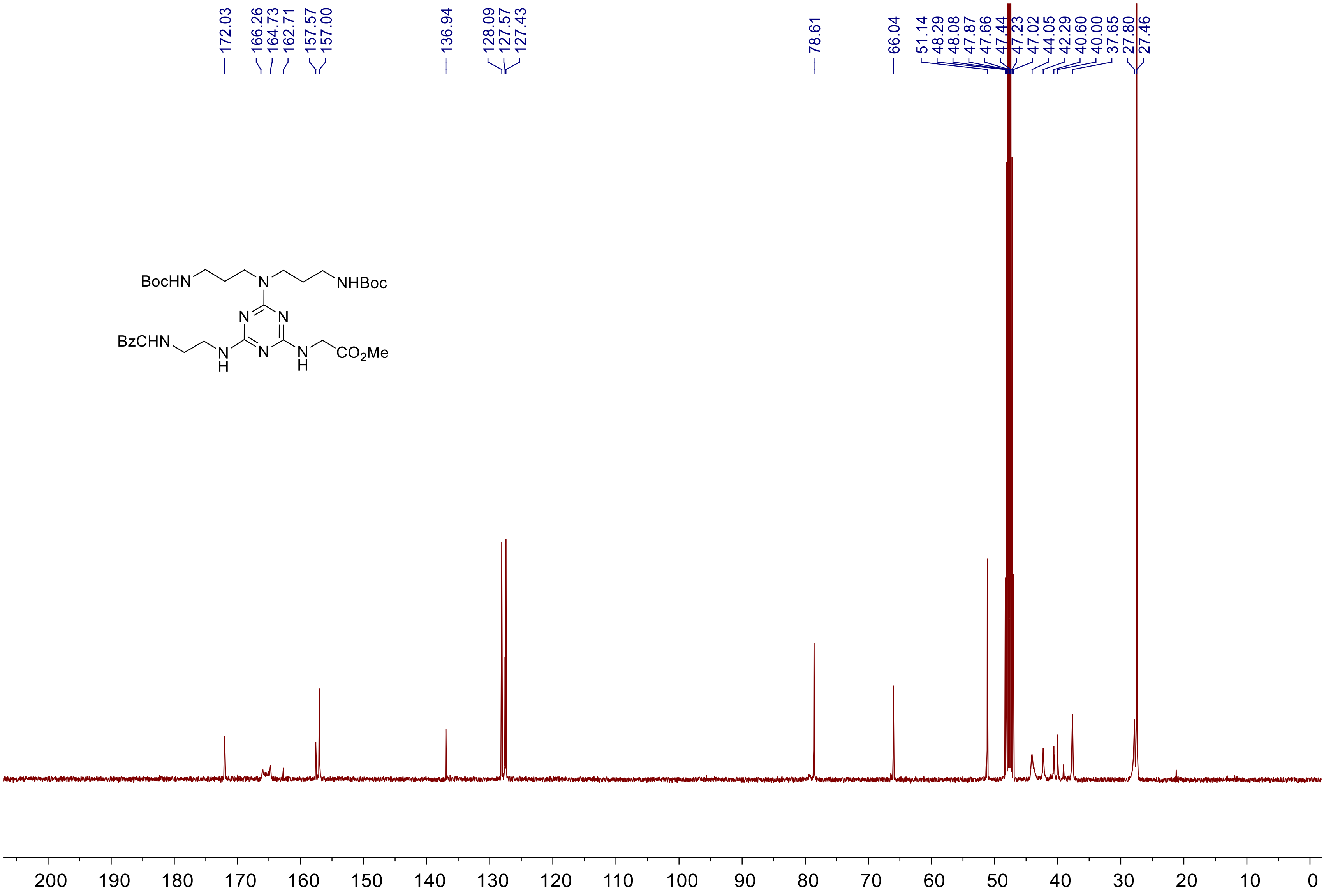

^{13}C NMR spectrum of **8** (MeOD, 100 MHz)

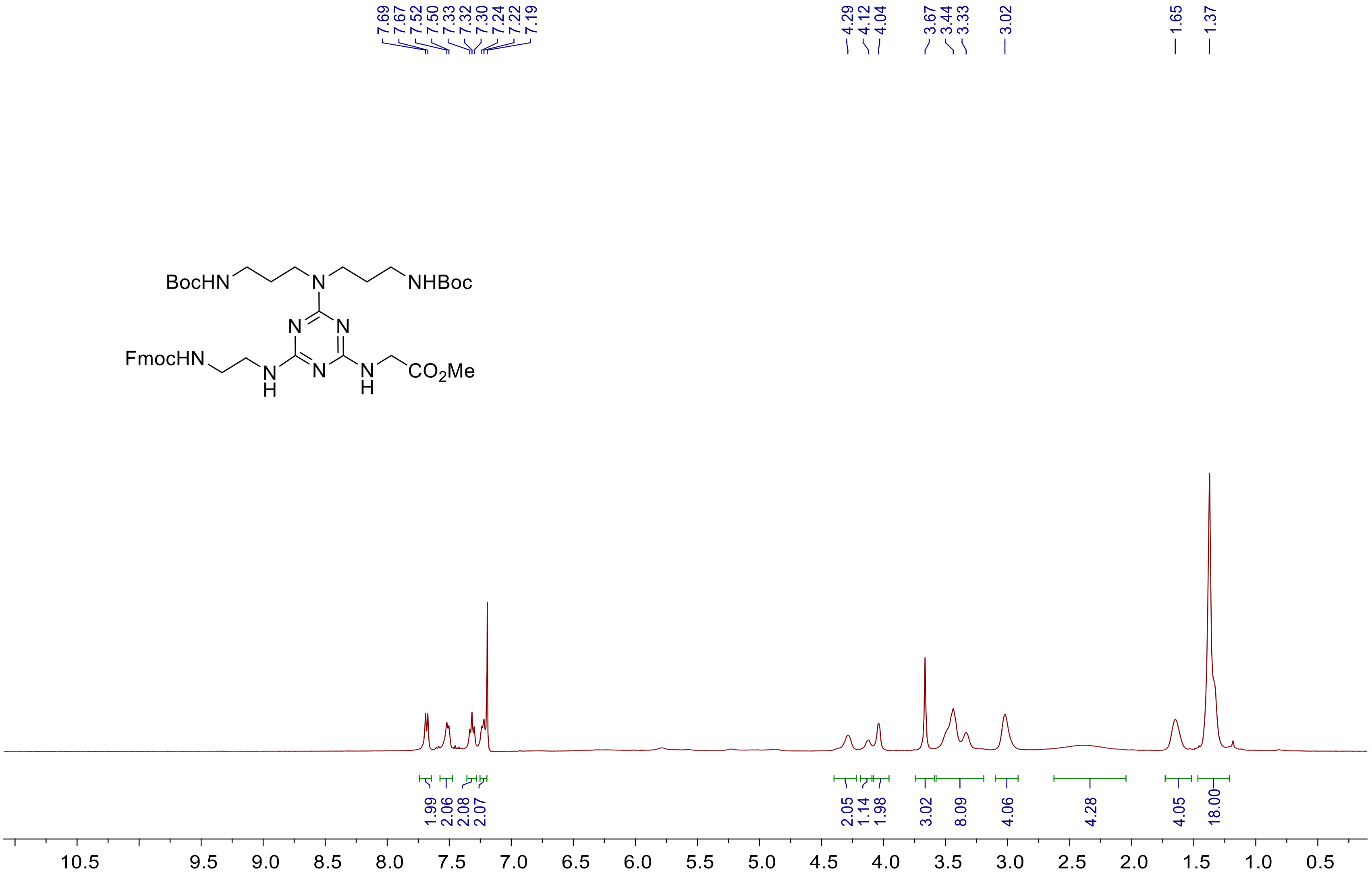

^{1}H NMR spectrum of **9** ($CDCl_3$, 400 MHz)

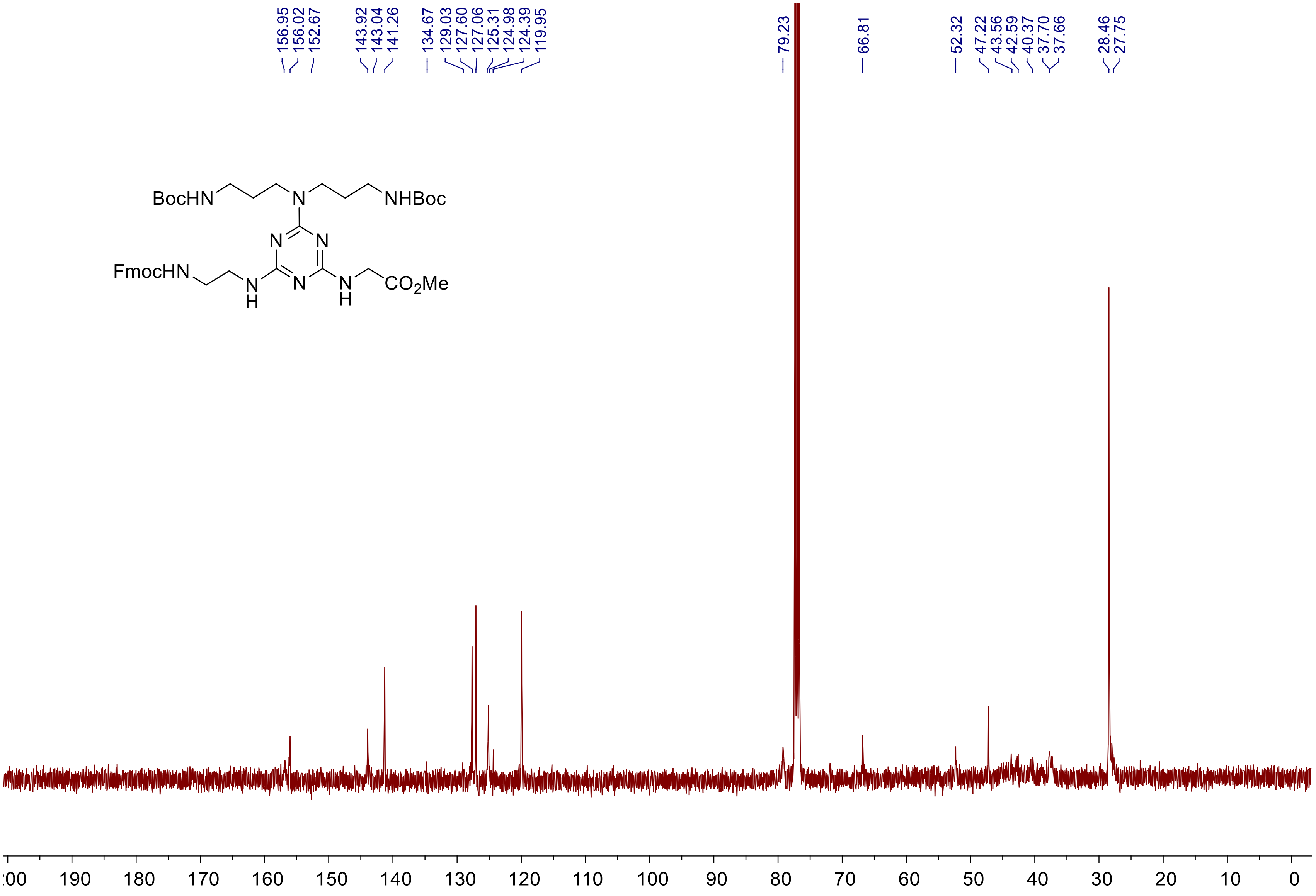

^{13}C NMR spectrum of **9** ($CDCl_3$, 100 MHz)

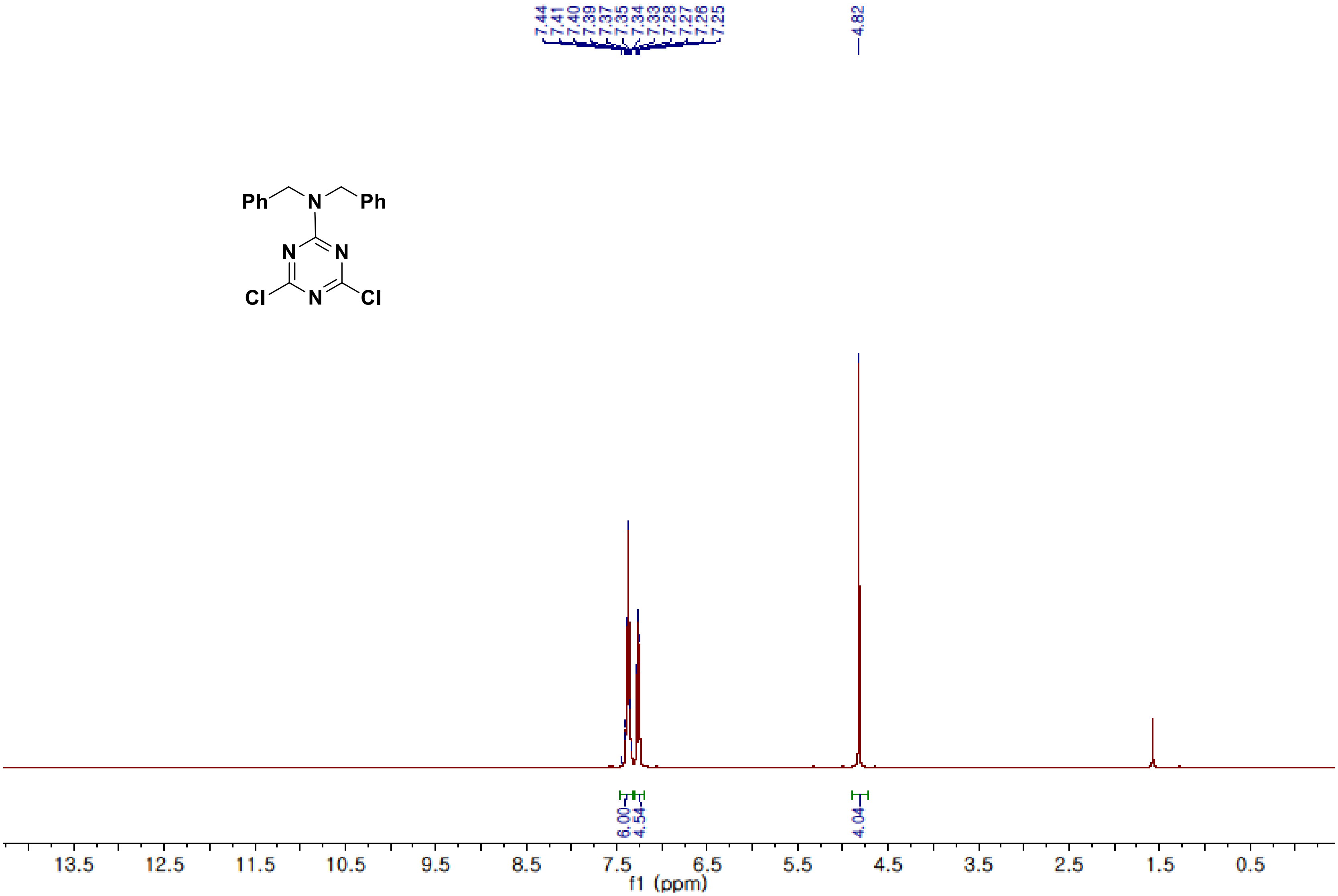

^{1}H NMR spectrum of **10a** ($CDCl_3$, 400 MHz)

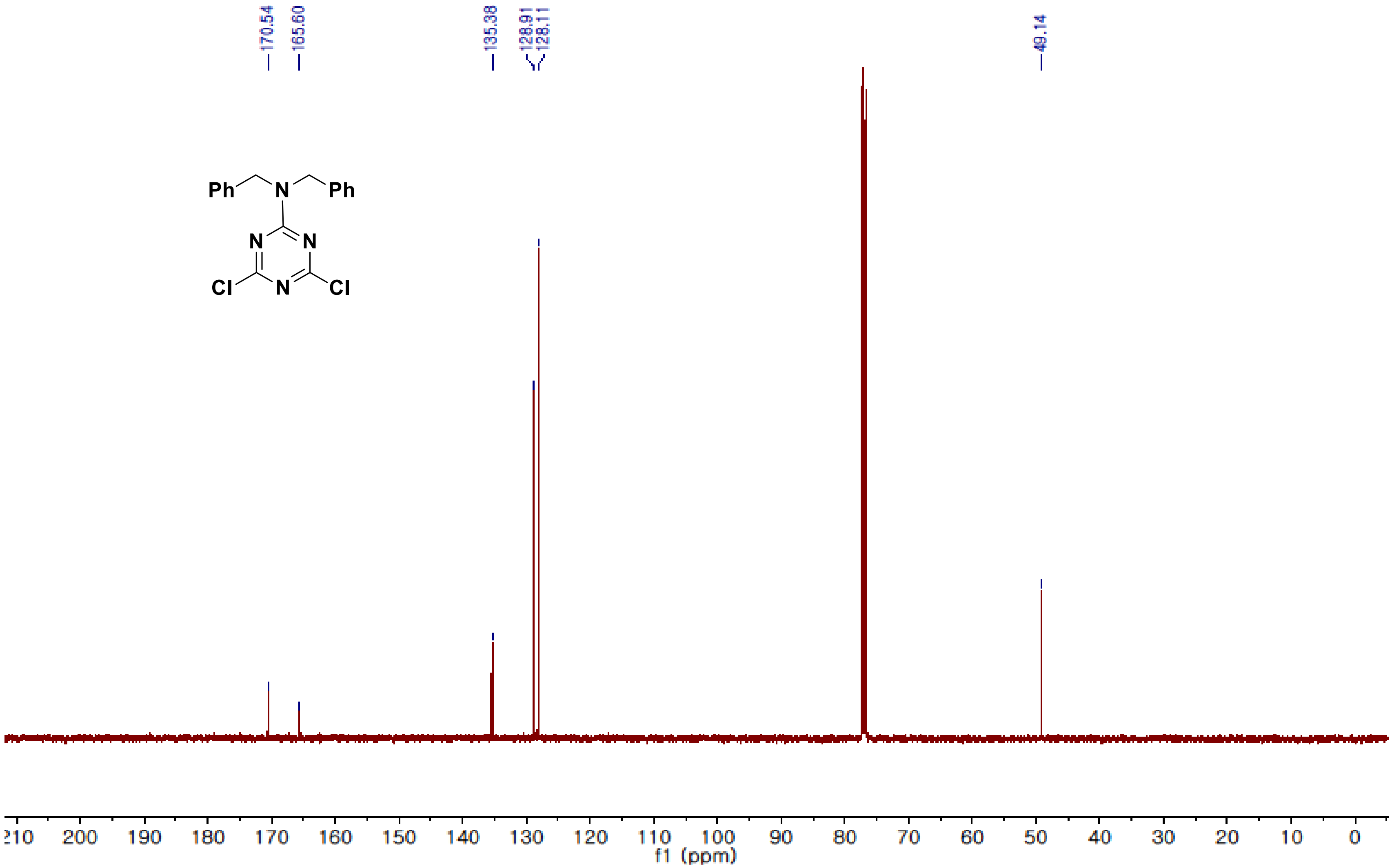

^{13}C NMR spectrum of **10a** ($CDCl_3$, 100 MHz)

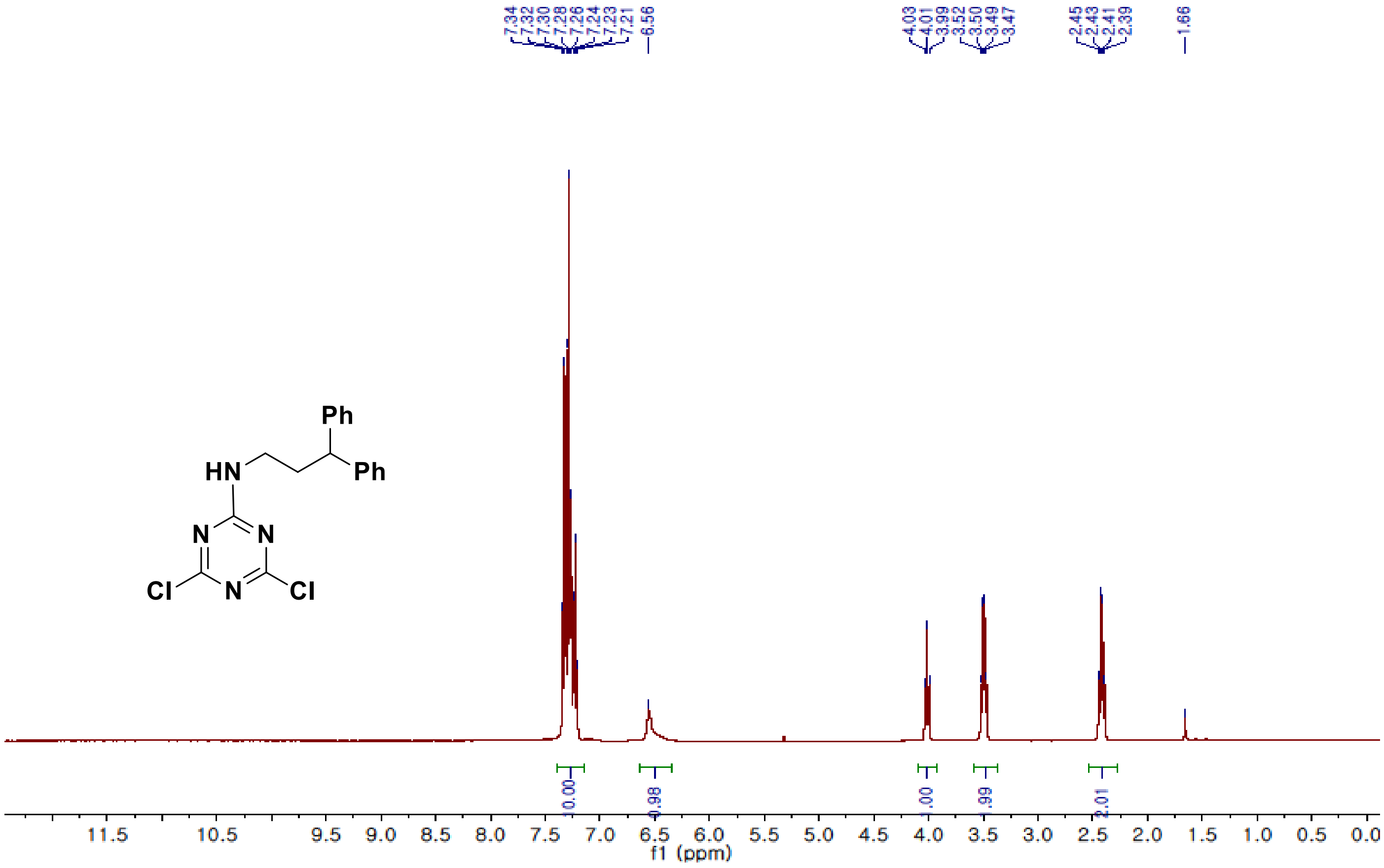

^{1}H NMR spectrum of **10b** ($CDCl_3$, 400 MHz)

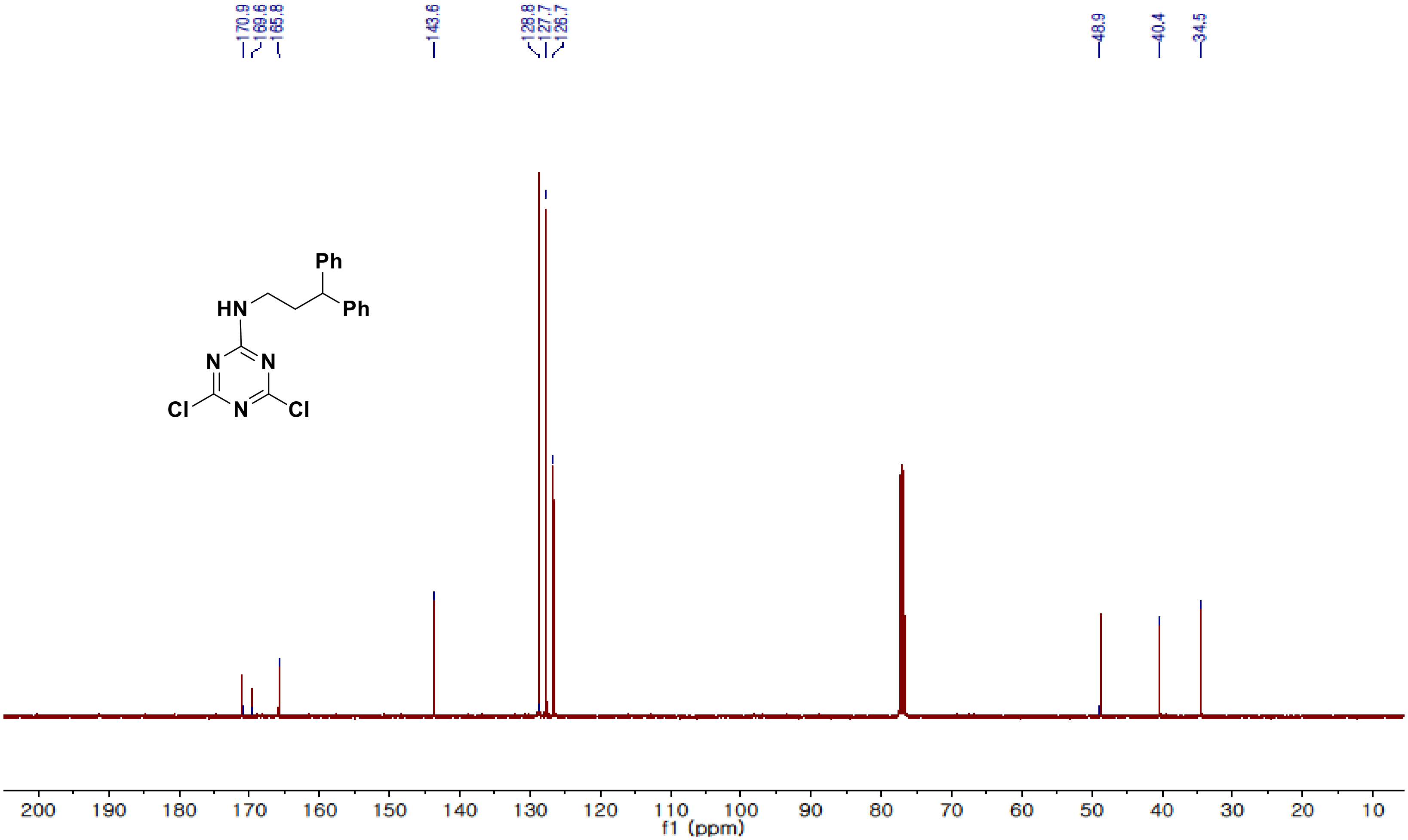

^{13}C NMR spectrum of **10b** ($CDCl_3$, 100 MHz)

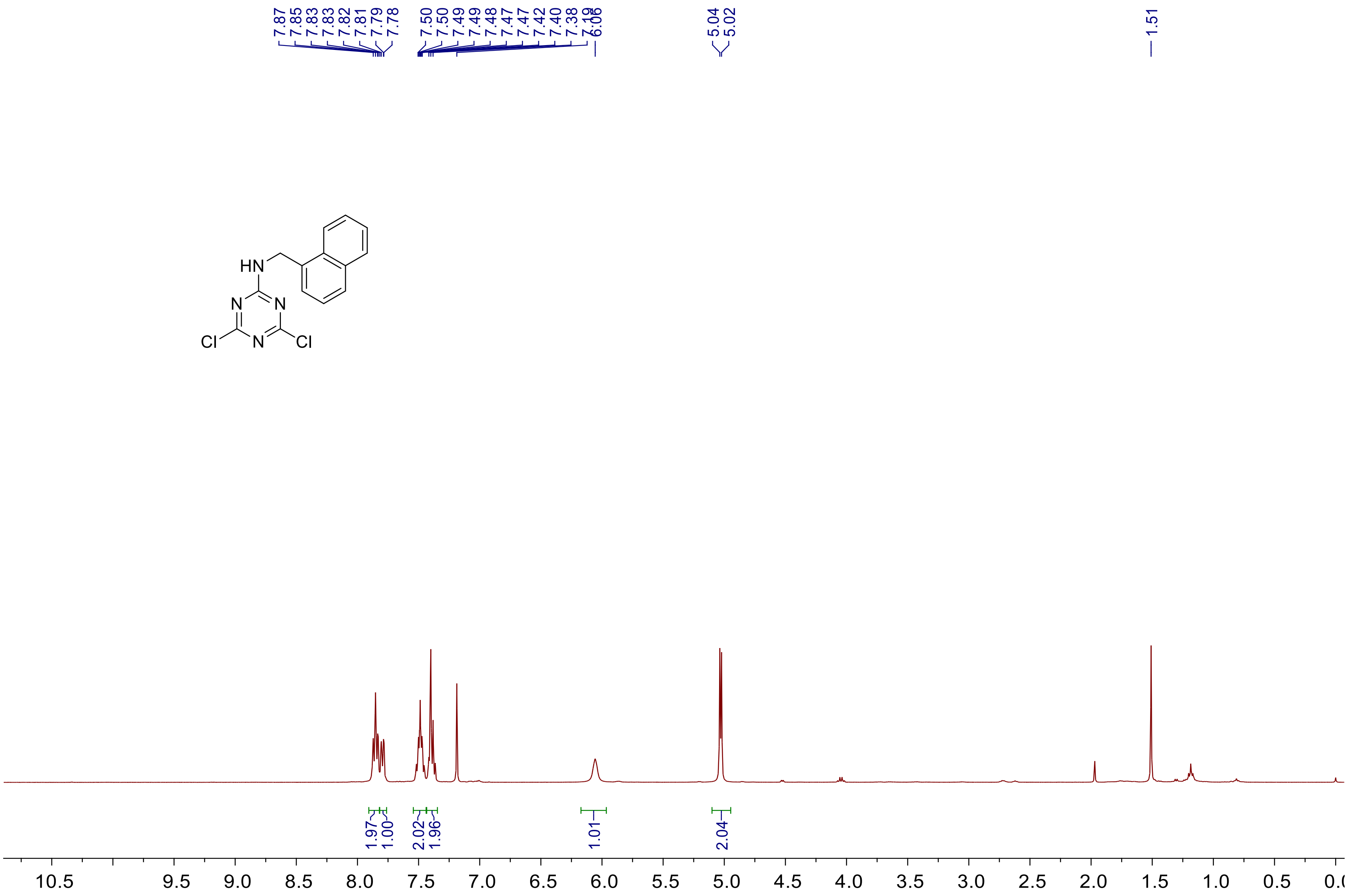

^{1}H NMR spectrum of **10c** ($CDCl_3$, 400 MHz)

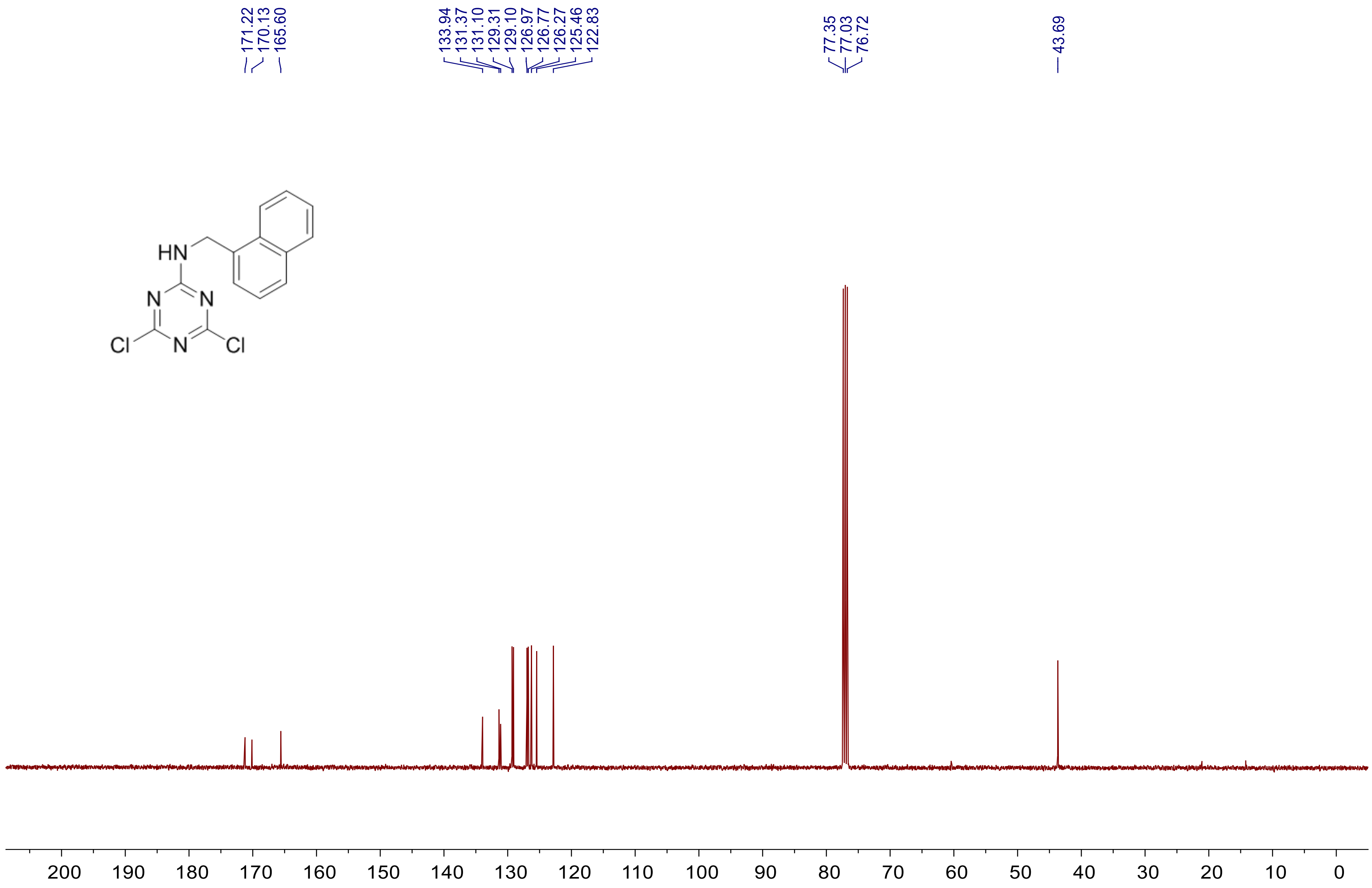

^{13}C NMR spectrum of **10c** ($CDCl_3$, 100 MHz)

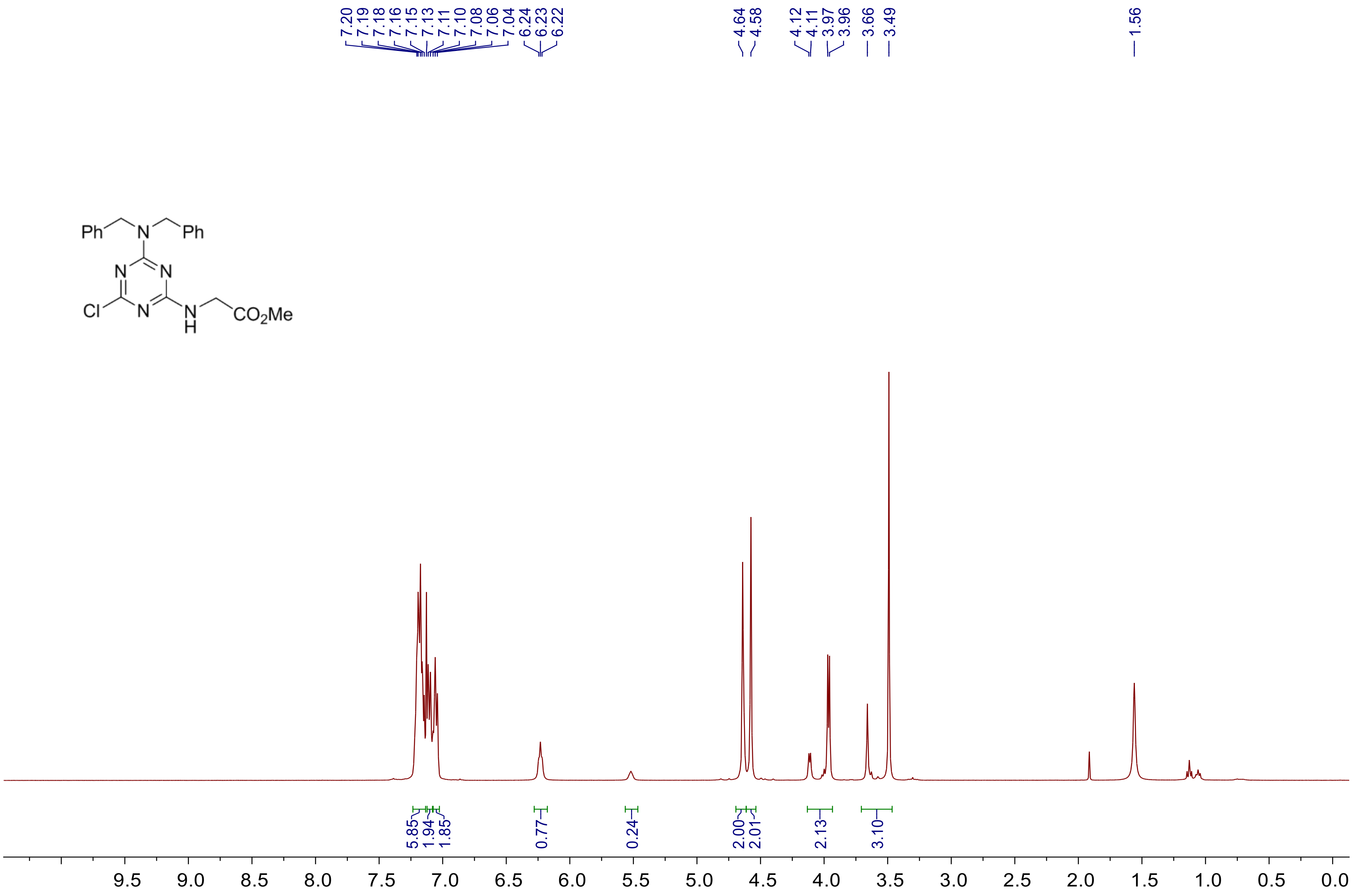

^1H NMR spectrum of **11a** ($CDCl_3$, 400 MHz)

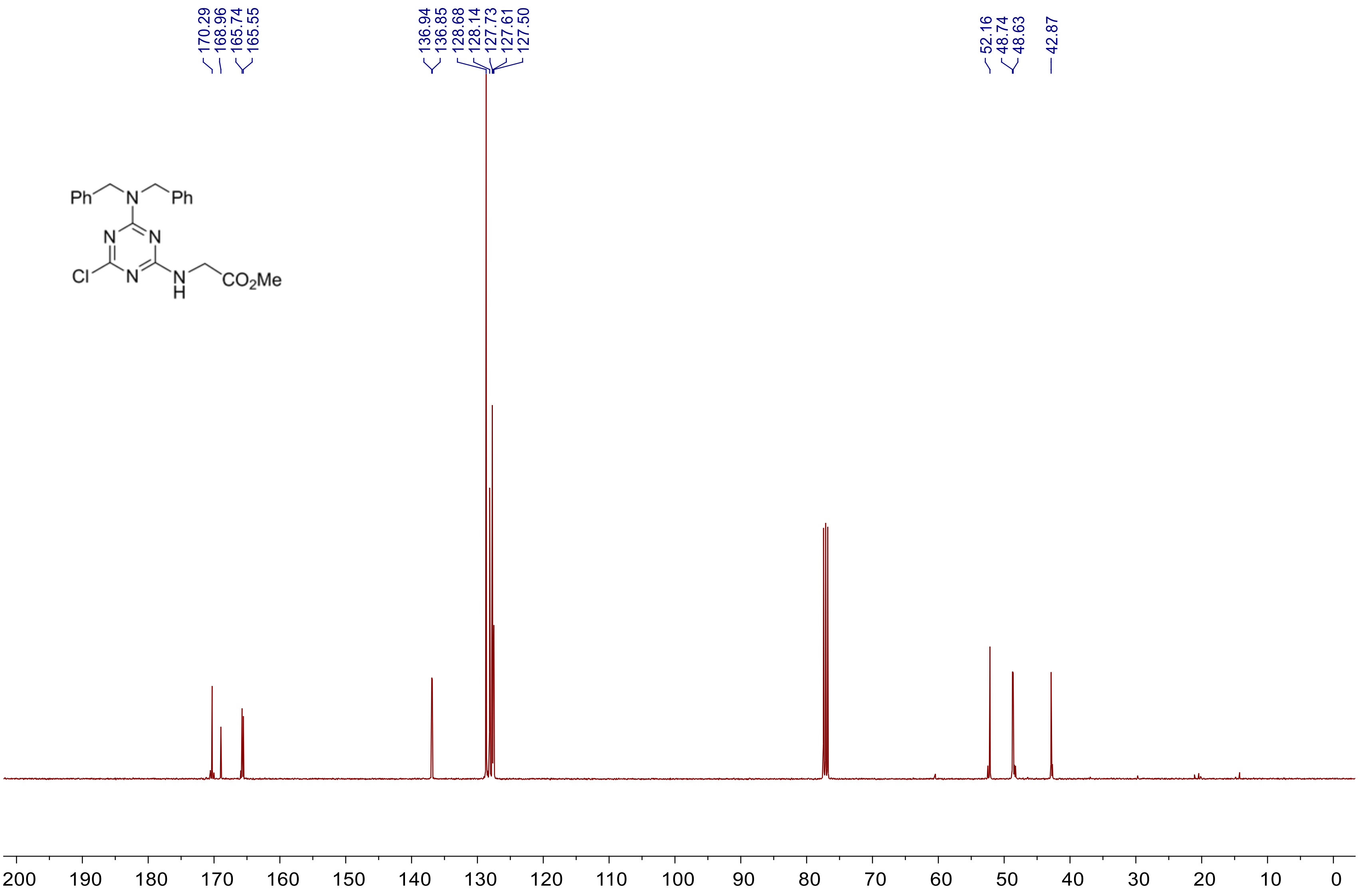

^{13}C NMR spectrum of **11a** ($CDCl_3$, 100 MHz)

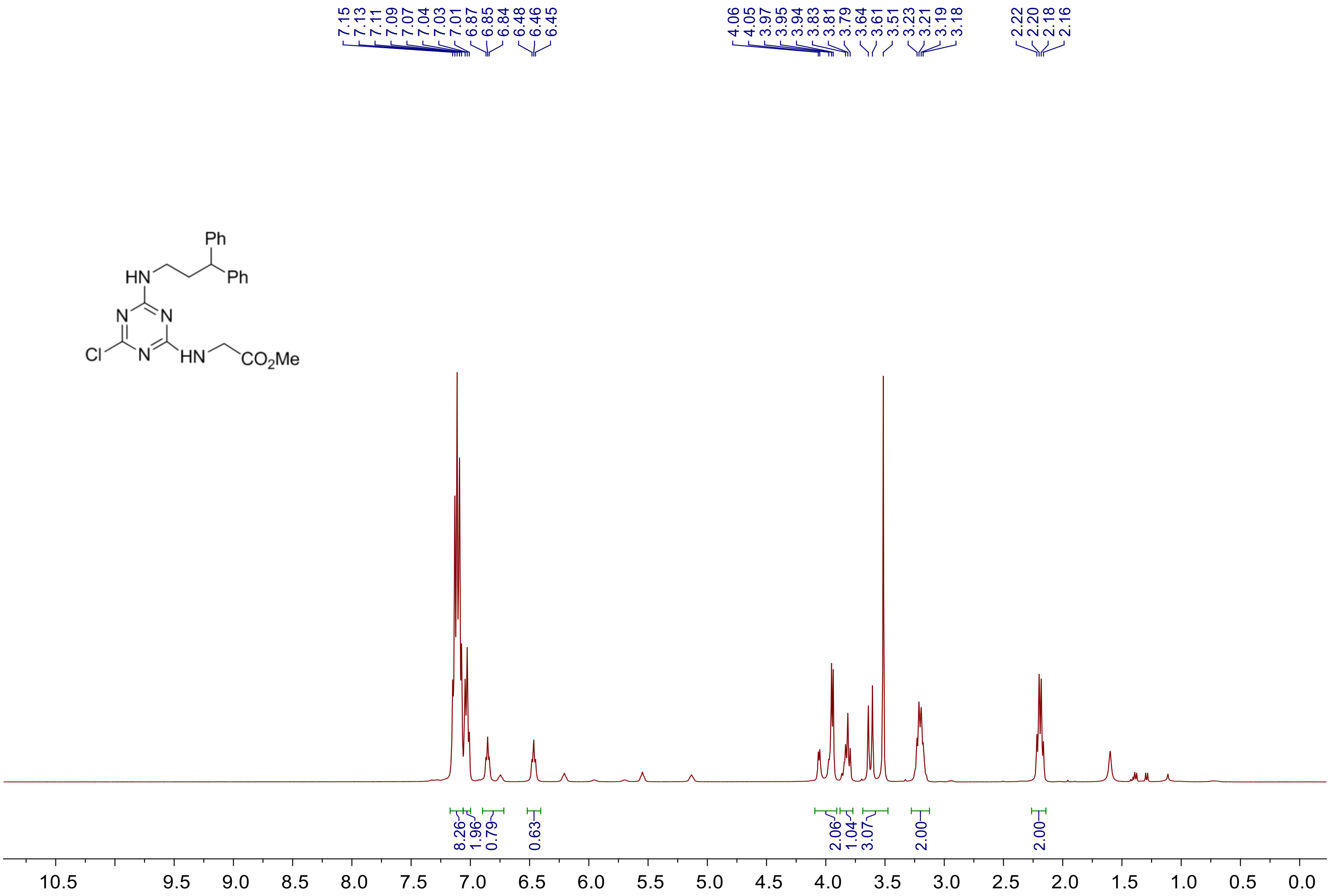

^{1}H NMR spectrum of **11b** ($CDCl_3$, 400 MHz)

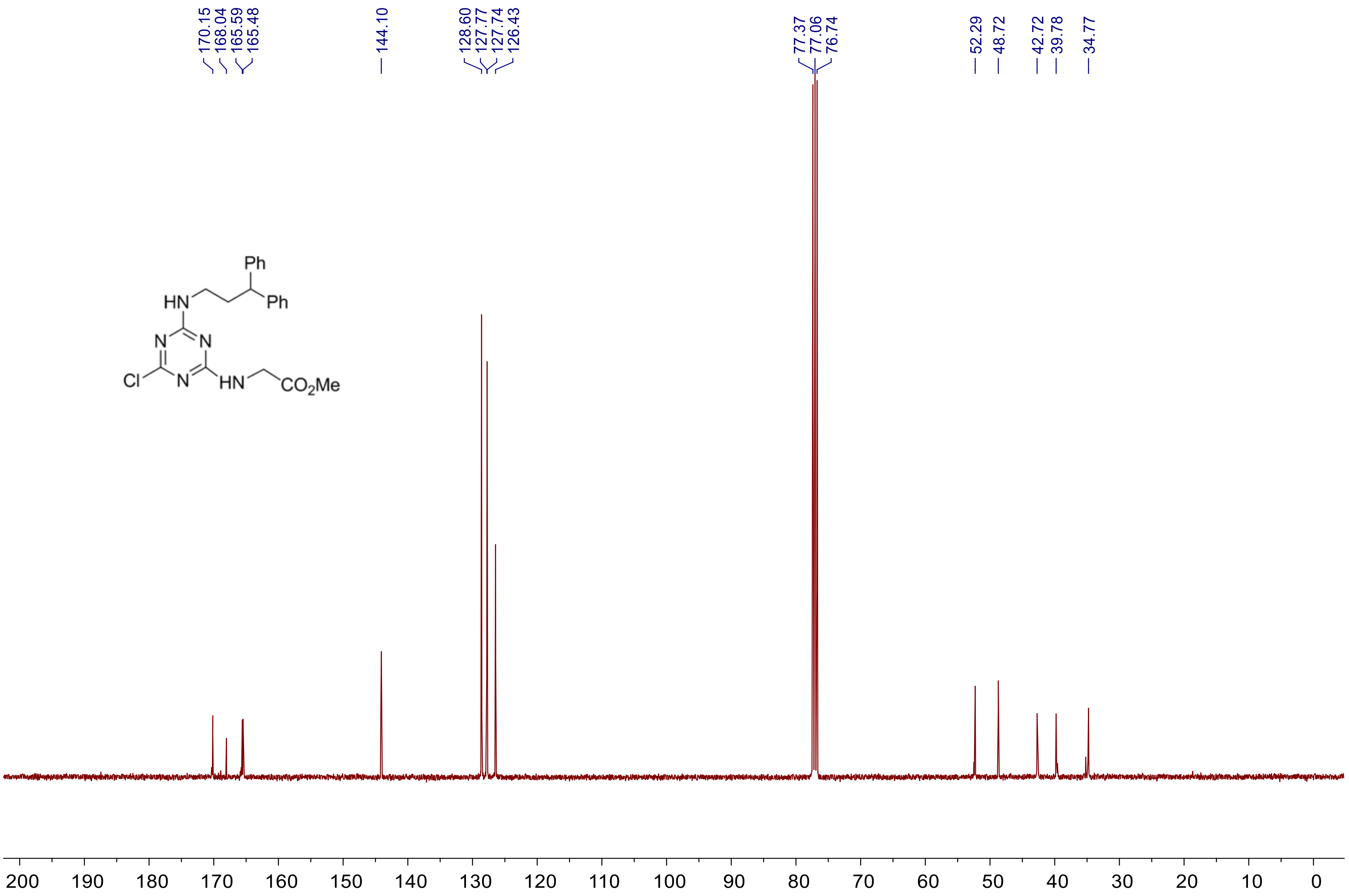

^{13}C NMR spectrum of **11b** ($CDCl_3$, 100 MHz)

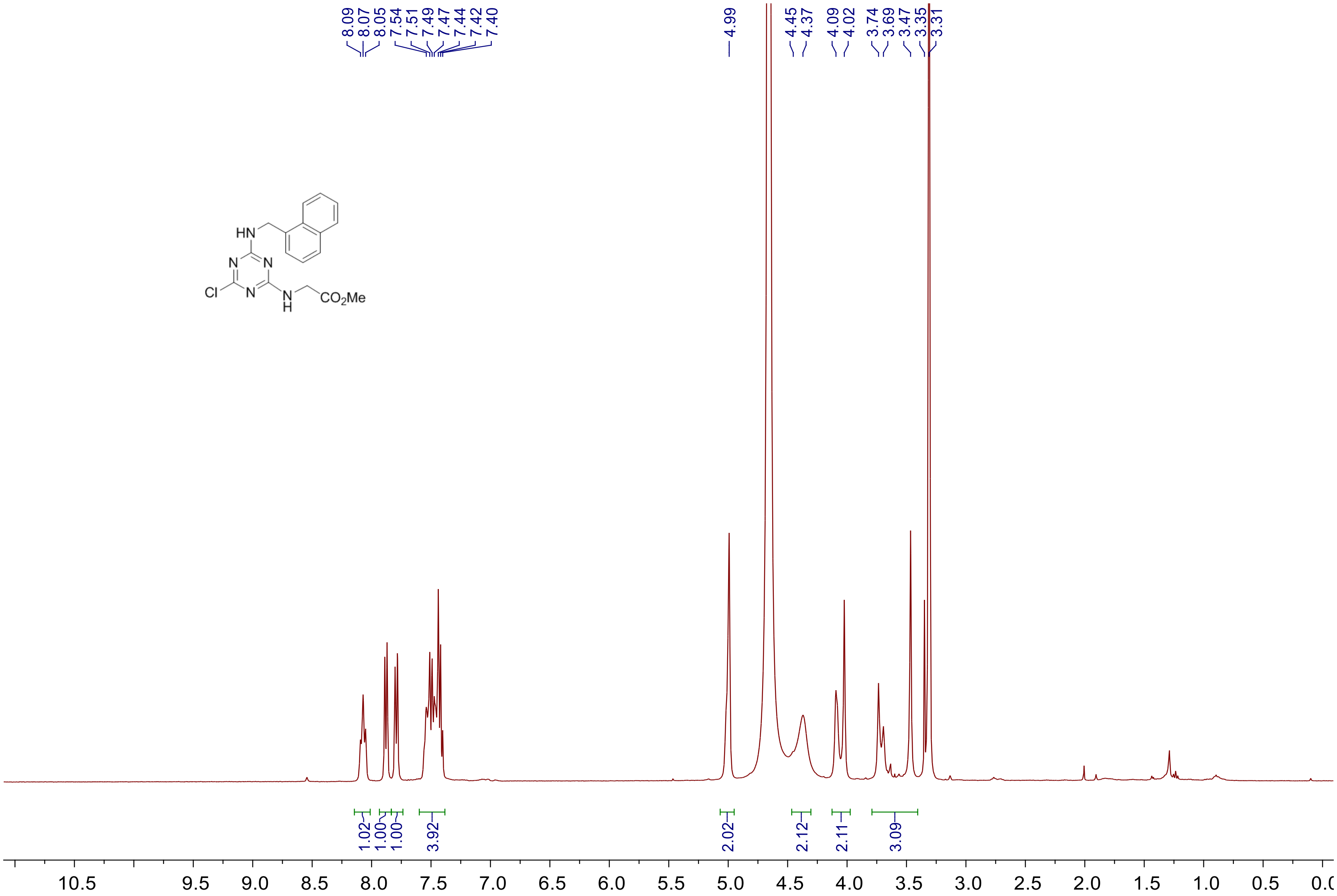

^{1}H NMR spectrum of **11c** (MeOD, 400 MHz)

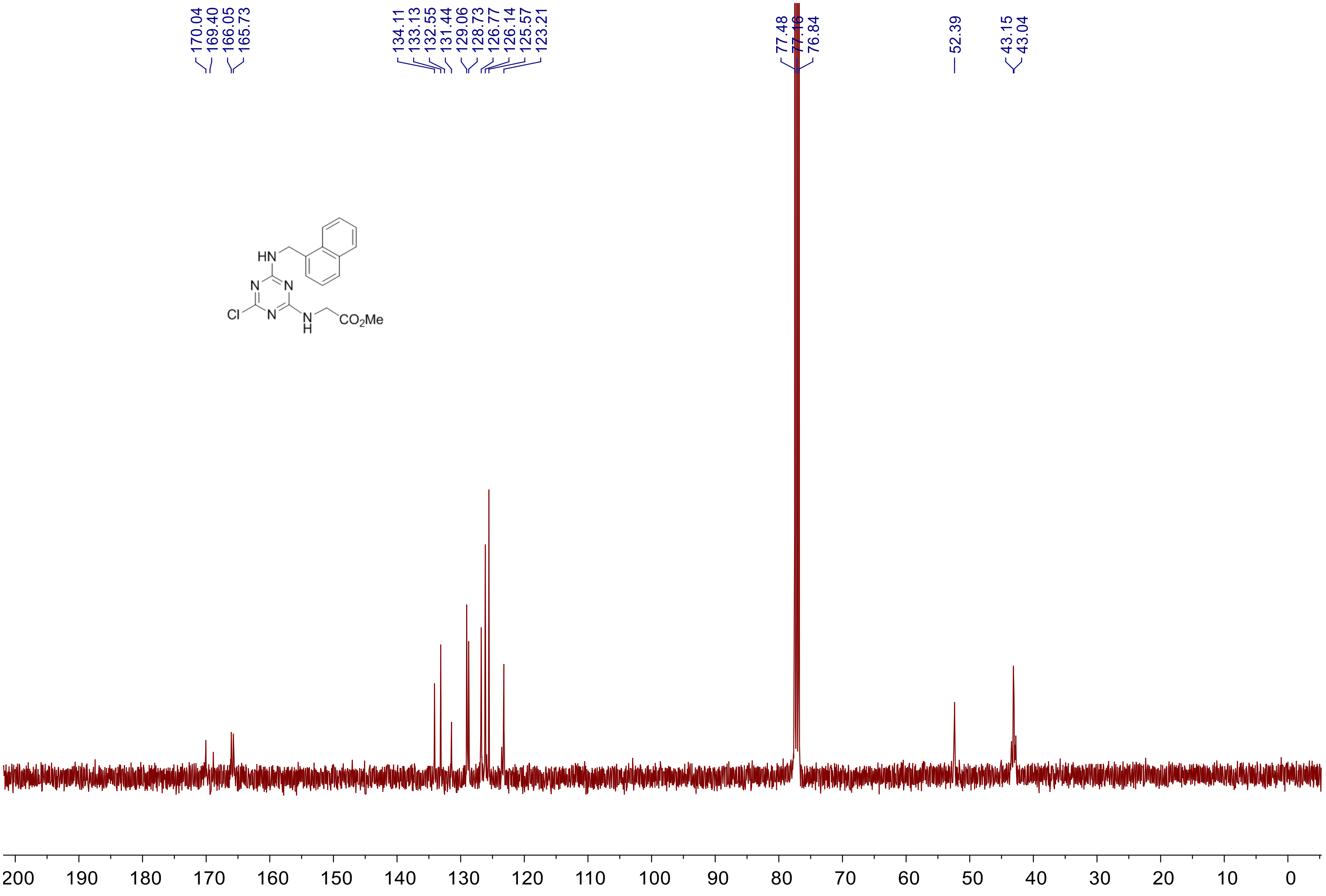

^{13}C NMR spectrum of **11c** ($CDCl_3$, 100 MHz)

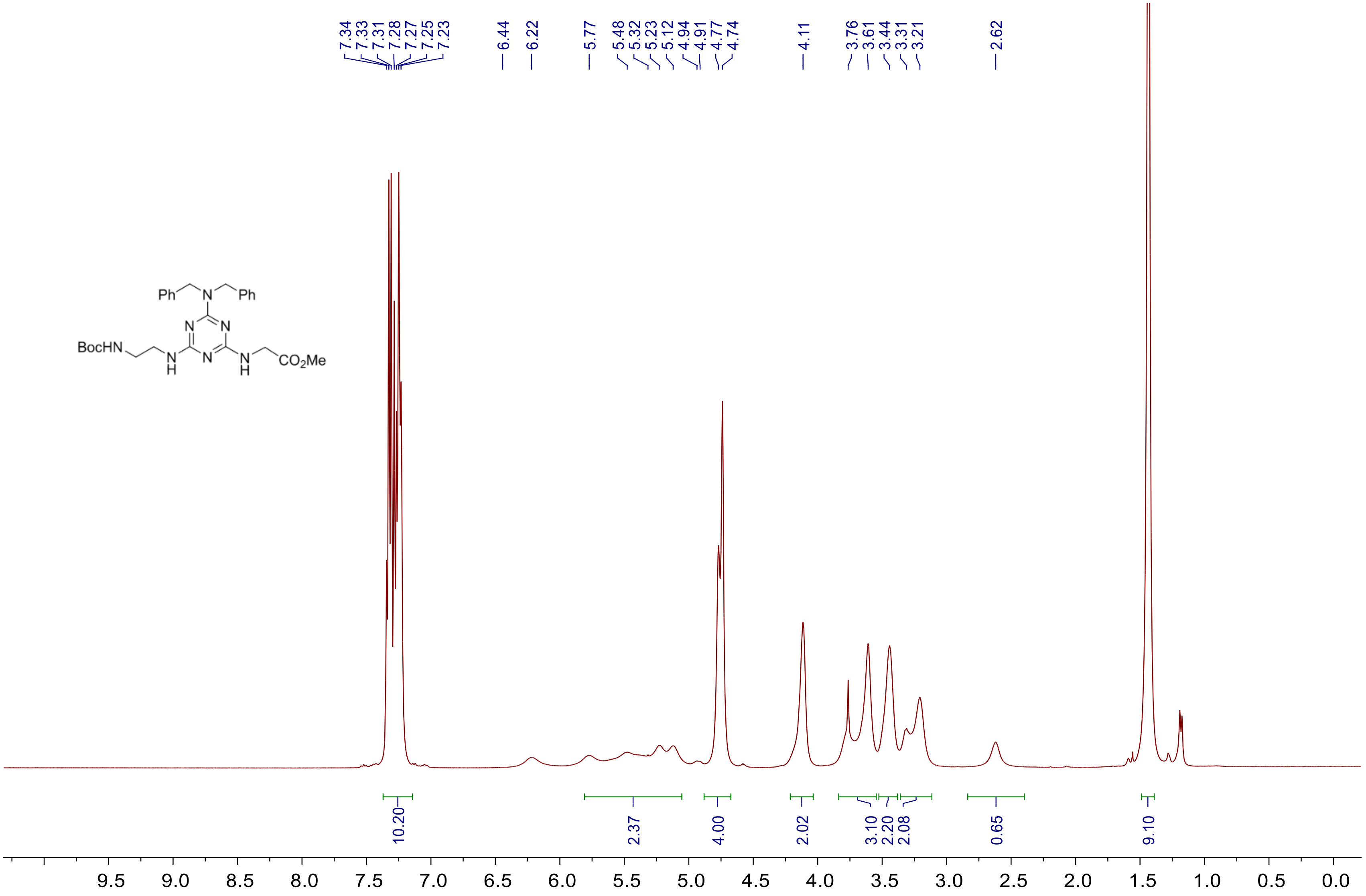

^{1}H NMR spectrum of **12a** ($CDCl_3$, 400 MHz)

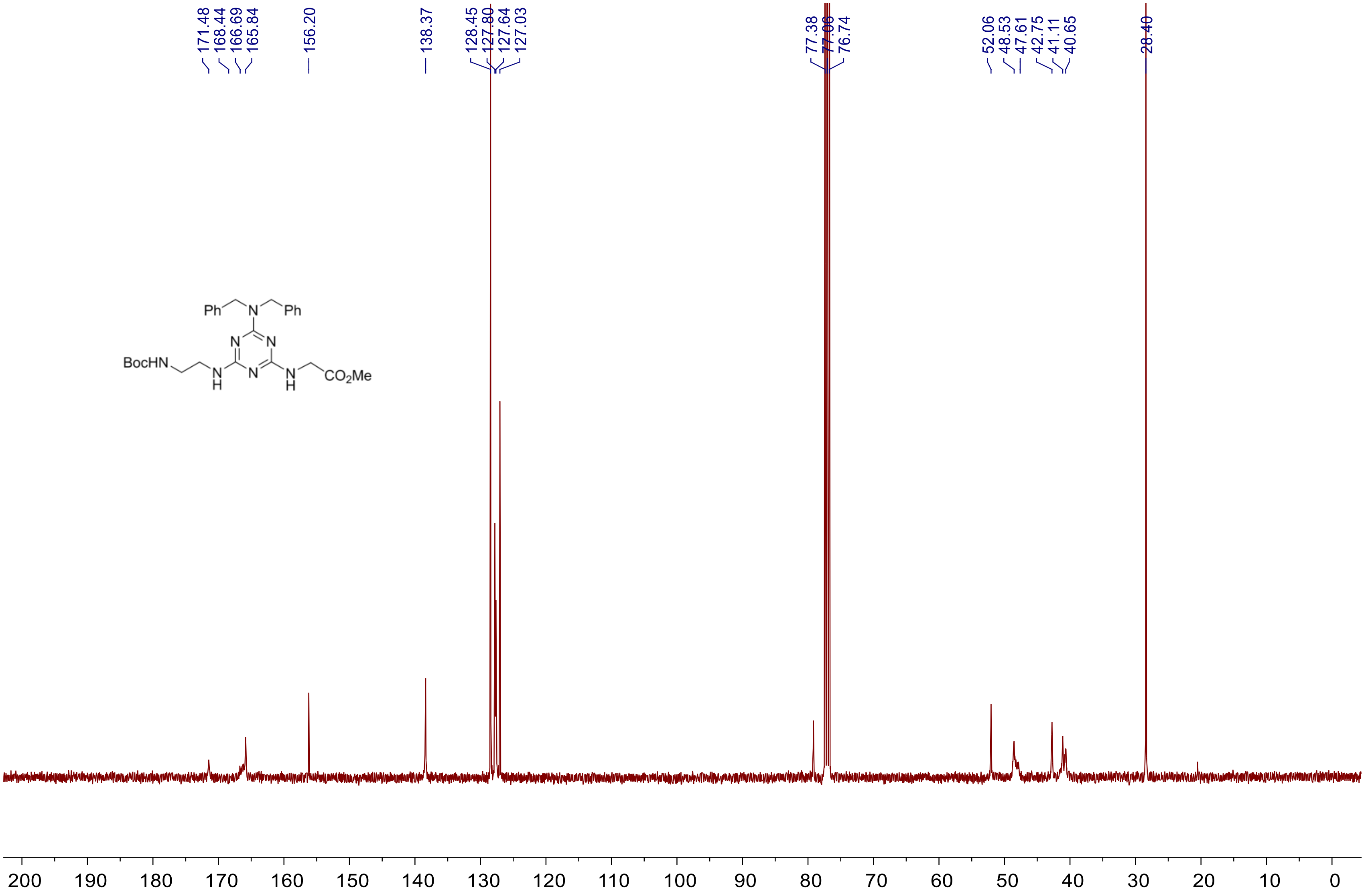

^{13}C NMR spectrum of **12a** ($CDCl_3$, 100 MHz)

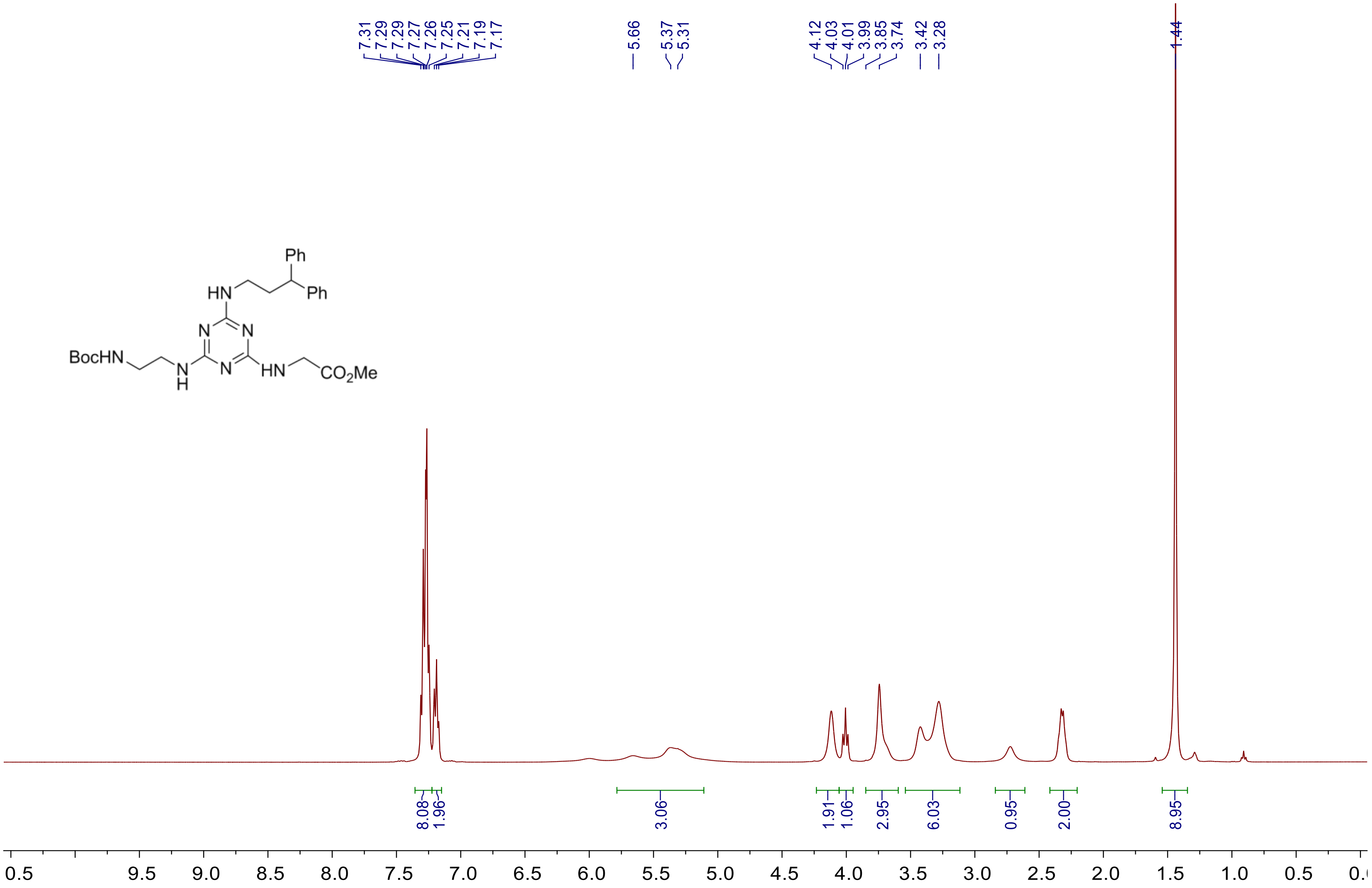

^{1}H NMR spectrum of **12b** ($CDCl_3$, 400 MHz)

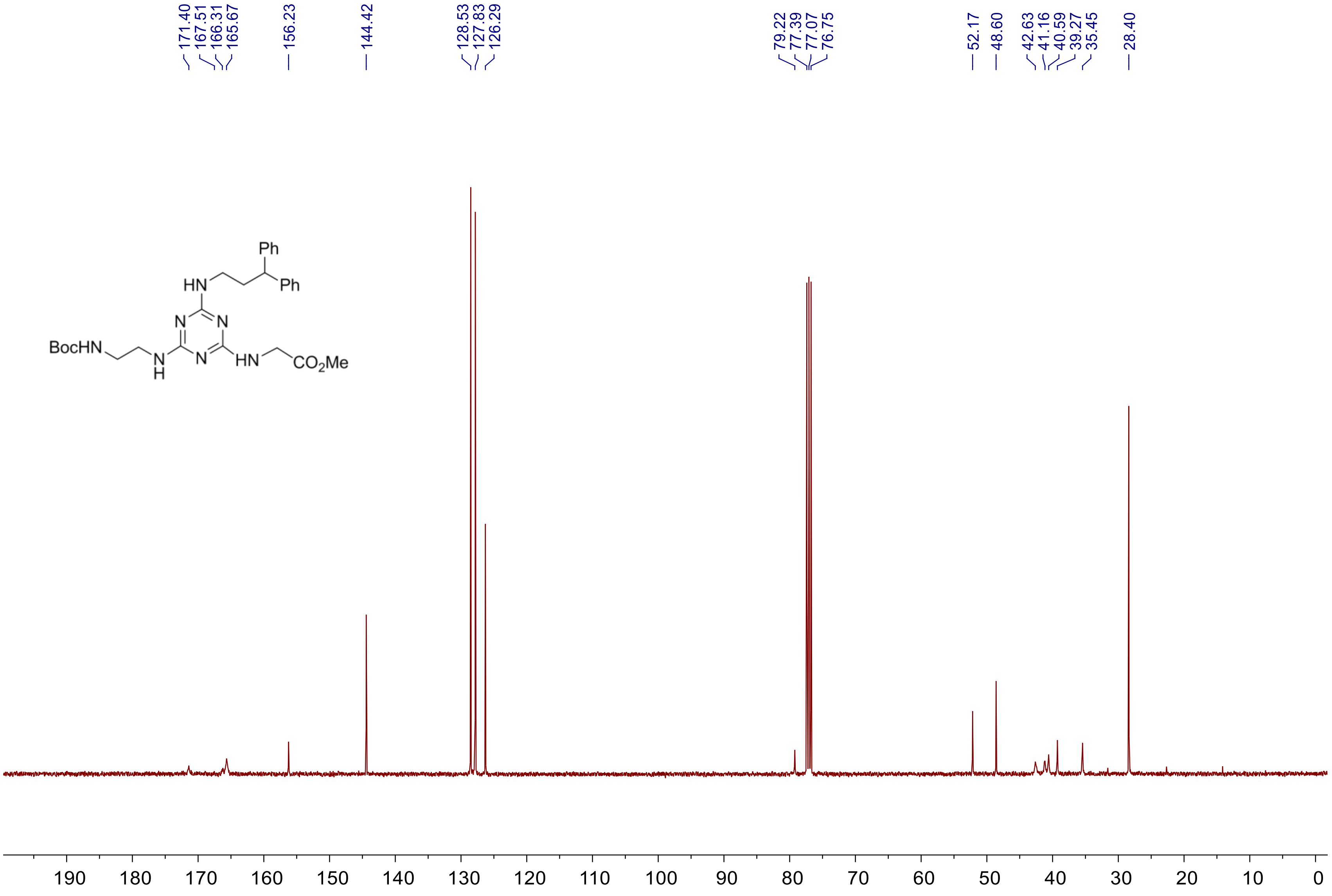

^{13}C NMR spectrum of **12b** ($CDCl_3$, 100 MHz)

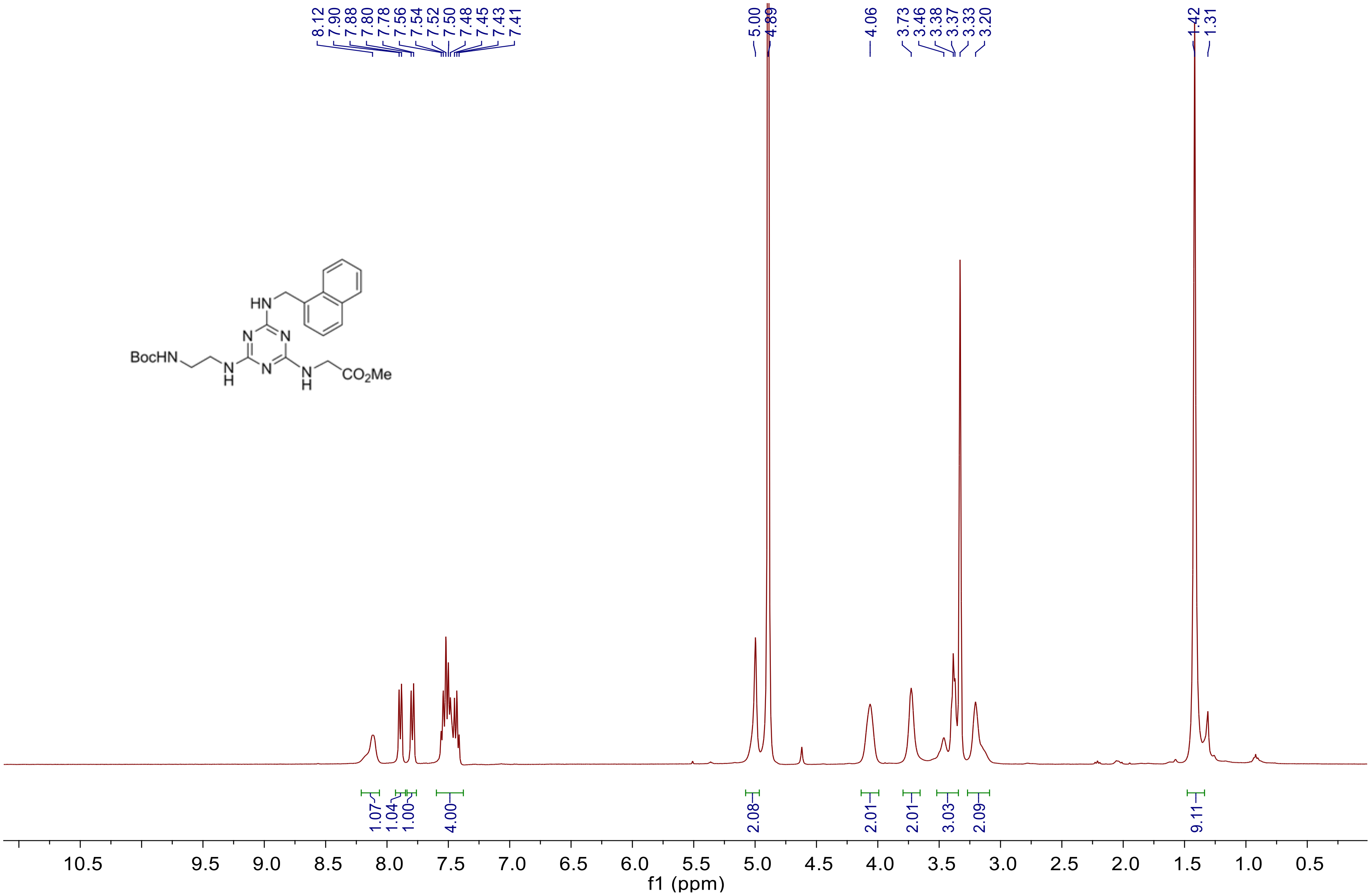

^{1}H NMR spectrum of **12c** (MeOD, 400 MHz)

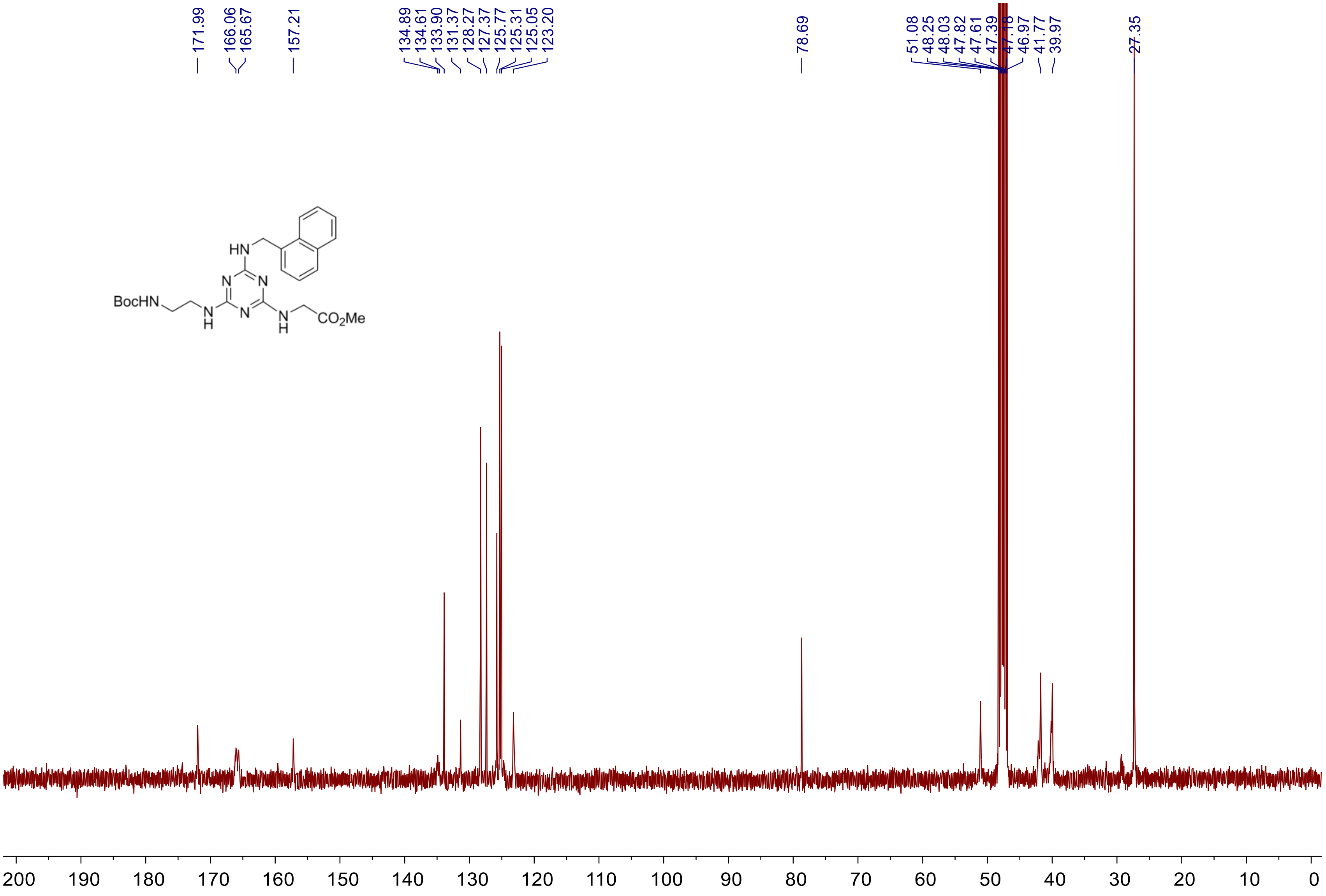

^{13}C NMR spectrum of **12c** (MeOD, 100 MHz)

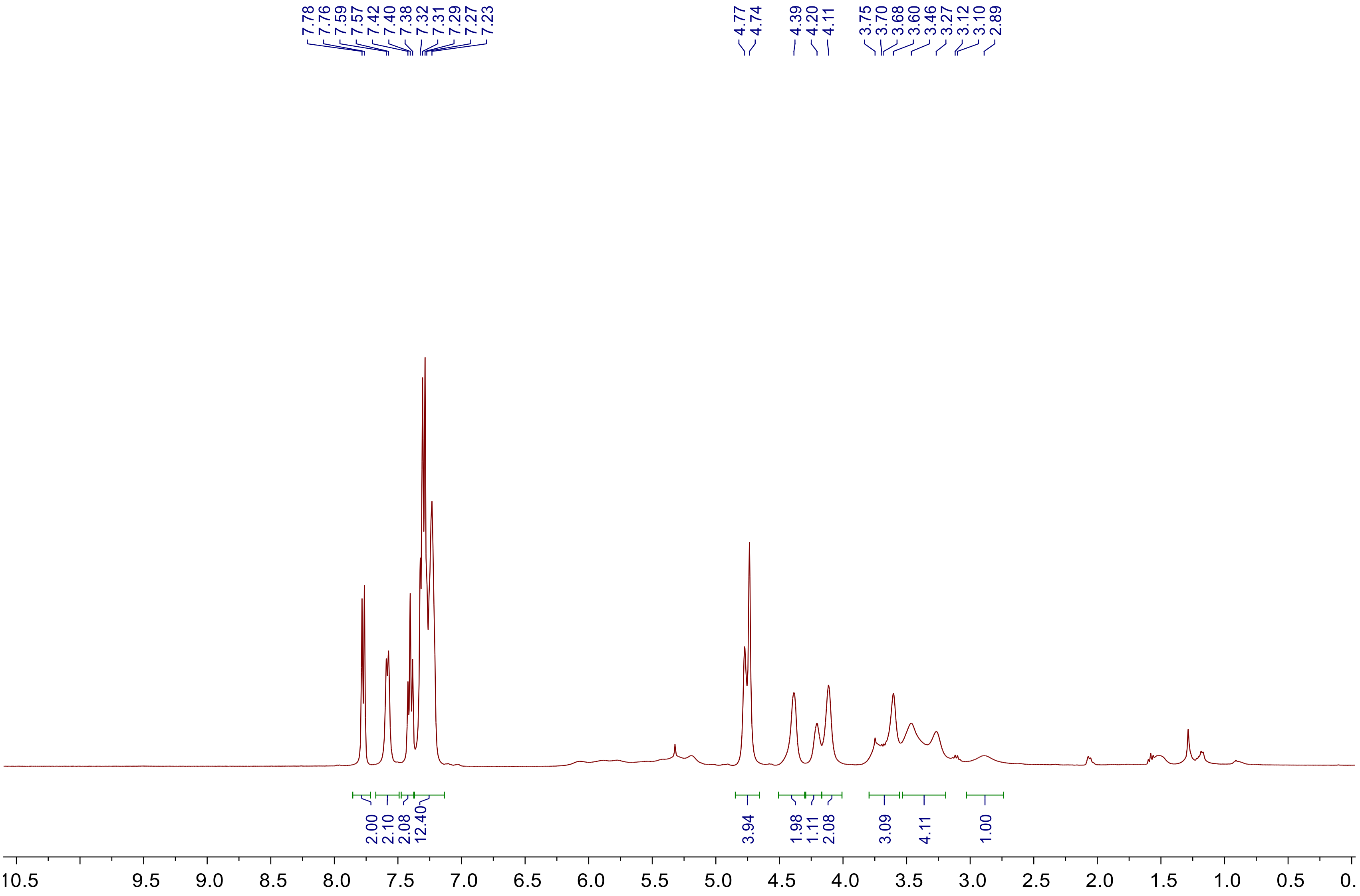

^{1}H NMR spectrum of **13a** ($CDCl_3$, 400 MHz)

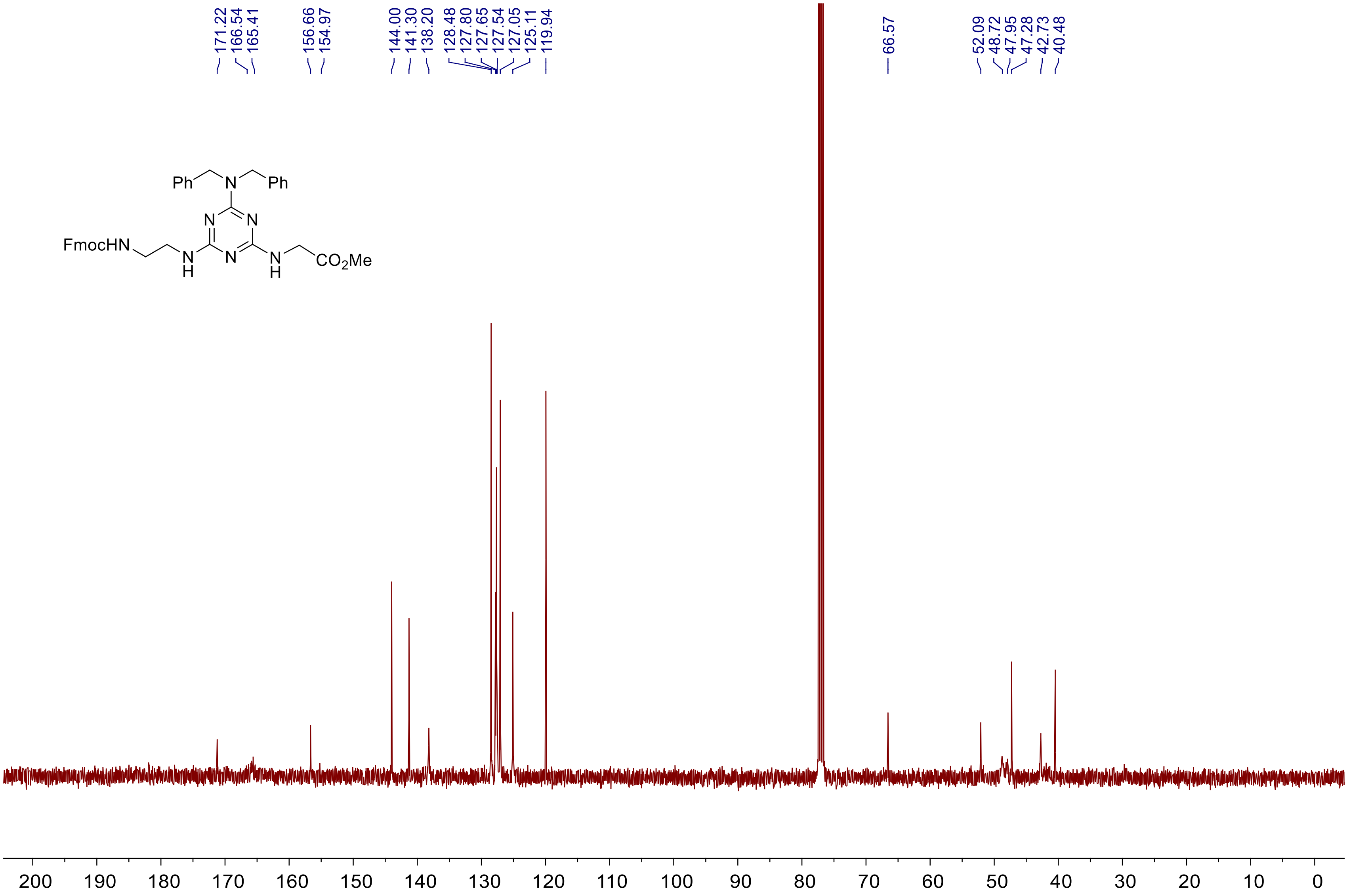

^{13}C NMR spectrum of **13a** ($CDCl_3$, 100 MHz)

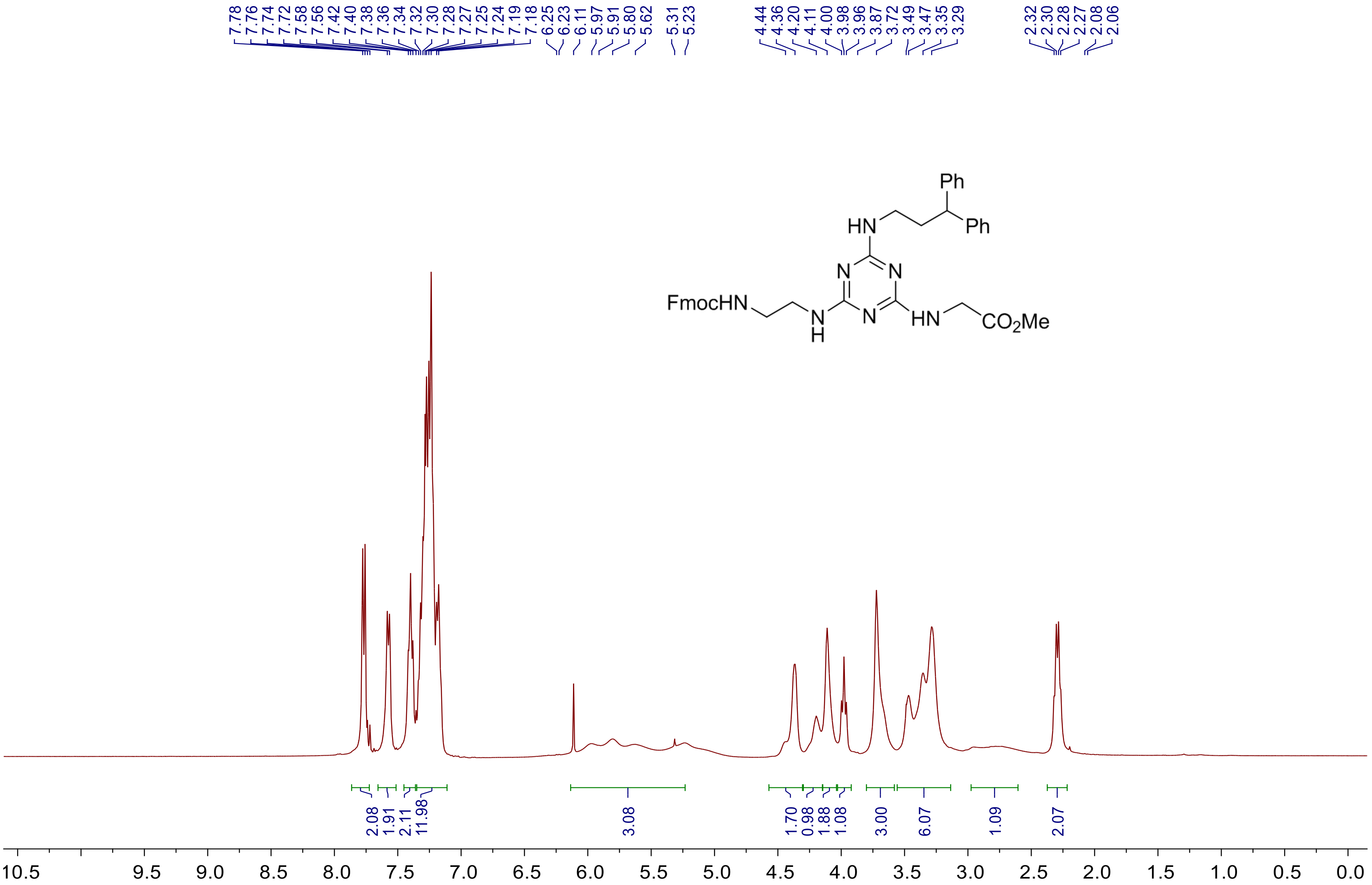

^{1}H NMR spectrum of **13b** ($CDCl_3$, 400 MHz)

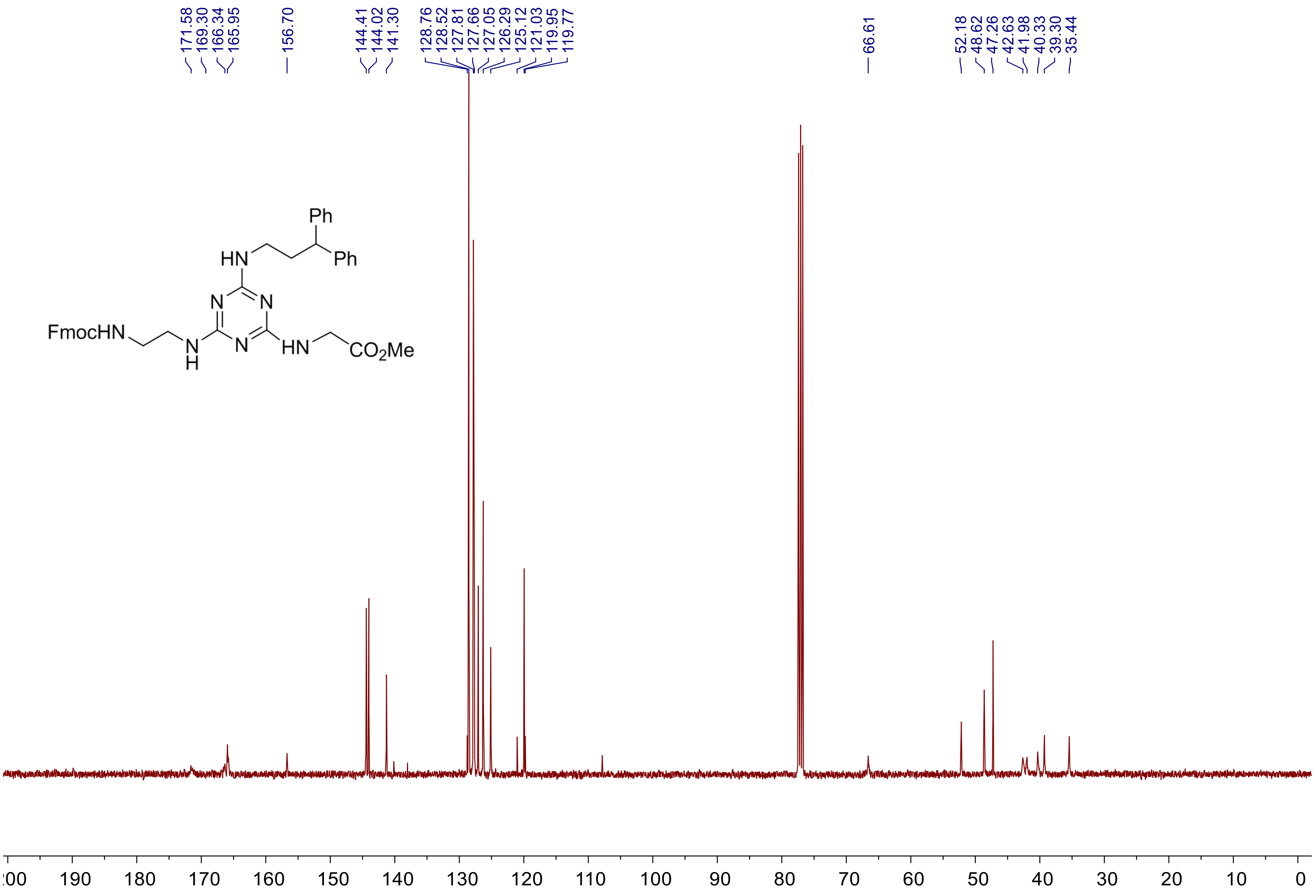

^{13}C NMR spectrum of **13b** ($CDCl_3$, 100 MHz)

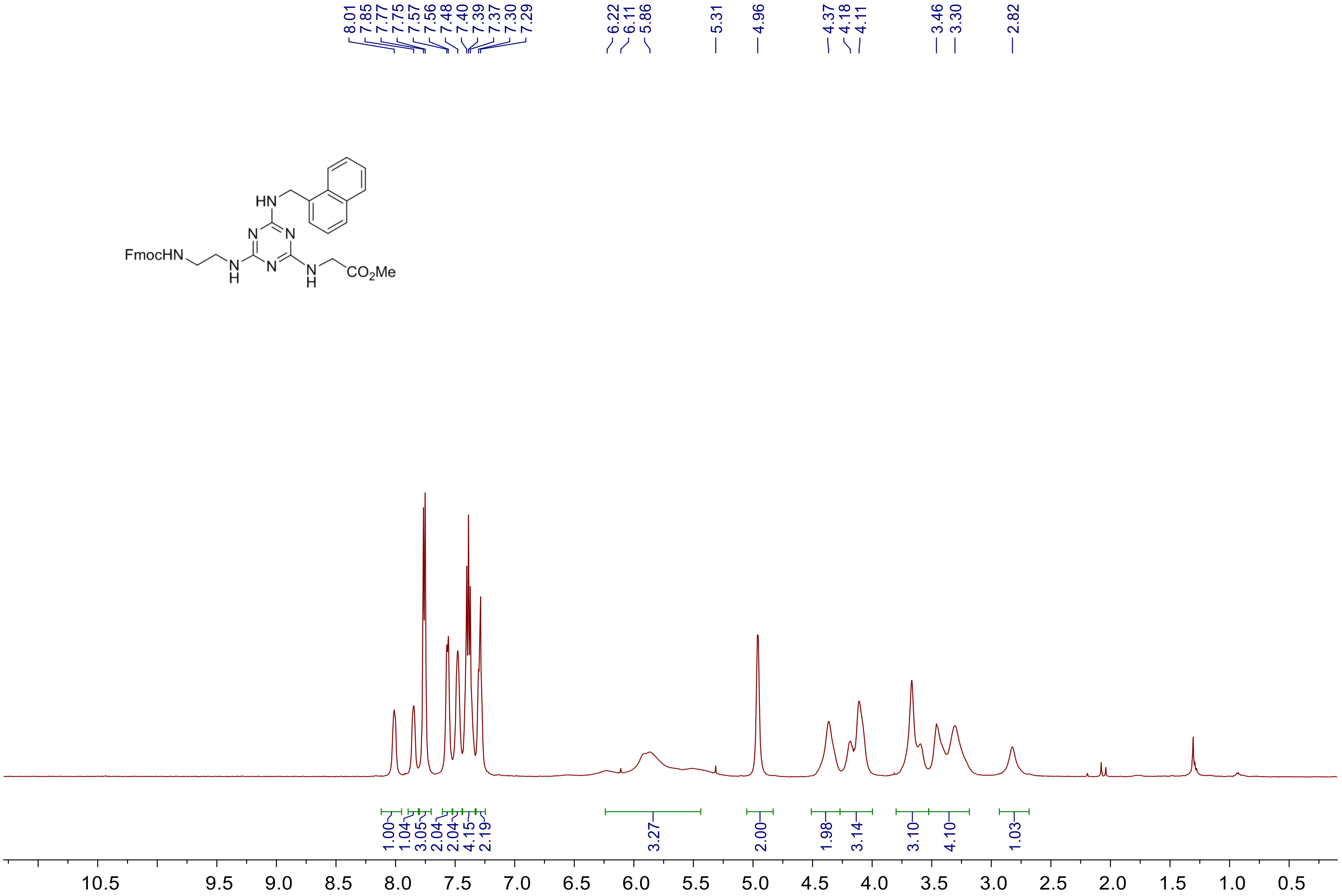

^{1}H NMR spectrum of **13c** ($CDCl_3$, 400 MHz)

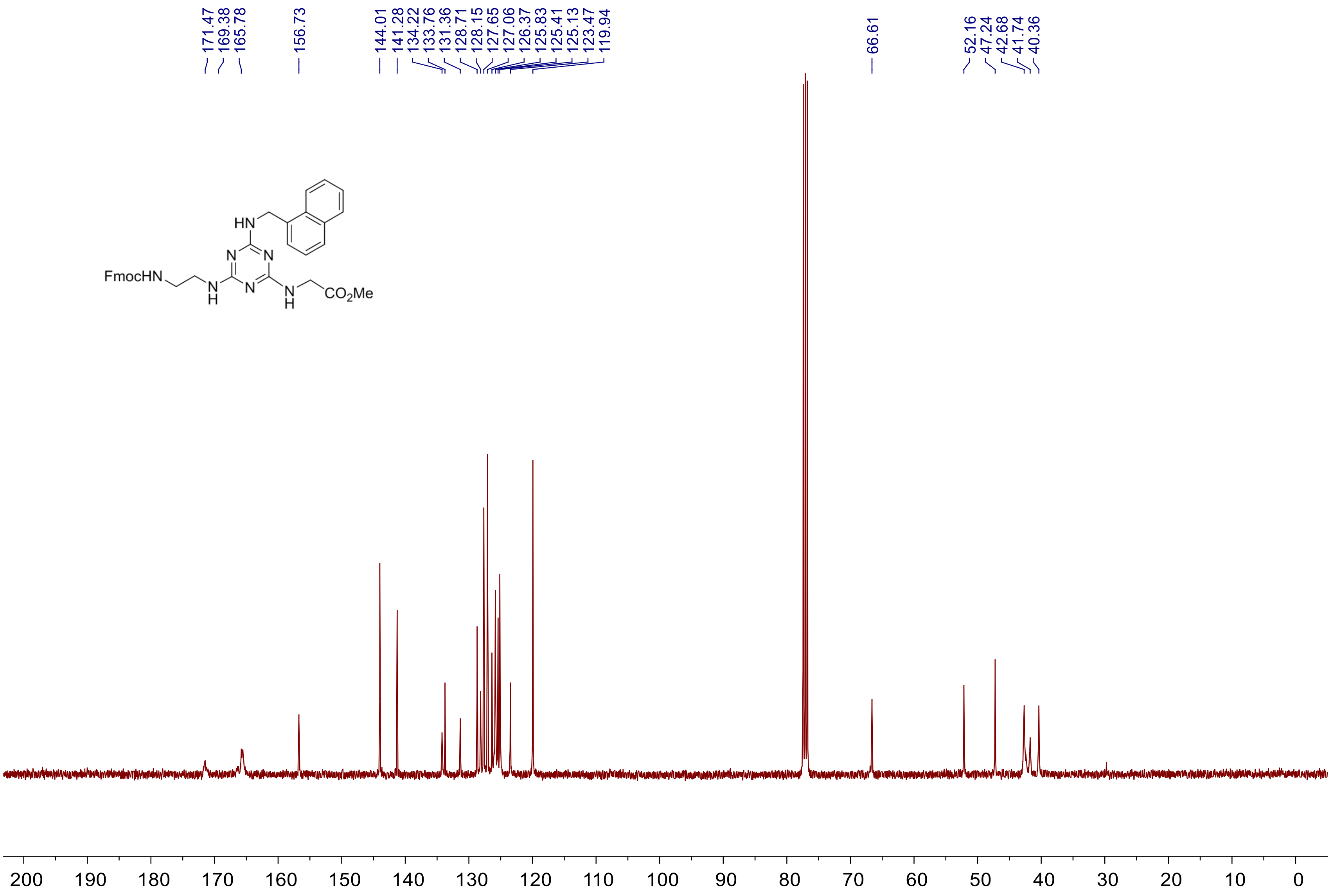

^{13}C NMR spectrum of **13c** ($CDCl_3$, 100 MHz)

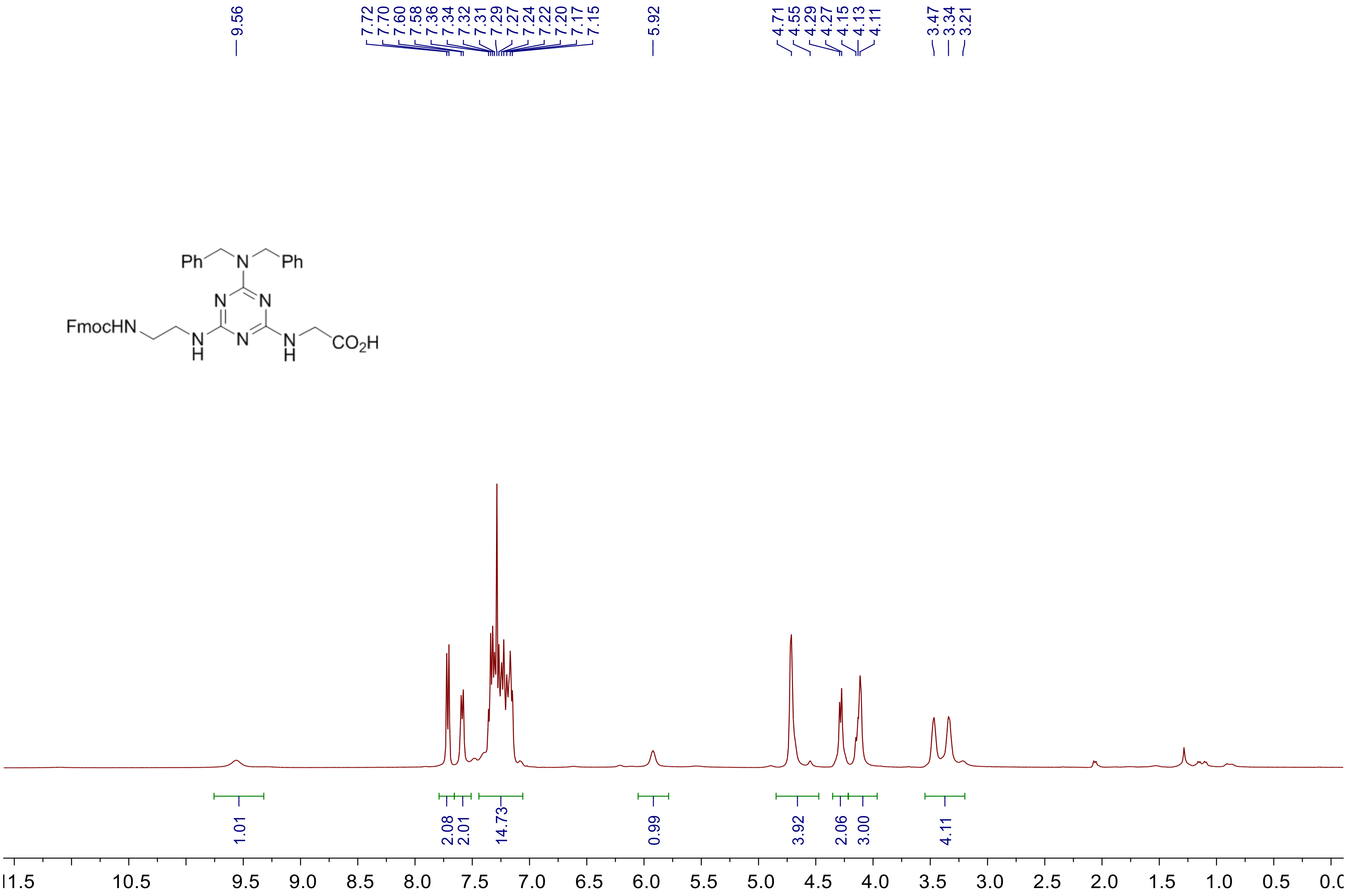

^{1}H NMR spectrum of **3a** ($CDCl_3$, 400 MHz)

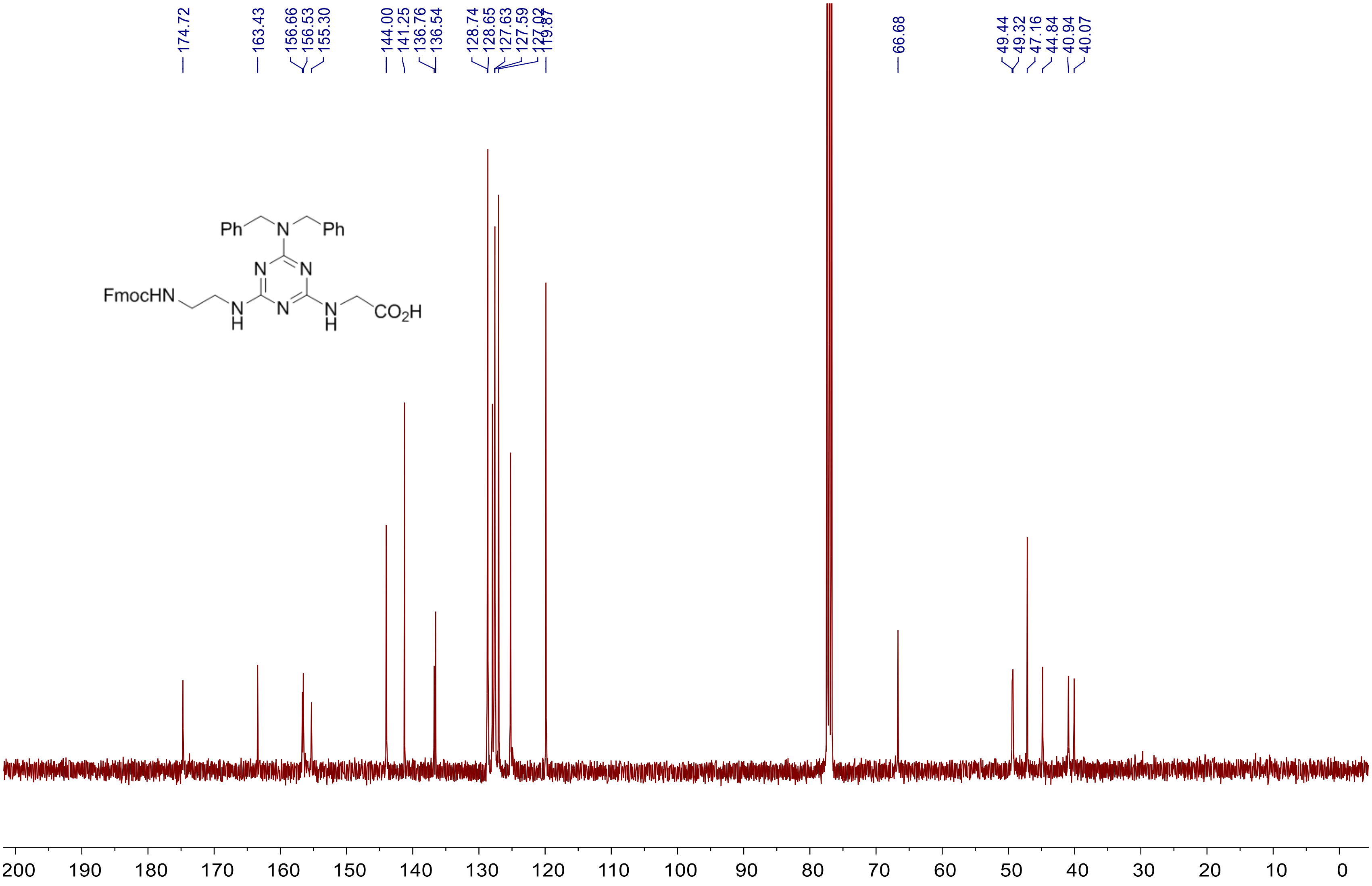

^{13}C NMR spectrum of **3a** ($CDCl_3$, 100 MHz)

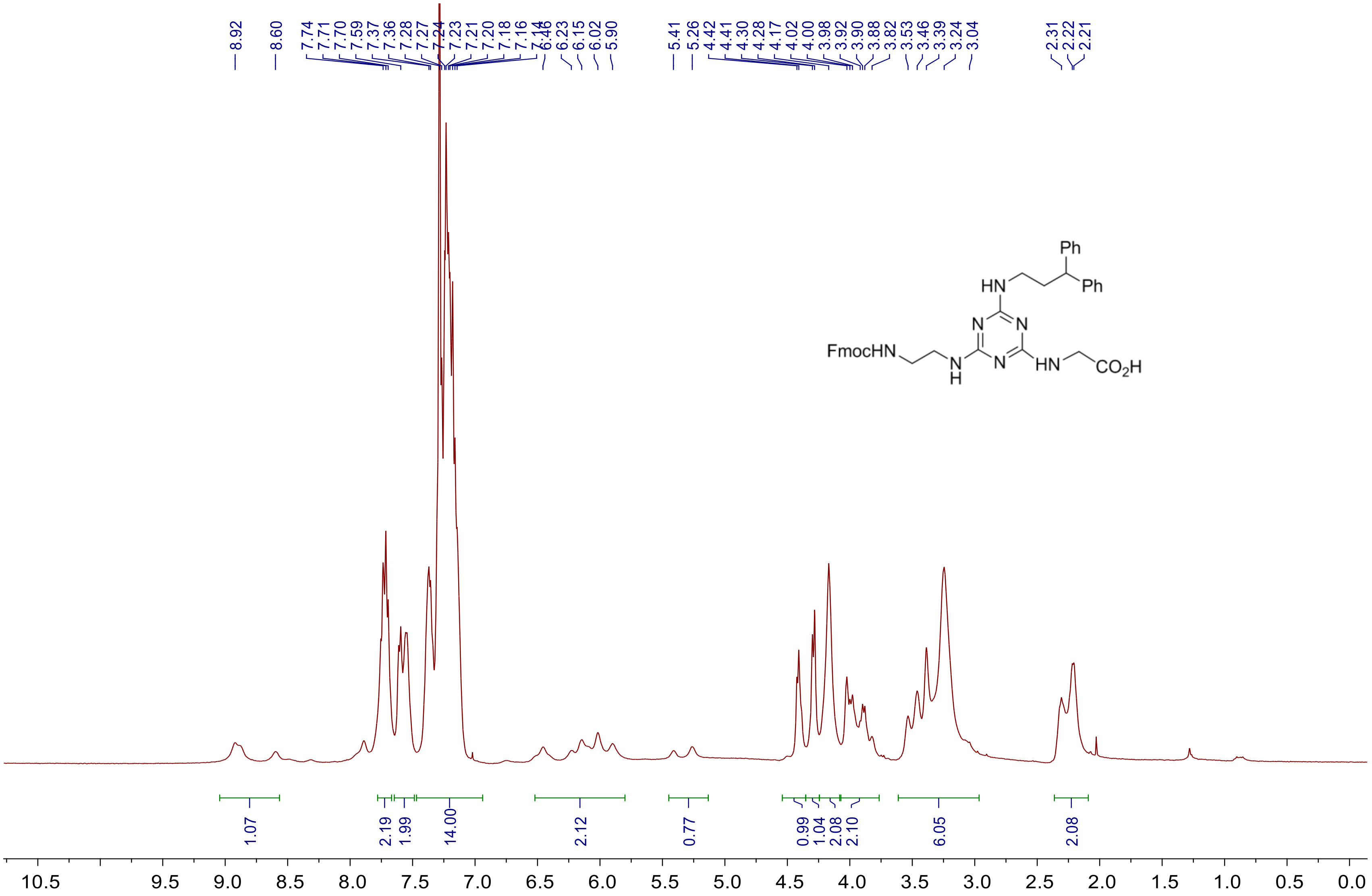

^{1}H NMR spectrum of **3b** ($CDCl_3$, 400 MHz)

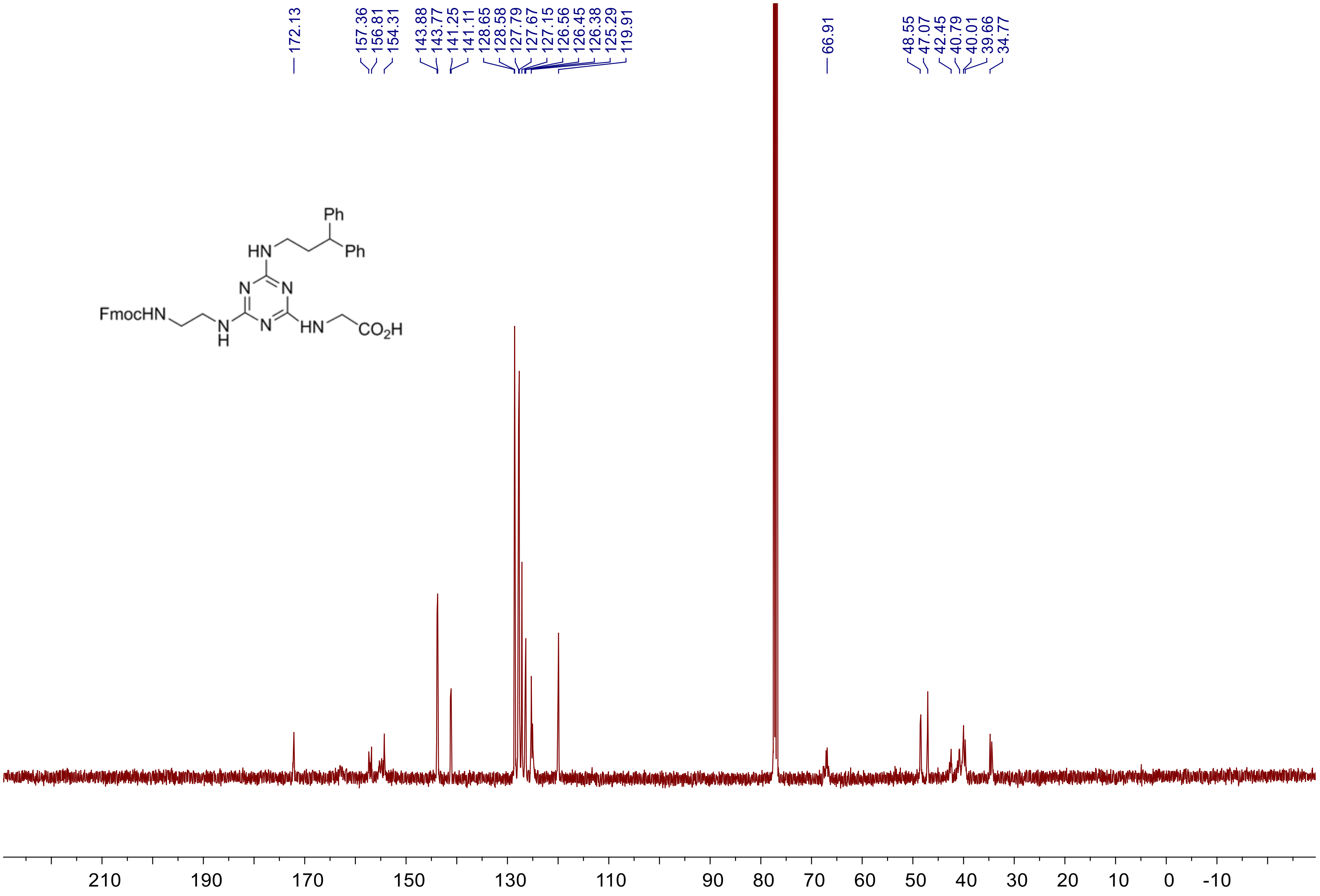

^{13}C NMR spectrum of **3b** ($CDCl_3$, 100 MHz)

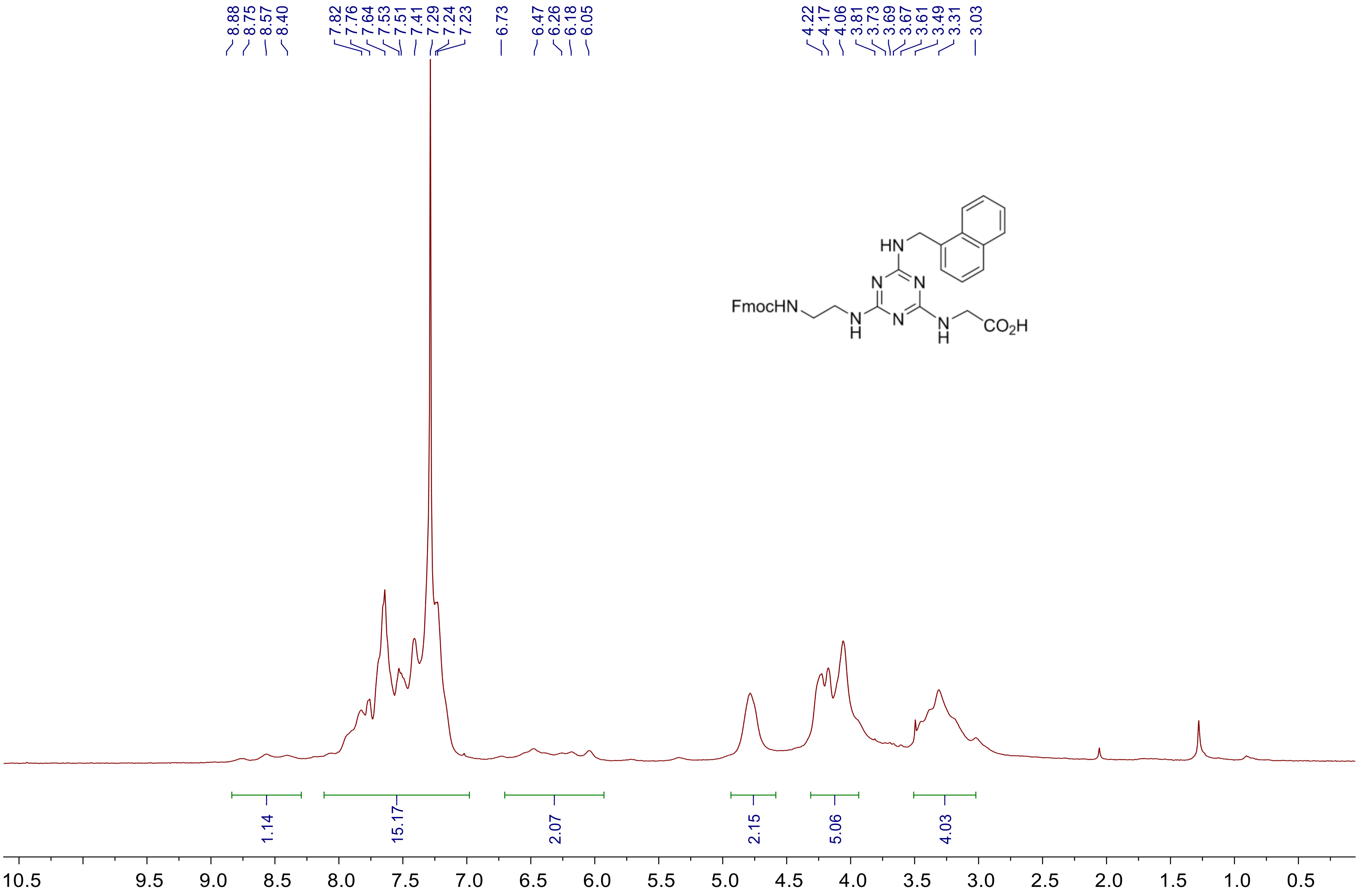

^{1}H NMR spectrum of **3c** ($CDCl_3$, 400 MHz)

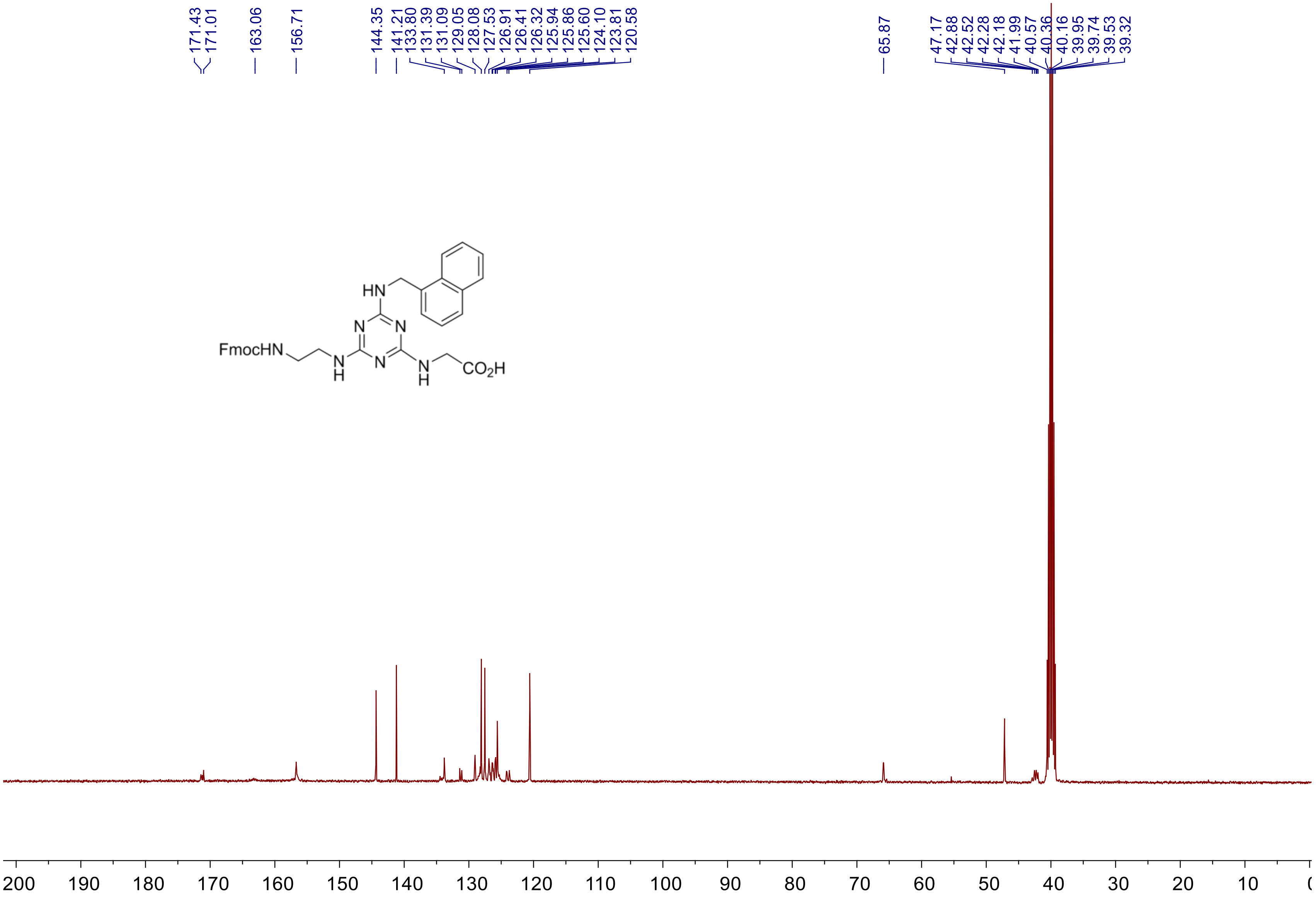

^{13}C NMR spectrum of **3c** ($CDCl_3$, 100 MHz)
